# Supplementary material for: Hospital-based caregiver intervention for people following hip fracture surgery (HIP HELPER): multicentre randomised controlled feasibility trial with embedded qualitative study in England
Source: BMJ Open. 2023 Dec 8;13(12):e073611. doi: 10.1136/bmjopen-2023-073611 (PMC10729129; doi:10.1136/bmjopen-2023-073611)
Supplement: Supplementary data [file bmjopen-2023-073611supp001.pdf]

Supplementary File 1: Clinical outcome data collected for patient-participants and caregiver-participants at baseline and four-months post-randomisation.

Patients without cognitive impairment:

- EQ-5D-5L
- Nottingham Activities of Daily Living Scale (NEADL)
- General Self-Efficacy questionnaire
- Center for Epidemiologic Studies Depression Scale (CES-D)
- Numerical rating scale (NRS) for pain (hip and whole body)
- Complications and adverse events including mortality

For all caregivers:

- EQ-5D-5L
- CES-D
- Short Sense of Competence Questionnaire for caregiver burden (SCQ-16)
- Resource Utilization in Dementia questionnaire
- Complications and adverse events including mortality
- Patient and caregiver residential status

PLUS for caregivers of patients with cognitive impairment

- EQ-5D-5L proxy
- Disability Assessment for Dementia Scale-6 (DADS-6) functional score
- Neuropsychiatry Inventory (NPI)
- Abbey Pain Scale

Supplementary File 2: Reasons for ineligibility in the HIP HELPER study across the five sites.

Site 1	Site 2	Site 3	Site 4	Site 5	Total	Screening	
37	38	11	83	31	200	Pre-admission patient did not live in the community, alone or with a friend, relative or caregiver.	Ineligible Reason
51	22	2	16	37	128	Does not have a nominated individual who will act as an informal caregiver.	
45	12	7	22	33	119	Patient has acute, unstable or terminal illness which would make participation in the rehabilitation strategies contraindicated and/or impractical.	
-	-	-	85	-	85	3+ days post op	
12	1	5	9	27	54	Patients expected by the clinical team to be discharged to a care home (residential or nursing) after their hospital admission	
15	1	1	17	9	43	Patient under age of 65 years	
7	4	-	29	1	41	Patient not willing or able to provide consent or assent depending on the level of cognitive impairment.	
8	1	6	15	8	38	Other	
8	4	-	21	-	33	Caregiver not willing or able to provide consent	
3	2	-	12	2	19	Not undergone hip fracture surgery	
-	-	-	11	2	13	Missing reason	
-	1	-	4	4	9	Participant has significant difficulties reading and/or comprehending English	
-	-	-	2	7	9	Patient under age of 65 years	
-	1	-	-	2	3	Individual caregivers unable to understand written English or have access to a translator.	
1	-	1	-	-	2	Principal (main) caregiver has AMTS score of less than 8	
					**796**	**Total ineligible**	

Supplementary File 3: Reasons for eligible participants not consenting to the HIP HELPER study across the five sites.

Site 1	Site 2	Site 3	Site 4	Site 5	Total	Not Consented	
47	26	15	43	27	158	Insufficient recruitment staff resources	Eligible but not consented
10	25	2	16	23	76	Caregiver unable to attend caregiving hospital training sessions	
15	11	6	27	11	70	Not interested in taking part in research	
6	9	1	25	14	55	Other	
21	-	3	13	1	38	Leaving the area	
3	13	1	7	4	28	Missing Reason	
19	-	-	-	1	19	Outlier ward	
-	-	-	-	-	4	Does NOT want to be randomised to receive caregiver intervention	
-	3	1	-	4	4	Persistent post-operative confusion	
-	1	1	-	-	2	Does not have a nominated individual who will act as an informal caregiver.	
-	-	-	-	-	2	Does NOT want to be randomised to receive control (usual care)	
-	-	-	1	2	2	Caregiver not willing or able to provide consent	
-	1	-	-	1	1	Participant has significant difficulties reading and/or comprehending English	
					**459**	**Total not consented**	
-	-	-	-	-	-	Post-operative complication	Not Randomised
	-	-	-	-	1	Persistent post-operative confusion	
-	-	-	-	-	-	Participant no longer wants to take part in research	
-	-	-	-	-	-	Unable to approach caregiver	
-	-	-	-	-	-	Insufficient recruitment staff resources	
2	1	-	-	1	4	Withdrawn	
					**5**	**Total not randomised**	

Supplementary File 4: Recruitment rate presented by each of the five HIP HELPER sites.

Site	Date Opened	Number of Recruited Participants											Total
		April 2021	May 2021	June 2021	July 2021	August 2021	Sept 2021	Oct 2021	Nov 2021	Dec 2021	Jan 2022	Feb 2022	
**Site 1**	07Apr2021	1	3	0	1	1	1	3	0	0			10
**Site 2**	19Apr2021		1	2	2	0	1	2	0	0	0	0	8
**Site 3**	15Jun2021				1	0	0	0	1	1	0	2	5
**Site 4**	29Jun2021				3	1	2	2	0	2	2	1	13
**Site 5**	05Aug2021					0	3	3	4	2	1	2	15
**TOTAL**		**1**	**4**	**2**	**7**	**2**	**7**	**10**	**5**	**5**	**3**	**5**	**51**

Supplementary File 5: Table illustrating the number of participant-dyads that attended intervention sessions.

	Hospital-Based Session			Telephone Booster Calls		
	**Session 1 N (%)**	**Session 2 N (%)**	**Session 3 N (%)**	**Telephone 1 N (%)**	**Telephone 2 N (%)**	**Telephone 3 N (%)**
Yes	24 (96.0)	19 (76.0)	14 (56.0)	13 (54.2)	14 (58.3)	11 (47.8)
No	1 (4.0)	6 (24.0)	11 (44.0)	11 (45.8)	10 (41.7)	12 (52.2)
Missing	0	0	0	1	1	2

Supplementary File 6: Intervention fidelity by site (shown below are those that achieved intervention fidelity).

	Site Number					All Sites
	1	2	3	4	5	
Intervention fidelity n (%)	2 (40.0)	2 (50.0)	0	3 (50.0)	5 (62.5)	12 (48.0)

N.B. fidelity = 3 in-patient sessions and >= 1 telephone calls

Supplementary File 7: Table illustrating the data completion of clinical outcome scores (baseline and 4-month follow-up).

	**Baseline (N; %)**		**4-Month Follow-up (N; %)**	
	**Intervention**	**Control**	**Intervention**	**Control**
Patient participants without Cognitive Impairment (n=41)				
EQ-5D-5L Index	20 (100)	19 (95.0)	14 (70.0)	11 (57.9)
EQ-5D-5L VAS	19 (95.0)	18 (90.0)	14 (70.0)	12 (63.2)
NEADL	15 (75.0)	17 (85.0)	5 (27.8)	9 (52.9)
GSE	19 (95.0)	19 (95.0)	11 (61.1)	10 (58.8)
CES-D	17 (85.0)	16 (80.0)	11 (61.1)	9 (52.9)
NRS pain – hip	20 (100)	18 (90.0)	12 (66.7)	10 (58.8)
NRS pain - body	19 (95.0)	19 (95.0)	12 (66.7)	10 (58.8)
Patient participants with Cognitive Impairment (n=10)				
EQ5D Proxy Index	5 (100)	5 (100)	2 (40.0)	4 (80.0)
EQ5D Proxy VAS	5 (100)	5 (100)	2 (40.0)	4 (80.0)
DADS-6 total (carer)	3 (60.0)	5 (100)	0	4 (80.0)
DADS-6 initiation (carer)	4 (80.0)	5 (100)	0	4 (80.0)
DADS-6 planification (carer)	3 (60.0)	5 (100)	0	4 (80.0)
DADS-6 performance (carer)	4 (80.0)	5 (100)	0	4 (80.0)
NPI severity (carer)	5 (100)	5 (100)	0	3 (60.0)
NPI distress (carer)	5 (100)	5 (100)	0	3 (60.0)
Abbey Pain Scale (carer)	2 (40.0)	5 (100)	0	3 (60.0)
NRS pain – hip	4 (80.0)	4 (80.0)	0	4 (80.0)
NRS pain - body	4 (80.0)	4 (80.0)	0	4 (80.0)
Caregiver participants (n=51)				
EQ-5D-5L Index	24 (96.0)	23 (92.0)	10 (47.6)	11 (50.0)
EQ-5D-5L VAS	24 (96.0)	23 (92.0)	12 (57.1)	12 (54.6)
CESD	20 (80.0)	20 (80.0)	12 (57.1)	9 (40.9)
SCQ total	23 (92.0)	20 (80.0)	12 (57.1)	11 (50.0)
SCQ recipient satisfaction	24 (96.0)	22 (88.0)	12 (57.1)	12 (54.6)
SCQ own satisfaction	23 (92.0)	21 (84.0)	12 (57.1)	12 (54.6)
SCQ consequence	24 (96.0)	23 (92.0	12 (57.1)	11 (50.0)
Patient living in own home: n (%)	24 (96.0)	23 (92.0)	11 (52.4)	11 (50.0)
Caregiver living with participant: n (%)	23 (92.0)	23 (92.0)	12 (57.1)	12 (54.5)

CESD - Center for Epidemiologic Studies Depression Scale; DAD-6 – Disability Assessment for Dementia scale – 6 item; GSE – Generalized Self-Efficacy Scale; NA – not assessed; NEADL - Nottingham Extended Activities of Daily Living scale (NEADL); NPI - Neuropsychiatry Inventory; NRS – numerical rating scale; SCQ – Short Sense of Competence questionnaire for caregiver burden; VAS – visual analogue scale
NB: Deaths prior to 4 month follow-up were assigned zero response accounting for 40% response rate for 2 participants in the patient participants with cognitive impairment responses.

Supplementary File 8: Carer Resource Utilisation in Dementia questionnaire completion statistics at baseline and 4-months

C-RUD Question	Conditional on previous question responses	Baseline		Conditional on previous question responses	4-Months	
		Intervention N=25 n (%)	Control N=25 n (%)		Intervention N=21 n (%)	Control N=22 n (%)
1. Age	No	24 (96.0)	23 (92.0)	No	12 (57.1)	12 (54.6)
2. Gender	No	24 (96.0)	23 (92.0)	No	12 (57.1)	12 (54.6)
3. Relationship to patient	No	23 (92.0)	23 (92.0)	No	12 (57.1)	12 (54.6)
4. Children living with you	No	24 (96.0)	23 (92.0)	No	12 (57.1)	12 (54.6)
5. Do you live with the patient	No	23 (92.0)	23 (92.0)	No	12 (57.1)	12 (54.6)
6. Other caregivers involved	No	23 (92.0)	23 (92.0)	No	12 (57.1)	12 (54.6)
7. Your caring contribution level	No	23 (92.0)	23 (92.0)	No	12 (57.1)	12 (54.6)
8. Sleep in last 30 days	No	24 (96.0)	21 (84.0)	No	12 (57.1)	12 (54.6)
9a. Hours per day assisting patient with tasks such as dressing in last 30 days	No	23 (92.0)	20 (80.0)	No	10 (47.6)	11 (50.0)
9b. Days assisting patient with tasks such as dressing in last 30 days	No	23 (92.0)	20 (80.0)	No	10 (47.6)	11 (50.0)
10a. Hours per day assisting patient with tasks such as shopping in last 30 days	No	23 (92.0)	20 (80.0)	No	12 (57.1)	11 (50.0)
10b. Days assisting patient with tasks such as shopping in last 30 days	No	22 (88.0)	21 (84.0)	No	12 (57.1)	11 (50.0)
11a. Hours per day supervising patient in last 30 days	No	21 (84.0)	21 (84.0)	No	11 (52.4)	12 (54.6)
11b. Days supervising patient in last 30 days	No	21 (84.0)	21 (84.0)	No	11 (52.4)	12 (54.6)
12.Work for pay	No	24 (96.0)	23 (92.0)	No	12 (57.1)	11 (50.0)
13. Stop/reduce working	Yes (N=30)	17 (100)	13 (100)	Yes (N=7)	2 (100)	5 (100)
14. Change of job/working situation	Not Assessed in Baseline C-RUD			Yes (N=7)	2 (100)	5 (100)
15. Hours of paid work per week	Yes (N=17)	7 (100)	10 (100)	Yes (N=1)	1 (100)	0
16. Hours of patient care paid per week	Yes (N=17)	7 (100)	10 (100)	Yes (N=1)	1 (100)	0
17. Hours cut for carer responsibilities in last 30 days	Yes (N=17)	7 (100)	10 (100)	Yes (N=1)	1 (100)	0
18a. Work days missed	No	6 (24.0)	11 (44.0)	Yes (N=7)	2 (100)	3 (60.0)
18b. Part work days missed	No	7 (28.0)	11 (44.0)	Yes (N=7)	2 (100)	5 (100)
19. Stop/reduce working	Not Assessed in Baseline C-RUD			Yes (N=1)	1 (100)	0

20. Admitted to hospital in last 30 days	No	24 (96.0)	23 (92.0)	Yes (N=23)	12 (100)	10 (90.9)
21. Nights in each ward in last 30 days	Yes (N=50)	23 (92.0)	23 (92.0)	Yes (N=23)	2 (16.7)	1 (9.1)
22. Hospital ER care in last 30 days	No	23 (92.0)	23 (92.0)	Yes (N=23)	12 (100)	9 (81.8)
23. Health care professional visits in last 30 days	No	24 (96.0)	22 (88.0)	Yes (N=23)	10 (83.3)	10 (90.9)
24. Current medications	No	22 (88.0)	18 (72.0)	No	8 (38.1)	8 (36.4)
25. Patient change of living accommodation since last visit	Not Assessed in Baseline C-RUD			No	12 (57.1)	11 (50.0)
26. Patient current living accommodation	No	24 (96.0)	23 (92.0)	Yes (N=0)	0	0
27. Date living change occurred	Not Assessed in Baseline C-RUD			Yes (N=0)	0	0
28. Reason for living change	Not Assessed in Baseline C-RUD			Yes (N=0)	0	0
29. Who patient lives with	No	24 (96.0)	23 (92.0)	Not Assessed in Follow-Up C-RUD		
30. Patient temporary accommodation in last 30 days	No	7 (28.0)	6 (24.0)	Yes (N=23)	8 (38.1)	5 (45.5)
31. Patient admitted to hospital in last 30 days	No	23 (92.0)	21 (84.0)	No	10 (47.6)	12 (54.6)
32. Patient nights in each ward in last 30 days	No	19 (76.0)	16 (64.0)	No	4 (19.1)	1 (4.6)
33. Patient hospital ER care in last 30 days	No	24 (96.0)	21 (84.0)	No	11 (52.4)	11 (50.0)
34. Patient health care professional visits in last 30 days	No	21 (84.0)	20 (80.0)	No	7 (33.3)	9 (40.9)
35. Patient service visits in last 30 days	No	22 (88.0)	21 (84.0)	No	8 (38.1)	11 (50.0)

* Questions are deemed completed if the main parts of the question have all been completed (e.g., checkboxes), and completion rates for some questions are conditional on previous responses.

Supplementary File 9: Table illustrating descriptive clinical outcomes presented as median and inter-quartile ranges (baseline and 4-month follow-up).

	Baseline		4-Month Follow-Up	
	**Intervention**	**Control**	**Intervention**	**Control**
Patient participants without Cognitive Impairment				
EQ-5D-5L Index	-0.10 (-0.26, 0.23)	0.05 (-0.15, 0.41)	0.60 (0.22, 0.77)	0.60 (0.34, 0.67)
EQ-5D-5L VAS	50.0 (30.0, 55.0)	47.5 (15.0, 50.0)	55.0 (30.0, 80.0)	52.5 (35.0, 80.0)
NEADL	13 (12, 20)	17(14, 21)	20 (18, 20)	16 (13, 20)
GSE	32 (26, 36)	34 (30, 40)	29.91 (5.87)	30.70 (4.83)
CES-D	23.0 (17.0, 27.0)	22.0 (18.0, 29.0)	14 (13, 18)	19 (16, 20)
NRS pain – hip	90.0 (73.5, 100.0)	82.5 (54.0, 97.0)	20.0 (4.0, 35.0)	14.5 (10.0, 45.0)
NRS pain - body	60.0 (20.0, 90.0)	55.0 (35.0, 70.0)	17.0 (1.5, 35.0)	32.5 (14.0, 64.0)
Patient participants with Cognitive Impairment				
EQ5D Proxy Index	0.15 (-0.20, 0.34)	0.22 (0.01, 0.50)	0 (0, 0)	0.33 (0.07, 0.50)
EQ5D Proxy VAS	50.00 (15.0, 50.0)	60.0 (30.0, 75.0)	0 (0, 0)	57.5 (37.5, 67.5)
DADS-6 total (carer)	2.0 (1.0, 4.00)	1.0 (1.0, 4.0)	NA	0.0 (0.0, 7.5)
DADS-6 initiation (carer)	1.5 (0.5, 3.0)	0.0 (0.0, 1.00)	NA	0.0 (0.0, 2.5)
DADS-6 planification (carer)	0.0 (0.0, 1.0)	1.0 (0.0, 2.0)	NA	0.0 (0.0, 2.5)
DADS-6 performance (carer)	1.5 (0.5, 2.5)	1.0 (0.0, 1.0)	NA	0.0 (0.0, 2.5)
NPI severity (carer)	10 (5, 12)	3 (3, 7)	NA	5 (1, 11)
NPI distress (carer)	9 (1, 11)	1 (0, 2)	NA	1 (1, 11)
Abbey Pain Scale (carer)	6.5 (3.0, 10.0)	5.0 (5.0, 6.0)	NA	5 (4, 5)
NRS pain – hip	100.0 (70.0, 100.0)	60.0(45.0, 75.0)	NA	20 (5, 50)
NRS pain - body	35.0 (20.0, 60.0)	45.0 (20.5, 65.0)	NA	51 (26, 70)
Caregiver participants				
EQ-5D-5L Index	0.80(0.71, 1.00)	1.00 (0.77, 1.00)	0.88 (0.71, 1.00)	0.77 (0.68, 1.00)
EQ-5D-5L VAS	85.0 (80.0, 92.5)	85.0 (80.0, 95.0)	82.5 (72.5, 92.5)	77.5 (67.5, 90.0)
CESD	14.0 (12.0, 19.0)	13.5 (11.5, 19.5)	16.5 (13.5, 18.0)	13.0 (12.0, 19.0)
SCQ total	60.0 (53.0, 65.0)	63.5 (60.0, 68.0)	65.5 (58.0, 74.5)	60.0(55.0, 68.0)
SCQ recipient satisfaction	17.5 (15.5, 20.0)	18.0 (16.0, 20.0)	20.0 (17.0, 20.0)	20.0 (16.0, 20.0)
SCQ own satisfaction	19.0 (17.0, 21.0)	21.0 (18.0, 22.0)	21.0 (19.5, 23.5)	18.0 (17.5, 20.5)
SCQ consequence	26.0 (21.5, 27.0)	27.0 (22.0, 28.0)	27.0 (17.5, 32.0)	24.0 (21.0, 26.0)
Patient living in own home: n=Yes (%)	23 (95.8)	21 (91.3)	11 (100.0)	10 (100.0)
Caregiver living with participant: n=Yes (%)	16 (69.6)	14 (60.9)	11 (91.7)	7 (58.3)

CESD - Center for Epidemiologic Studies Depression Scale; DAD-6 – Disability Assessment for Dementia scale – 6 item; GSE – Generalized Self-Efficacy Scale; NA – not assessed; NEADL - Nottingham Extended Activities of Daily Living scale (NEADL); NPI - Neuropsychiatry Inventory; NRS – numerical rating scale; SCQ – Short Sense of Competence questionnaire for caregiver burden; VAS – visual analogue scale

Supplementary File 10: Acceptability questionnaire (participant) by question number.

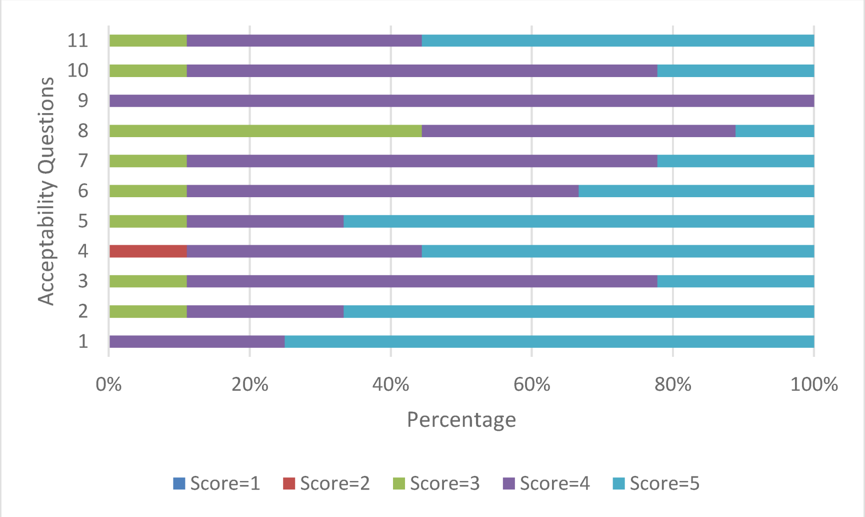

Question 1: How acceptable was the HIP HELPER in hospital training (Missing=13)
Question 2: How acceptable were the 3 HIP HELPER telephone calls? (Missing=12)
Question 3: How acceptable was the HIP HELPER Workbook? (Missing=12)
Question 4: How much effort was it to engage with the HIP HELPER in-hospital training? (Missing=12)
Question 5: How much effort did it take to engage with the HIP HELPER telephone calls? (Missing=12)
Question 6: How much effort did it take to engage with the HIP HELPER workbook? (Missing=12)
Question 7: To what extent does the HIP HELPER programme fit with your belief about recovery after a hip fracture operation? (Missing=12)
Question 8: Is the HIP HELPER programme likely to change your ability to help your friend/family member's recover after a hip fracture operation? (Missing=12)
Question 9: Does the HIP HELPER programme provide you with more confidence on your skills to help someone after a hip fracture operation? (Missing=12)
Question 10: Is it clear how the HIP HELPER intervention could help recovery after a hip fracture operation? (Missing=12)
Question 11: Did doing the HIP HELPER programme interrupt with you other priorities? (Missing=12)

Note: higher scores indicate greater acceptability.

Supplementary File 11: Acceptability questionnaire (caregiver) by question number.

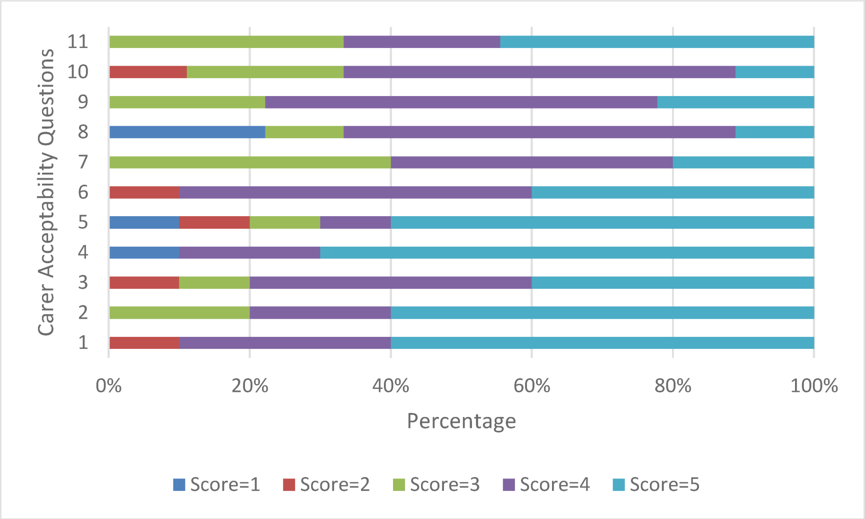

Question 1: How acceptable was the HIP HELPER in hospital training
Question 2: How acceptable were the 3 HIP HELPER telephone calls?
Question 3: How acceptable was the HIP HELPER Workbook?
Question 4: How much effort was it to engage with the HIP HELPER in-hospital training?
Question 5: How much effort did it take to engage with the HIP HELPER telephone calls?
Question 6: How much effort did it take to engage with the HIP HELPER workbook?
Question 7: To what extent does the HIP HELPER programme fit with your belief about recovery after a hip fracture operation?
Question 8: Is the HIP HELPER programme likely to change your ability to help your friend/family member's recover after a hip fracture operation?
Question 9: Does the HIP HELPER programme provide you with more confidence on your skills to help someone after a hip fracture operation?
Question 10: Is it clear how the HIP HELPER intervention could help recovery after a hip fracture operation?
Question 11: Did doing the HIP HELPER programme interrupt with you other priorities?

Note: higher scores indicate greater acceptability.

Supplementary File 12: Summary of safety outcomes at the end of the study for all participants.

**Adverse Event**	**Frequency**
Death	7
Falls	5
Joint infection	4
Increased pain (hip)	4
Increased pain (other joints)	4
Anxiety/depression	4
Atrial fibrillation	1
Deep wound infection	1
Wound infection	1
Skin integrity complication	1
PE	1
Stroke	1
Bowel obstruction	1
Barrett's oesophagus	1
**Total**	**36**

Supplementary File 13: Characteristics of qualitative investigation sample.

Person with hip fracture	Intervention	Control
N	7	3
Mean age (years)	77.85	69.33
Gender (M/F)	6 / 1	0 / 3
Ethnicity		
White British	7	3
Site (n)		
1	1	1
2	1	1
3	1	1
4	3	-
5	1	-
**Healthcare Professionals**	**Frequency**	
**Professional Role**		
Physiotherapist	4	
Occupational therapist	2	
Nurse	1	
Researcher	1	
**Site**		
1	2	
2	2	
3	1	
4	2	
5	1	
