# Supplementary material for: Hospital-based caregiver intervention for people following hip fracture surgery (HIP HELPER): multicentre randomised controlled feasibility trial with embedded qualitative study in England
Source: BMJ Open. 2023 Dec 8;13(12):e073611. doi: 10.1136/bmjopen-2023-073611 (PMC10729129; doi:10.1136/bmjopen-2023-073611)
Supplement: Author's manuscript [file bmjopen-2023-073611.draft_revisions.pdf]

BMJ Open

BMJ Open is committed to open peer review. As part of this commitment we make the peer review history of every article we publish publicly available.

When an article is published we post the peer reviewers' comments and the authors' responses online. We also post the versions of the paper that were used during peer review. These are the versions that the peer review comments apply to.

The versions of the paper that follow are the versions that were submitted during the peer review process. They are not the versions of record or the final published versions. They should not be cited or distributed as the published version of this manuscript.

BMJ Open is an open access journal and the full, final, typeset and author-corrected version of record of the manuscript is available on our site with no access controls, subscription charges or pay-per-view fees (http://bmjopen.bmj.com).

If you have any questions on BMJ Open's open peer review process please email info.bmjopen@bmj.com

BMJ Open

Hospital-based caregiver intervention for people following hip fracture surgery (HIP HELPER): multi-centre randomised controlled feasibility trial with embedded qualitative study.

Journal:	*BMJ Open*
Manuscript ID	bmjopen-2023-073611
Article Type:	Original research
Date Submitted by the Author:	21-Jun-2023

Complete List of Authors:	Smith, Toby; University of Warwick Warwick Medical School, Warwick Clinical Trials Unit; University of East Anglia, Faculty of Medicine and Health Sciences<br>Khoury, Reema; University of East Anglia, Norwich Medical School<br>Hanson, Sarah; University of East Anglia, School of Health Sciences<br>Welsh, Allie; University of East Anglia, School of Education<br>Grant, Kelly; University of East Anglia, Norwich Medical School<br>Clark, Allan; University of East Anglia, Norwich Medical School<br>Ashford, Polly-Anna; University of East Anglia, Norwich Medical School<br>Hopewell, Sally; University of Oxford, Nuffield Department of Orthopaedics, Rheumatology and Musculoskeletal Sciences<br>Pfeiffer, K; Robert Bosch Hospital, Department of Clinical Gerontology and Geriatric Rehabilitation<br>Logan, Phillipa; University of Nottingham, School of Medicine<br>Crotty, Maria; Flinders University, College of Medicine and Public Health<br>Costa, Matthew; University of Oxford, Nuffield Department of Orthopaedics, Rheumatology, and Musculoskeletal Sciences<br>Lamb, Sarah; University of Exeter, College of Medicine and Health; University of Oxford, NDORMS

Keywords:	ORTHOPAEDIC & TRAUMA SURGERY, Hip < ORTHOPAEDIC & TRAUMA SURGERY, REHABILITATION MEDICINE, Nursing Care

SCHOLARONE™
Manuscripts

I, the Submitting Author has the right to grant and does grant on behalf of all authors of the Work (as defined in the below author licence), an exclusive licence and/or a non-exclusive licence for contributions from authors who are: i) UK Crown employees; ii) where BMJ has agreed a CC-BY licence shall apply, and/or iii) in accordance with the terms applicable for US Federal Government officers or employees acting as part of their official duties; on a worldwide, perpetual, irrevocable, royalty-free basis to BMJ Publishing Group Ltd ("BMJ") its licensees and where the relevant Journal is co-owned by BMJ to the co-owners of the Journal, to publish the Work in this journal and any other BMJ products and to exploit all rights, as set out in our licence.

The Submitting Author accepts and understands that any supply made under these terms is made by BMJ to the Submitting Author unless you are acting as an employee on behalf of your employer or a postgraduate student of an affiliated institution which is paying any applicable article publishing charge ("APC") for Open Access articles. Where the Submitting Author wishes to make the Work available on an Open Access basis (and intends to pay the relevant APC), the terms of reuse of such Open Access shall be governed by a Creative Commons licence – details of these licences and which Creative Commons licence will apply to this Work are set out in our licence referred to above.

Other than as permitted in any relevant BMJ Author's Self Archiving Policies, I confirm this Work has not been accepted for publication elsewhere, is not being considered for publication elsewhere and does not duplicate material already published. I confirm all authors consent to publication of this Work and authorise the granting of this licence.

For peer review only

BMJ OPEN

TITLE PAGE

Title: Hospital-based caregiver intervention for people following hip fracture surgery (HIP HELPER): multi-centre randomised controlled feasibility trial with embedded qualitative study.

Authors: Smith TO,[1,2] Khoury R,[2] Hanson S,[2] Welsh A,[2] Grant K,[2] Clark A,[2] Ashford P-A,[2] Hopewell S,[3] Pfeiffer K,[4] Logan PA,[5] Crotty M,[6] Costa ML,[3] Lamb SE,[7] on behalf of the HIP HELPER Study Collaborators.

Affiliations

1. Warwick Clinical Trials Unit, Warwick Medical School, University of Warwick, Coventry, UK
2. Faculty of Medicine and Health Sciences, University of East Anglia, Norwich, UK
3. Oxford Trauma and Emergency Care, Nuffield Department of Rheumatology, Orthopaedics and Musculoskeletal Sciences, University of Oxford, Oxford, UK
4. Department of Geriatric Rehabilitation, Robert-Bosch-Hospital, Stuttgart, Germany
5. School of Medicine, University of Nottingham, Nottingham, UK
6. College of Medicine and Public Health, Flinders University, Adelaide, Australia
7. Exeter Medical School, University of Exeter, Exeter, UK

Corresponding Author: Professor Toby Smith, Warwick Medical School, University of Warwick, Coventry, CV1 4AL. Email: toby.smith@uea.ac.uk

For peer review only

ABSTRACT

Objectives: To assess the feasibility of conducting a pragmatic, multi-centre randomised controlled trial (RCT) to test the clinical and cost-effectiveness of an informal caregiver training programme to support the recovery of people following hip fracture surgery.

Design: Two-arm, multi-centre, pragmatic, open, feasibility RCT with embedded qualitative study.

Setting: National Health Service (NHS) providers in five English hospitals.

Participants: Community-dwelling adults, aged 60 years and over, who undergo hip fracture surgery and their informal caregivers.

Intervention: Usual care: usual NHS care. Experimental: usual NHS care *plus* a caregiver-patient dyad training programme (HIP HELPER). This programme comprised of three, one-hour, one-to-one training sessions for a patient and caregiver, delivered by a nurse, physiotherapist or occupational therapist in the hospital setting pre-discharge. After discharge, patients and caregivers were supported through three telephone coaching sessions.

Randomisation and blinding: Central randomisation was computer generated (1:1), stratified by hospital and level of patient cognitive impairment. There was no blinding.

Main outcome measures: Data collected at baseline and four months post-randomisation included: screening logs, intervention logs, fidelity checklists, acceptability data and clinical outcomes. Interviews were conducted with a subset of participants and health professionals.

Results: 102 participants were enrolled (51 patients; 51 caregivers). Thirty-nine percent (515/1311) of patients screened were eligible. Eleven percent (56/515) of eligible patients consented to be randomised. Forty-eight percent (12/25) of the intervention group reached compliance to their allocated intervention. There was no evidence of treatment contamination. Qualitative data demonstrated the trial and HIP HELPER programme was acceptable.

Conclusions: The HIP HELPER programme was acceptable to patient-caregiver dyads and health professionals. The COVID-19 pandemic impacting on site's ability to deliver the research. Modifications are necessary to the design for a viable definitive RCT.

Trial registration number: ISRCTN13270387

Data availability statement: The data that support the findings of this study are available from the corresponding author (TS) upon reasonable request. This includes access to the full protocol, anonymised participant-level dataset and statistical code.

STRENGTHS AND LIMITATIONS OF THIS STUDY

- Mixed-method approach provided useful feasibility and acceptability data.
- Assessment of diverse measures allowed evaluation of data collection for key outcome domains.
- Participant experiences and acceptability data suggest perceived value in the HIP HELPER programme.

For peer review only

- 10% of the cohort were living with cognitive impairment; none were recruited to the qualitative sub-study.
- COVID-19 pandemic affected NHS services, which impacted on study delivery.

KEYWORDS: trauma; femoral fracture; recovery; rehabilitation; domiciliary; carer; home network; RCT

WORD COUNT: 4298 manuscript; 280 abstract

For peer review only

INTRODUCTION

Hip fracture is a serious injury for older people [1]. Approximately 80,000 people aged 60 years and over experience a fragility hip fracture in the United Kingdom (UK) annually [2]. This has an estimated combined health and social cost of over £2 billion [3].

People have frequently experienced poor recovery following hip fracture [4]. The majority never return to pre-injury levels of function [3,5]. Health-related quality of life (HRQoL) is reduced and mortality is high [5,6]. Patients also often experience repeated falls. This leads to reduced independence and confidence in self-caring skills. Approximately 20% of patients who previously lived at home move into institutional care following hip fracture [7]. For those who do return home, informal caregivers who support their friend's/family member's care needs frequently experience physical and mental stress [4]. A high caregiver burden that has previously been reported by 50%, 36%, and 26% at 1-month, 3-months, and 1-year post-surgery [8] shows the multifaceted strain perceived by at least a sub-group of hip-fracture caregivers.

People after hip fracture who return home often need help. This ranges from assistance with personal activities of daily living (ADLs) such as toileting, washing, dressing and eating, to more complex tasks such as managing money, shopping and household chores [9]. Most of this required help is provided by family members or friends. Depending on the pre-fracture status of the patient, some of these informal caregivers continue in their caregiving role, others become a first-time caregiver.

Whilst informal caregivers may be willing to support their friend/family member, they frequently feel under-skilled, and have low confidence to do so [10]. A lack of information sharing, disorganised discharge planning and unclear individual roles have been identified as challenges for patients following hip fracture and their caregivers during care transitions [11]. Teaching caregiver skills to better support patients following hip fracture may improve HRQoL and independence, whilst reducing the burden of impairment for patients and caregivers [10,12].

This study aimed to assess the feasibility of conducting a pragmatic, multi-centre, randomised controlled trial (RCT) to test the clinical and cost-effectiveness of an informal caregiver training programme to support the recovery of people following hip fracture surgery.

METHODS

The study was reported to satisfy the CONSORT extension for reporting pilot and feasibility RCTs[13](. A full protocol has been published previously [14].

Study Design

This was a feasibility study comprising of a parallel, multicentre, pragmatic RCT and embedded qualitative study. The study process evaluation results are presented in this paper.

The study flowchart is presented as **Figure 1**.

Eligibility Criteria

For peer review only

Participants were recruited from orthopaedic services in five National Health Service (NHS) hospitals in England providing hip fracture surgery. We recruited adults who previously lived in the community (not institutional care), aged 60 years and over, who had undergone hip fracture surgery, could nominate an informal caregiver and provided both patient-caregiver consent to participate. Where a patient-participant did not have capacity, agreement from a consultee was sought.

We excluded people who had acute, unstable or terminal illness or were expected by the clinical team to be discharged to a care home (residential or nursing). Caregivers were ineligible if they had an Abbreviated Mental Test Score (AMTS) [15] of less than eight.

Study Treatments

Usual NHS surgical and rehabilitation care was received by both control and intervention groups [16]. Accordingly, post-hip fracture surgery, all participants received pre-discharge care including nursing, physiotherapy, occupational therapy and social service needs-assessment (where appropriate). Patients and their caregivers in the control group, did not receive the HIP HELPER programme, with no additional in-patient or out-patient caregiver training.

The HIP HELPER intervention has been previously described [14]. In brief, this was a patient-caregiver dyad training programme. The theoretical principle behind the programme is a social learning theory [17].

In practice, people randomised to the experimental group received the usual NHS care in addition to the HIP HELPER programme. The only difference between the groups was the addition of three, 60-minute, health professional-caregiver dyad HIP HELPER training sessions, performed in the hospital setting whilst the patient was an in-patient, and three follow-up telephone calls one, three and six weeks after hospital discharge. In the in-patient sessions, participants were taught about the normal recovery process, and skills in goal-setting, pacing, activity behaviour modification and stress management. They were also taught skills on manual handling, transfers, walking and how to support people with ADLs. The follow-up telephone calls aimed to re-enforce the skills developed in the face-to-face sessions, support any set-backs in recovery and to develop longer-term goals.

Each health professional who delivered the experimental intervention attended a one-day training session, which taught the components and format of the programme. To promote compliance with the treatment protocol, the Central Trial Team had regular contact with clinical team members, reviewing the first HIP HELPER sessions for intervention fidelity and held monthly meetings regarding study processes.

Data Collection

At the time of enrolment, sites checked eligibility and recorded demographic characteristics in the screening log. Baseline assessments were undertaken after consent was obtained, prior to randomisation. Data collected at baseline included: hospital admission, age, sex, ethnicity, height, weight, patient cognitive impairment assessed using the AMTS [15], past medical history, American Society of Anaesthesiologists (ASA) grade [18], side of hip fracture, operative procedure and hip fracture classification. Caregiver demographic data collected included: relationship of caregiver to patient, age, sex, ethnicity, past medical history, AMTS [15], whether they lived with the patient, employment status and experience of being a caregiver (for this patient and/or for another person).

Participants were followed-up at four months post-randomisation.

Outcome Measures

For peer review only

To answer our feasibility objectives, we assessed:

1. Recruitment feasibility – by screening log data on: number of potential participants and their caregivers assessed for eligibility, including reasons for exclusion/non-participation, and consented to be randomised; timing and location of approach and consent.

2. *Intervention acceptability* – by qualitative interviews with participants and health professionals; acceptability questionnaire, study attrition at the intervention phase.

3. *Intervention fidelity (healthcare professionals)* – by intervention log data on: HIP HELPER session duration, frequency, location (orthopaedic/orthogeriatric ward, rehabilitation ward or other); Quality Assurance (QA) to monitor HIP HELPER programme delivery.

4. *Intervention fidelity (caregivers)* – by caregiver HIP HELPER programme intervention logs; qualitative interviews.

5. *Randomisation acceptability* – by screening logs, eligibility assessment logs and consent forms; participant attrition; qualitative investigation.

6. *Risk of contamination* - by HIP HELPER programme log data including: QA monitoring visit checklists; delegation logs; qualitative interviews with health professionals.

7. *Completeness of outcome measures* - by completion rates (baseline and four months post-randomisation). Outcome measures collected are included in **Supplementary File 1**.

Randomisation and blinding

Randomisation was at the patient-caregiver dyad level (1:1 experimental and control groups) by stratification for: hospital and the presence of patient cognitive impairment (AMTS[15] < or ≥ 8 points). Sites team members performed randomisation post-baseline data collection. Allocation was concealed prior to randomisation. Randomisation was computer generated, performed by site team members on a secure, online programme, centrally administered by an independent programmer at the Norwich CTU (NCTU). The randomisation sequence was generated by NCTU programmers, tested by the trial statistician.

Due to the participatory nature of the intervention, blinding participants or the site team was not possible. Senior research team members were blinded to treatment allocation for the duration of the study.

Sample Size

We aimed to recruit 120 participants (60 patients; 60 caregivers). This was considered sufficient to answer our feasibility objectives and assess the *a priori* progression criteria (**Table 1**).

Data Analysis and Progression Criteria

Consent rates, recruitment rates, attrition, missing data rates and intervention fidelity were reported as proportions with 95% confidence intervals (CIs) presented for consent and recruitment rates. The analysis of clinical outcome measures was descriptive, reported as means and standard deviations (SD) or medians and interquartile ranges (IQR) and numbers and percentages for binary and categorical variables. No formal statistical testing was undertaken.

For peer review only

A 'traffic light' system was used as a guide for progression to a definitive trial [19]. The progression criteria were centred around recruitment, retention, intervention fidelity and contamination.

Study Monitoring

A Trial Oversight Committee (TOC) was appointed to independently review data on safety, protocol adherence and study processes.

Patient and Public Involvement

Patient involvement began during protocol development and continued throughout the study. One patient-member (not enrolled in the study) attended TOC meetings. They provided insights into the study conduct, particularly on data collection processes and helped interpret the findings to inform the study's dissemination phase.

Participants who expressed an interest in receiving information on the findings were provided with this.

Embedded Qualitative Study

The aim of the embedded qualitative investigation was to assess the acceptability of the HIP HELPER programme and the research design from the perspective of caregiver dyads and health professionals. Its design was guided by MRC guidance for evaluating complex interventions [20-22], with the intention of understanding contextual factors influencing implementation, theorising how the HIP HELPER programme may work in practice and identifying key uncertainties to enable the programme and research design to be refined.

Six weeks after hospital discharge, caregiver dyads were invited, via a telephone call, to participate in an in-depth, semi-structured interview. They were purposively sampled by age, ethnicity, pre-fracture disability (Nottingham ADL scale (NEADL)[23]), level of cognitive health (AMTS [15]), Disability Assessment for Dementia Scale-6 [24]) and study site. For health professionals, we invited for interview those who had completed the HIP HELPER programme with at least one caregiver-dyad. This sample was purposively sampled by site location and clinical background, to ensure representation across physiotherapy, nursing and occupational therapy professions. All interviews were conducted virtually using Microsoft Teams or telephone. Our topic guide was informed by the MRC guidelines [21,22] and Sekhon's framework of acceptability [25]. All interviews were conducted by the same researcher (AW), an experienced post-doctoral, female, qualitative researcher. AW had no role in recruitment to the study nor intervention delivery. Interviews were audio-recorded, anonymised and transcribed verbatim.

Our analysis took a two-stage approach. Firstly deductive, to assess the quality of implementation and identify contextual factors using the MRC frameworks as a guide [21,22]. An inductive approach further explored participant's experiences and reflections on the intervention from a caregiver dyad perspective. Analysis was independently conducted by one researcher (AW) and then themed with an additional two (SH, TS) using Reflexive Thematic Analysis, whereby the highly contextual nature of the data was acknowledged [26,27].

RESULTS

For peer review only

Patient characteristics and treatment

As a result of disruption on NHS services caused by the COVID-19 pandemic, we recruited 102 participants (51 patients; 51 caregivers) from April 2021 to February 2022.

A summary of the patient-cohort characteristics is presented in **Table 1**. Seventy-four percent (37/50) were female, with a mean age of 81.4 years in the intervention group and 77.6 years in the control group. In total, 94% (47/50) were white British or Irish. Ten patient-participants (five per group) had a AMTS [15] of less than eight, indicating cognitive impairment at baseline. The median length of hospital stay was 15 days (IQR: 10, 19) in the intervention group and 11 days in the control group (IQR: 8, 17). As **Table 1** demonstrates, people with hip fracture in the intervention group were older, with more medical co-morbidities and more frequently presented with intra-trochanteric fractures.

A summary of the caregiver-participant characteristics is presented in **Table 2**. Fifty-three percent (36/50) of the cohort were female. Mean age of caregivers in the intervention group was 66 years and 58 years in the control group. Caregivers were most frequently patient-participant's children (53%; 26/50) or a spouse (37%; 18/50). Most caregivers were not working (65%; 32/50); 20% (10/50) were in full-time work.

Feasibility outcomes

The outcomes of the progression criteria traffic-light assessment are presented in **Table 3**.

Recruitment, retention and randomisation acceptability

The CONSORT flow-chart is presented in **Figure 1**. As this illustrates, 1311 potential participants were screened. Of these, 515 (39%; 95% CI: 37% to 42%) were eligible, with 56/515 (11%; 95% CI: 8% to 13%) of eligible participant-dyads consented to participate. Five participant-dyads were withdrawn prior to randomisation. A summary of reasons for being ineligible or being eligible but not consenting are presented in **Supplementary File 2** and **Supplementary File 3**. Recruitment activity per-site is presented in **Supplementary File 4**.

At four-month follow-up, 43/51 participant-dyads (86%) (21 intervention, 22 control) remained in the study. Six participants died, one withdrew without reason; in each instance the complete dyad were withdrawn. At four-months, there were eight patient-participants with cognitive impairment, 35 without cognitive impairment. Whilst the groups were largely comparable at baseline, the experimental group were slightly older, with a greater number of co-morbidities and a higher frequency of intra-trochanteric fracture (**Table 1**).

Intervention fidelity (health professionals)

Supplementary File 5 illustrate the delivery of the hospital-based and telephone-based HIP HELPER sessions. As summarised in **Supplementary File 6**, 12/25 participant-dyads (48%) of participant-dyads received the minimal compliance level of all three HIP HELPER in-patient sessions and one telephone call. Reasons for non-compliance were: insufficient staff to deliver the intervention due to staff redeployment and interruption of service provision or visiting of participants due to the COVID-19

For peer review only

pandemic (n=2); patient-participant transferred from a unit (n=2); death (n=4); treatment discontinuation (n=4); or did not answer the telephone (n=3).

Table 4 illustrates that all components of the HIP HELPER programme were delivered during testing. Components which were most frequently delivered were: explanation on recovery expectations (96%; 23/25), goal-setting (92%; 22/25) and pacing and behaviour modification (92%; 22/25). Less frequently delivered components were the more functionally-demanding activities such as washing, dressing and stair and car transfers (38%; 9/25 each).

Intervention fidelity (caregivers)

Only two caregiver-participants returned their caregiver log. Accordingly, there were insufficient data to permit robust assessment of intervention fidelity from the caregiver perspective. This was therefore not analysed.

Contamination

From the qualitative investigations, case report forms for treatment received, protocol deviation reports and delegation logs of treating health professionals, there was no evidence of between-group intervention contamination.

Outcome Data Response Rate

There was limited difference in the completion of the caregiver-participant outcomes at baseline or four months in either group (**Supplementary File 7; Supplementary File 8**). However, there was a notable difference in outcome completion at four months for patients with cognitive impairment and their caregivers. Whilst 80% of caregivers in the control group completed the majority of outcomes, only two caregivers of people with cognitive impairment completed the outcomes (EQ-5D proxy only) in the intervention group. Patient-participants in the intervention group reported a higher response rate to all outcomes at four months (**Supplementary File 7**). Of the outcomes measured, NEADL[23] demonstrated lowest response at four months (Intervention 28%; Control 53%).

Clinical Outcomes

Supplementary File 9 illustrates the descriptive clinical outcomes presented as median and IQRs for baseline and four-month follow-up. Between-group differences should be interpreted with caution given the level of missing data in both groups (**Supplementary File 7**), a potential baseline difference between the groups for age, presented co-morbidities and fracture type (**Table 1**) and underpowered analyses.

No participant, from either group, experienced a related adverse event or serious adverse event. A summary of the patient-caregiver reported adverse events is presented in **Supplementary File 10**.

Intervention acceptability questionnaire data indicated the HIP HELPER programme was regarded as acceptable by people with hip fracture (**Supplementary File 11**) and caregivers (**Supplementary File 12**).

For peer review only

Qualitative Study

Fourteen caregiver dyads were invited to be interviewed. Ten agreed to participate (Intervention: seven participants; control: three participants). All eight health professionals approached, agreed to be interviewed. **Supplementary File 13** summarises the patient-caregiver's and health professional's characteristics.

Our findings are grouped into three main themes: context, intervention delivery and study procedures.

Context

This study was conducted during the COVID-19 pandemic. Patient visitor policies and restrictions were in place. Most caregiver dyads suggested that the opportunity to visit their friend/relative was a main driver for participation.

> *"This trial helped me visit **** once or twice more during her stay in hospital than I would have been allowed to." (*Caregiver 1, Intervention Group, Male, Site 4*)*

For health professionals, allocating visiting time slots created a further challenge of obtaining consent.

> *"With visiting times, we're making sure it's the right carer coming in because it might not be them that have the slots booked that week."* (Occupational Therapist, Female, Site 1).

Changes in visitor policies/restrictions would need to be considered for any future trial.

From the perspective of health professionals, one of the most common reasons for non-participation was attributed to perceived burden on caregivers.

> *"They say, 'oh no that seems like a lot for my daughter to take on or that seems a lot for husband to do they already do enough, or I don't think they'd manage that'. So, it's that perception that they don't want to put any more burden on someone. Seems to be the main reason we find."* (Occupational Therapist, Site 1)

> *"Caregivers who work full-time or spouses who are too frail to make it into hospital just can't."* (Research Physiotherapist, Male, Site 5)

For this study, there was the added complexity of dyad recruitment, as reflected in this comment:

> *"I think getting both the caregiver and the patient consent is a bit of a headache."* (Occupational Therapist, Female, Site 1)

Initially, when health professionals approached the person with hip fracture, a key reason for decline was concerns about feelings of burden on their caregivers. Staff felt recruitment was more successful when the potential caregiver was approached first.

Intervention delivery

For peer review only

Participants perceived the workbook to be helpful in giving a sense of tangible timeframes for recovery. Goal-setting was seen as helping in pushing people out of their comfort zones and allowed them to reflect on progress.

> *"Made us gauge our progress and that he wanted our response to what goals we had. And as I said, going back through it each time we read another page, you realise that we've upped the goal and how far we've progressed"* (Caregiver, Intervention Group, Male, Site 4)

Areas of workbook refinement were identified, such as the volume of information included. Importantly some felt that the workbook did not reflect the life circumstances of younger participants and those still in work.

> "*Like, say, when I did it [the case studies], I was like how am I gonna drag this out for an hour with a patient who can just about get bed to chair*." (Physiotherapist, Male, Site 5)

The follow-up phone calls were seen as helpful. For health professionals, this addition added a rewarding element to their role.

> *"Telephone calls have been really useful. Especially for me because I work on inpatients where don't often get time to follow-up a patient, see how they are, see if there's any concerns. I suppose, from a development point of view, knowing what has worked and what hasn't."* (Physiotherapist, Male, Site 2)

For the patient-caregiver dyads, telephone calls provided encouragement and reassurance to maintain and progress activities.

> *"Reassured me that I'm doing the right things and where I should expect to be."* (Person with Hip Fracture, Intervention Group, Female, Site 4)

Participants perceived value in the telephone calls particularly in navigating additional services and support after discharge. They expressed this would have been challenging without this follow-up.

<u>*Study procedures*</u>

Respondents repeatedly acknowledged the perceived burden of completing the outcome measures.

> *"It would be a lot for say, the husband to stay at home, trying to fill in 15 pages, when the wife is not there and then they have to try to adjust living by themselves and with all this happening."* (Nurse Practitioner, Female, Site 2)

The outcome measures evaluated patient-caregiver outcomes from a biopsychosocial perspective [28]. Accordingly, some patient reported outcomes posed questions regarding an individual's ability to cope with the physical and psychosocial challenges of trauma. For some respondents, this was reported as emotionally difficult.

> *"So, when you were doing it [questionnaires] with the carer, there was a couple of times where it was uncomfortable. They might not want to say that I have low in mood in front of someone. It was quite upsetting for the carer to divulge that information with you, or to bring up things from their past as well."* (Physiotherapist, Male, Site 3)

For peer review only

DISCUSSION

The findings from this feasibility study indicate that the HIP HELPER intervention was acceptable to patients, informal caregivers and health professionals but the trial design requires further development to ensure feasibility. Modifications should be made on promoting intervention adherence, prioritising outcome measures to test future effectiveness of data completeness and exploring strategies to support the recruitment of patients with cognitive impairment.

The completion of data from the outcomes at four months was lower than anticipated. This was particularly for participants with cognitive impairment. The qualitative study indicated that participants found the number of outcome measures challenging to complete and future study could better discern what outcome measures are important to people following hip fracture and their caregivers [29]. Streamlining should be made to determine the outcomes which are most valuable to participants and clinical commissioners. A second major modification relates to the acceptability of randomisation. Only 11% of eligible participants were randomised. The qualitative study indicated this may have been because patient-participants did not wish to 'burden their caregiver' with the study when they were initially approached and so declined participation. We originally designed the study approach pre-COVID 19 with an initial approach occurring when both patient and caregiver were together, during visiting hours. This was to facilitate a collaborative decision between the dyad, rather than one member deciding participation. However, due to COVID-19 restrictions on visiting, this was not possible. The qualitative study indicated that the originally planned approach should have been more successful. We recommend that both members of the dyad should be approached simultaneously in future trials, to mitigate such low conversion to randomisation.

A written, information guide about rehabilitation, recovery goals and caregiver responsibilities in the home has been previously reported as valuable to other populations [30]. The findings of this study indicate that whilst the addition of this intervention was beneficial, the HIP HELPER workbook received mixed views from participants. The level of detail, degree of context and order of material covered in the HIP HELPER workbook was considered by many participants as too great. Equally the qualitative findings suggested that the current materials were not representative of all patients and caregivers, most notably younger people who sustain a hip fracture. Further patient and public consultation with the research and clinical hip fracture community is needed to modify this workbook and associated digital offerings of this material.

Approximately 40% of people who sustain a hip fracture present with dementia [31]. Whilst previous authors have acknowledged potential challenges in recruiting people with dementia to drug trials [32], no studies have explored recruitment expectations or strategies to address low recruitment to non-pharmacological interventions [33]. We anticipated recruiting 20 patient-participants with cognitive impairment. In total, 10 participants were recruited with mild cognitive impairment. The qualitative findings suggest that offering further support to research site members who approach patients with cognitive impairment and their caregivers, to promote skills conveying study information, may be beneficial. Furthermore, given the poor response rate in four-month outcome data for these participants, consideration on the appropriateness of the current instruments used and model of delivery of outcome battery for people with cognitive impairment and their caregiver, should be considered in future trials of this population [34].

Previous evidence suggests that health professionals have been inflexible about people with hip fracture and their caregivers to discuss care plans, when these do happen [35-37]. The qualitative

For peer review only - http://bmjopen.bmj.com/site/about/guidelines.xhtml

study highlighted that those participants who received the HIP HELPER programme appreciated the contact and opportunity to explore skills and knowledge for early recover and caregiver support following hip fracture. The addition of the telephone calls was reported as offering beneficial, additional, post-discharge support in a flexible approach. However, intervention fidelity was lower than anticipated. Unfortunately, it is difficult to separate the challenges which COVID-19 placed on research conduct and service provision and challenges in delivering the HIP HELPER programme in a non-health crisis. Sites were challenged in delivering the intervention due to staffing, patient transfers, visiting restrictions and earlier than planned discharges. This impacted on fidelity of the 'full' HIP HELPER programme to all participants. Deeper exploration on modification to intervention delivery and what components are 'core' ingredients to the programme to estimate compliance thresholds would be warranted.

This study presented with strengths and limitations. A notable strength was the ability to recruit over 100 participants from five NHS organisations during the 2020 COVID-19 pandemic. Whilst a short-fall of 10 participant-dyads, given the challenges in managing site opening and research conduct during the COVID-19 pandemic, the ability to undertake this was considered a success. In the absence of COVID-19 (or such like) restrictions on visiting, patient flow and research activity in NHS settings, we would anticipate that this impact would be negated in future trials of this population. Secondly, although we planned to assess whether caregivers adopted their caregiving knowledge from the intervention into the home environment, only two participants returned these data. This was a major limitation and resulted in an inability to answer an *a prior* progression criterion. The findings from the qualitative study and acceptability questionnaires may suggest carry-over of the intervention into practice. However, we acknowledge that this does not offer the granularity of detail which the original caregiver log would have conveyed. Finally, the follow-up rates and data competition for clinical outcomes were low. Accordingly, it was not possible to confidently assess for a signal of efficacy in the experimental intervention. Further modifications in what and how clinical outcome data are collected should be considered as part of the following work to improve the feasibility of this trial design.

CONCLUSIONS

The HIP HELPER programme was acceptable to participants and health professions. Further modifications to the trial design are needed to ensure feasibility. These findings will form the basis of reflection and refinement to the trial design.

For peer review only

ACKNOWLEDGEMENTS AND DECLARATIONS

Collaborators: The HIP HELPER Study Collaborators: Penny Clifford (Norfolk, PPI Representative), Lis Freeman (Norfolk, PPI Representative), Rene Gray (Principal Investigator – James Paget University Hospital NHS Trust), Yan Cunningham (Principal Investigator – City Hospitals Sunderland NHS Foundation Trust), Sarah Langford (Principal Investigator - Northumbria Healthcare NHS Foundation Trust), Dr Mark Baxter (Principal Investigator - University Hospital Southampton NHS Foundation Trust), Jessica Pawson – (Principal Investigator - Barts Health NHS Trust), Melissa Taylor (James Paget University Hospital NHS Trust), Anna Mellows (James Paget University Hospital NHS Trust), Kate Lacey (James Paget University Hospital NHS Trust), Alex Herring (City Hospital Sunderland NHS Foundation Trust), Diane Williams (Northumbria Healthcare NHS Foundation Trust), Anna Cromie (Northumbria Healthcare NHS Foundation Trust), Gail Menton (Northumbria Healthcare NHS Foundation Trust), Warren Corbett (University Hospital Southampton NHS Foundation Trust), Helen Jowett (University Hospital Southampton NHS Foundation Trust), Vishwanath Joshi (Barts Health NHS Trust), Maninderpal Matharu (Barts Health NHS Trust), Maria Baggot (University Hospital Southampton NHS Foundation Trust),and David Barker (University Hospital Southampton NHS Foundation Trust).

Oversight Committee Membership: TOC Members: Associate Professor Susan Dutton (Chair; University of Oxford, UK), Professor Opinder Sahota (University of Nottingham, UK), Dr Katie Sheehan (Kings College London, UK).

Authors' Contributors: TS, SHo, SHa, PA, KP, PL, MCo, SEL, MCr researched the topic and devised the study. TS, SHa, PA, RK, AC, SHo, AW, KP, PL, MCo, SEL, MCr provided the first draft of the manuscript. AC provided statistical oversight. TS, SHa, PA, RK, AC, AW, SHo, KP, PL, MCo, SEL, MCr contributed equally to manuscript preparation. TS acts a guarantor.

Funding Statement: This project is funded by the National Institute for Health and Care Research (NIHR) under its Research for Patient Benefit (RfPB) Programme (Grant Reference Number NIHR200731). SL role in this study was supported by the National Institute for Health and Care Research Exeter Biomedical Research Centre. The views expressed are those of the author(s) and not necessarily those of the NIHR or the Department of Health and Social Care.

Competing Interests Statement: None declared.

Disclaimer: MCo and SHo are supported by the National Institute for Health Research (NIHR) Oxford Biomedical Research Centre (BRC). SL is supported by the National Institute for Health and Care Research Exeter Biomedical Research Centre. The views expressed are those of the author(s) and not necessarily those of the NHS, the NIHR or the Department of Health and Social Care.

Ethics Approval: North East - Newcastle & North Tyneside 1 Research Ethics Committee (20/NE/0213) Date: 16 March 2021.

Trial Sponsor: University of East Anglia (Research Sponsor, Research and Innovation Services, The Registry, University of East Anglia, Norwich, NR4 7TJ; Email: researchsponsor@uea.ac.uk) The views expressed are those of the author(s) and not necessarily those of the sponsor.

Provenance and Peer Review: Not commissioned; externally peer reviewed.

Data Sharing: The data that support the findings of this study may be available from the corresponding author (TS) upon reasonable request. This includes access to the full protocol, anonymised participant-level dataset and statistical code.

For peer review only

Patient consent for publication: Not required.

For peer review only

FIGURE AND TABLE LEGENDS

Table 1: Demographic characteristics of the patient participants at baseline.

Table 2: Demographic characteristics of the caregiver participants at baseline.

Table 3: Progression criteria traffic-light summary table.

Table 4: Table illustrating the frequency to-which the components of the HIP HELPER intervention were delivered to participants.

Figure 1: CONSORT diagram reporting the flow of patient-caregiver participants in the HIP HELPER study.

For peer review only

Supplementary File 1: Clinical outcome data collected for patient-participants and caregiver-participants at baseline and four-months post-randomisation.

Supplementary File 2: Reasons for ineligibility in the HIP HELPER study across the five sites.

Supplementary File 3: Reasons for eligible participants not consenting to the HIP HELPER study across the five sites.

Supplementary File 4: Recruitment rate presented by each of the five HIP HELPER sites.

Supplementary File 5: Table illustrating the number of participant-dyads that attended intervention sessions.

Supplementary File 6: Intervention fidelity by site (shown below are those that achieved intervention fidelity).

Supplementary File 7: Table illustrating the data completion of clinical outcome scores (baseline and 4-month follow-up).

Supplementary File 8: Carer Resource Utilisation in Dementia questionnaire completion statistics at baseline and 4-months.

Supplementary File 9: Table illustrating descriptive clinical outcomes presented as median and inter-quartile ranges (baseline and 4-month follow-up).

Supplementary File 10: Acceptability questionnaire (participant) by question number.

Supplementary File 11: Acceptability questionnaire (caregiver) by question number.

Supplementary File 12: Summary of safety outcomes at the end of the study for all participants.

Supplementary File 13: Characteristics of qualitative investigation sample.

REFERENCES

1. National Hip Fracture Database 2023. Assessment benchmark summary 2022. Accessed: 16th February 2023. Available at: https://www.nhfd.co.uk/tables
2. Mitchell P, Bateman K. Dementia, falls and fractures. Integrated approaches to improve quality and reduce costs. UK: Novartis; 2012.
3. Dyer SM, Dyer SM, Crotty M, Fairhall N, Magaziner J, Beaupre LA, Cameron ID, Sherrington C; Fragility Fracture Network (FFN) Rehabilitation Research Special Interest Group. A critical review of the long-term disability outcomes following hip fracture. *BMC Geriatr* 2016;**16**:158.
4. Lawler K, Taylor NF, Shields N. Involving family members in physiotherapy for older people transitioning from hospital to the community: a qualitative analysis. *Disabil Rehabil* 2015;**37**:2061-9.
5. Johnell O, Kanis JA. An estimate of the worldwide prevalence, mortality and disability associated with hip fracture. *Osteopor Int* 2004;**15**:897-902.
6. Griffin XL, Parsons N, Achten J, Fernandez M, Costa ML. Recovery of health-related quality of life in a United Kingdom hip fracture population. The Warwick Hip Trauma Evaluation--a prospective cohort study. *Bone Joint J* 2015;**97-B**:372-82.
7. Nurmi I, Narinen A, Lüthje P, Tanninen S. Cost analysis of hip fracture treatment among the elderly for the public health services: a 1-year prospective study in 106 consecutive patients. *Arch Orthop Trauma Surg* 2003;**123**:551-4.
8. Ariza-Vega P, Ortiz-Piña M, Kristensen MT, Castellote-Caballero Y, Jiménez-Moleón JJ. High perceived caregiver burden for relatives of patients following hip fracture surgery. *Disabil Rehabil* 2017;**16**:1-8
9. Wimo A, Wetterholm AL, Mastey V, Winblad B. Evaluation of the resource utilization and caregiver time in Anti-dementia drug trials - a quantitative battery. In: Wimo A, Karlsson G, Jönsson B, Winblad B (eds). The Health Economics of dementia, 1998. Wiley's, London, UK
10. Saletti-Cuesta L, Tutton E, Langstaff D, Willett K. Understanding informal carers' experiences of caring for older people with a hip fracture: a systematic review of qualitative studies. *Disabil Rehabil* 2018;**40**:740-50.
11. Asif M, Cadel L, Kuluski K, Everall AC, Guilcher SJT. Patient and caregiver experiences on care transitions for adults with a hip fracture: a scoping review. *Disabil Rehabil* 2020;**42**:3549-58.
12. Giosa JL, Stolee P, Dupuis SL, Mock SE, Santi SM. Examination of family caregiver experiences during care transitions of older adults. *Can J Aging* 2014;**33**:137-53.
13. Eldridge SM, Chan CL, Campbell MJ, Bond CM, Hopewell S, Thabane L, Lancaster GA; PAFS consensus group. CONSORT 2010 statement: extension to randomised pilot and feasibility trials. *BMJ* 2016;**355**:i5239.
14. Smith T, Clark L, Khoury R, Man MS, Hanson S, Welsh A, Clark A, Hopewell S, Pfeiffer K, Logan P, Crotty M, Costa M, Lamb SE. A feasibility study to assess the design of a multicentre randomized controlled trial of the clinical and cost-effectiveness of a caregiving intervention for people following hip fracture surgery. *Bone Jt Open* 2021;**2**:909-20.
15. Hodkinson KM. Evaluation of a mental test score for assessment of mental impairment in the elderly. *Age Ageing* 1972;**4**:233-8.
16. National Institute for Health and Care Excellence. Hip fracture: management. Clinical guideline CG124 - 2023. Available at: https://www.nice.org.uk/Guidance/CG124 Accessed on: 16 February 2023.
17. Bandura A. Self-efficacy: towards a unifying theory of behavioural change. *Psychol Rev* 1977; **84**:191–215.
18. Saklad M. Grading of patients for surgical procedures. *Anesthesiology* 1941;**2**:281-4.

19. Avery KN, Williamson PR, Gamble C, O'Connell Francischetto E, Metcalfe C, Davidson P, Williams H, Blazeby JM; members of the Internal Pilot Trials Workshop supported by the Hubs for Trials Methodology Research. Informing efficient randomised controlled trials: exploration of challenges in developing progression criteria for internal pilot studies. *BMJ Open* 2017;**7**:e013537.
20. Craig P, Dieppe P, Macintyre S, Michie S, Nazareth I, Petticrew M; Medical Research Council Guidance. Developing and evaluating complex interventions: the new Medical Research Council guidance. *BMJ* 2008;**337**:a1655.
21. Moore GF, Audrey S, Barker M, Bond L, Bonell C, Hardeman W, et al. Process evaluation of complex interventions: Medical Research Council guidance. *BMJ* 2015;**350**:h1258.
22. Skivington K, Matthews L, Simpson SA, Craig P, Baird J, Blazeby JM, Boyd KA, Craig N, French DP, McIntosh E, Petticrew M, Rycroft-Malone J, White M, Moore L. A new framework for developing and evaluating complex interventions: update of Medical Research Council guidance. *BMJ* 2021;**374**:n2061.
23. Gladman JR, Lincoln NB, Adams SA. Use of the extended ADL scale with stroke patients. *Age Ageing* 1993;**22**:419–24.
24. de Rotrou J, Wu YH, Hugonot-Diener L, Thomas-Antérion C, Vidal JS, Plichart M, Rigaud AS, Hanon O. DAD-6: A 6-Item version of the Disability Assessment for Dementia scale which may differentiate Alzheimer's disease and mild cognitive impairment from controls. *Dement Geriatr Cogn Disord* 2012;**33**:210-8.
25. Sekhon M, Cartwright M, Francis JJ. Acceptability of healthcare interventions: an overview of reviews and development of a theoretical framework. *BMC Health Serv Res* 2017;**17**:88.
26. Braun V, Clarke V. Using thematic analysis in psychology. *Qual Res Psychol* 2006;**3**:77-101.
27. Braun V, Clarke V. One size fits all? What counts as quality practice in (reflexive) thematic analysis? *Qual Res Psychol* 2020;**3**:1-25.
28. Engel GL. The need for a new medical model: a challenge for biomedicine. *Science* 1977;**196**:129-36.
29. Treweek S, Miyakoda V, Burke D, Shiely F. Getting it wrong most of the time? Comparing trialists' choice of primary outcome with what patients and health professionals want. *Trials* 2022;**23**:537.
30. Schiller C, Franke T, Belle J, Sims-Gould J, Sale J, Ashe MC. Words of wisdom - patient perspectives to guide recovery for older adults after hip fracture: a qualitative study. *Patient Prefer Adherence* 2015;**9**:57-64.
31. Seitz DP, Adunuri N, Gill SS, Rochon PA. Prevalence of dementia and cognitive impairment among older adults with hip fractures. *J Am Med Direct Assoc* 2011;**12**:556-64.
32. Cooper C, Ketley D, Livingston G. Systematic review and meta-analysis to estimate potential recruitment to dementia intervention studies. *Int J Geriatr Psychiatry* 2014;**29**:515-25.
33. Brambilla M, Parra MA, Della Sala S, Alemanno F, Pomati S. Challenges to recruitment of participants with MCI in a multicentric neuropsychological study. *Aging Clin Exp Res* 2021;**33**:2007-10.
34. Kramer JM, Schwartz A. Reducing barriers to patient-reported outcome measures for people with cognitive impairments. *Arch Phys Med Rehabil* 2017;**98**:1705-15.
35. Elliott J, Forbes D, Chesworth BM, Ceci C, Stolee P. Information sharing with rural family caregivers during care transitions of hip fracture patients. *Int J Integr Care* 2014;**14**:e018.
36. Glenny C, Stolee P, Sheiban L, Jaglal S. Communicating during care transitions for older hip fracture patients: family caregiver and health care provider's perspectives. *Int J Integr Care* 2013;**13**:e044.

For peer review only - http://bmjopen.bmj.com/site/about/guidelines.xhtml

37. Nahm ES, Resnick B, Orwig D, Magaziner J, Degrezia M. Exploration of informal caregiving following hip fracture. *Geriatr Nurs* 2010;**31**:254-62.
38. EuroQol: EQ-5D. Accessed: 29th September 2018. Available at: http://www.euroqol.org/
39. Schwarzer R, Jerusalem M. Generalized Self-Efficacy scale. In Weinman J, Wright S, Johnston M (eds), Measures in health psychology: A user's portfolio. Causal and control beliefs Windsor, UK: NFER-NELSON, 1995; pp. 35-37.
40. Radloff LS. The CES-D scale: A self-report depression scale for research in the general population. *Appl Psycholog Measure* 1977;**1**:385-401.
41. Farrar JT, Young JP Jr, LaMoreaux L, Werth JL, Poole MR. Clinical importance of changes in chronic pain intensity measured on an 11-point numerical pain rating scale. *Pain* 2001;**94**:149-58.
42. Pendergrass A, Beische D, Becker C, Hautinger M, Pfeiffer K. An abbreviated German version of the Sense of Competence Questionnaire among informal caregivers of relatives who had a stroke: development and validation. *Eur J Ageing* 2015;**12**:203-13.
43. Cummings J, Mega M, Gray K, Rosenberg-Thompson S, Carusi DA, Gornbein J. The Neuropsychiatric Inventory: Comprehensive assessment of psychopathology in dementia. *Neurology* 1994;**44**:2308-14.
44. Abbey J, Piller N, De Bellis A, Esterman A, Parker D, Giles L, Lowcay B. The Abbey pain scale: a 1-minute numerical indicator for people with end-stage dementia. *Int J Palliat Nurs* 2004;**10**:6-13.

For peer review only

Table 1: Demographic characteristics of the patient participants at baseline.

	Intervention (N=25)	Control (N=25)
Gender: n (%)		
Male	4 (16.0)	9 (36.0)
Female	21 (84.0)	16 (64.0)
Age in years: mean (SD)	81.4 (8.1)	77.6 (8.6)
Ethnicity: n (%)		
White British	22 (88.0)	23 (92.0)
White Irish	1 (4.0)	1 (4.0)
Indian	0	1 (4.0)
Bangladeshi	2 (8.0)	0
Height (cm): mean (SD)	164.2 (10.6)	166.81 (10.0)
Weight (kg): mean (SD)	65.1 (13.9)	72.6 (17.2)
BMI: mean (SD)	24.1 (4.5)	26.1 (6.2)
AMTS score: median (IQR)	10 (9, 10)	10 (9, 10)
AMTS category		
Cognitive Impairment (score<8)	5 (20.0)	5 (20.0)
No Cognitive Impairment (score≥8)	20 (80.0)	20 (80.0)
Side of hip fracture: n (%)		
Left	10 (40.0)	15 (60.0)
Right	15 (60.0)	10 (40.0)
Hip Fracture classification: n (%)		
Intra-capsular	14 (56.0)	17 (68.0)
Intra-trochanteric	8 (32.0)	5 (20.0)
Sub-trochanteric	3 (12.0)	3 (12.0)
Operative Procedure: n (%)		
Hemiarthroplasty	10 (41.7)	15 (60.0)
THR	2 (8.3)	1 (4.0)
Cannulated screws	3 (12.5)	1 (4.0)
DHS	4 (16.7)	5 (20.0)
Intramedullary device	5 (20.8)	3 (12.0)
Missing	1	0
Length of hospital stay (days): median (IQR)	15 (10, 19)	11 (8, 17)
ASA Grade: median (IQR)	3 (3, 3)	3 (2, 3)
Missing	1	1
Current Medical Diagnoses: n (%)		
Cardiac	8 (32.0)	5 (20.0)
Asthma	2 (8.0)	1 (4.0)
COPD	6 (24.0)	7 (28.0)
Hypertension	12 (48.0)	14 (56.0)
Diabetes	2 (8.0)	5 (20.0)
Stroke	0	2 (8.0)
Cancer	7 (28.0)	3 (12.0)
Osteoarthritis	5 (20.0)	3 (12.0)
Low back pain	4 (16.0)	3 (12.0)
Depression	0	0
Anxiety	0	0
Dementia	2 (8.0)	2 (8.0)
Other	12 (48.0)	11 (44.0)

AMTS – Abbreviated Mental Test Score; ASA - American Society of Anesthesiologists OPD – chronic obstructive pulmonary disease; BMI – body mass index; cm – centimetres; DHS – dynamic hip screw; IQR – inter-quartile range; kg – kilograms; SD – standard deviation; THR – total hip replacement

For peer review only

Table 2: Demographic characteristics of the caregiver participants at baseline.

	Intervention (N=25)	Control (N=25)
Gender: n (%)		
Male	14 (56.0)	9 (37.5)
Female	11 (44.0)	15 (62.5)
Missing	0	1
Age in years: mean (SD)	66.2 (13.6)	57.7 (12.9)
Missing	1	2
Ethnicity: n (%)		
White British	20 (80.0)	23 (95.8)
White Irish	1 (4.0)	0
White Other	1 4.0)	1 (4.2)
Mixed - Other	1 (4.0)	0
Bangladeshi	2 (8.0)	0
Missing	0	1
AMTS score: median (IQR)	10 (10, 10)	10 (10, 10)
Missing	0	1
Relationship to participant: n (%)		
Spouse	10 (40.0)	8 (33.3)
Daughter/Son	13 (52.0)	13 (54.2)
Grandchild	0	1 (4.2)
Other	2 (8.0)	2 (8.3)
Missing	0	1
Caregiver living with participant: n (%)		
Yes	16 (69.6%)	14 (60.9%)
No	7 (30.4%)	9 (39.1%)
Missing	2	2
Occupation: n (%)		
Not working	17 (68.0)	15 (62.5)
Part-time	3 (12.0)	4 (16.7)
Full-time	5 (20.0)	5 (20.8)
Missing	0	1
Current Medical Diagnoses: n (%)		
Cardiac	2 (8.0)	1 (4.0)
Asthma	0	4 (16.0)
COPD	0	0
Hypertension	2 (8.0)	1 (4.0)
Diabetes	1 (4.0)	2 (8.0)
Stroke	0	0
Cancer	1 (4.0)	0
Osteoarthritis	3 (12.0)	1 (4.0)
Low back pain	3 (12.0)	4 (16.0)
Depression	0	3 (12.0)
Anxiety	0	6 (24.0)
Other	3 (12.0)	2 (8.0)

AMTS – Abbreviated Mental Test Score; COPD – chronic obstructive pulmonary disease; SD – standard deviation

For peer review only

Table 3: Progression criteria traffic-light summary table.

	Green (Go)	Amber (Amend)	Red (Stop)	Judgement
**Recruitment**	> 40% of patients screened would be eligible	30% to 40% would be eligible	< 30% would be eligible	39% participants were eligible
**Randomisation acceptability**	> 40% of eligible patients consent to be randomised	20% to 40% would be randomised	< 20% would be randomised	11% of eligible participants were randomised
**Intervention fidelity (healthcare professionals)**	> 70% of participants compliant with their allocated intervention as randomised	50% to 70% received intervention as randomised	< 50% received intervention as randomised	48% received 'complete intervention' as randomised, limited by COVID-19
**Intervention fidelity (caregivers)**	> 90% of participants adopted HIP HELPER intervention post-discharge	60% to 90% adopted HIP HELPER post-discharge	< 60% adopted HIP HELPER post-discharge	Unable to assess with insufficient caregiver logs
**Contamination**	< 5% of participants in either group received majority of their allocated treatment crossover	5% to 10% of participants crossover	> 10% of participants crossover	0% evidence of contamination

For peer review only

Table 4: Table illustrating the frequency to-which the components of the HIP HELPER intervention were delivered to participants.

Item	Intervention (N=25)			
	Session 1 N (%)	Session 2 N (%)	Session 3 N (%)	At least one occurrence during Sessions 1-3
Practical skills-transfers:				
Bed to chair	16 (66.7)	13 (68.4)	8 (57.1)	20 (83.3)
Toilet	4 (16.7)	7 (36.8)	4 (28.6)	12 (50.0)
Walking and walking aids	15 (62.5)	12 (63.2)	9 (64.3)	19 (79.2)
In/out bed	16 (66.7)	12 (63.2)	8 (57.1)	20 (83.3)
Car	3 (12.5)	6 (31.6)	2 (14.3)	9 (37.5)
Stairs	3 (12.5)	6 (31.6)	3 (21.4)	9 (37.5)
Goal Setting theory	21 (87.5)	13 (68.4)	4 (28.6)	22 (91.7)
Goal setting practice	15 (62.5)	15 (79.0)	6 (42.9)	22 (91.7)
Pacing and behaviour theory	20 (83.3)	9 (47.4)	6 (42.9)	22 (91.7)
Pacing and behaviour task	13 (54.2)	10 (52.6)	5 (35.7)	18 (75.0)
Expectations of recovery pathways	22 (91.7)	9 (47.4)	5 (35.7)	23 (95.8)
Practical skills:				
Washing	1 (4.2)	7 (36.8)	1 (7.1)	9 (37.5)
Dressing	1 (4.2)	9 (47.4)	1 (7.1)	9 (37.5)
Caregiver:				
Management discussion	3 (12.5)	7 (36.8)	14 (100)	18 (75.0)
Pacing discussion	4 (16.7)	6 (31.6)	10 (71.4)	16 (66.7)
Case scenario discussion	1 (4.2)	3 (15.8)	9 (64.3)	12 (50.0)
Provision and discussion on HIP HELPER manual	15 (62.5)	4 (21.1)	10 (71.4)	22 (91.7)
Confirmation of HIP HELPER telephone calls	0	0	13 (92.9)	13 (54.2)
Other 1: (“pain relief”, “lack of hip precautions”, “caregiver questions”, “equipment ordering”)	1 (4.2)	2 (10.5)	1 (7.1)	2 (8.3)
Other 2: (“equipment ordering”)	0	0	1 (7.1)	1 (4.2)
Other 3: (“sleep”)	0	0	1 (7.1)	1 (4.2)

Note: Number of planned sessions not performed were: one Session 1, six Session 2, 11 Session 3; Missing data not given, percentages are out of non-missing data.

For peer review only

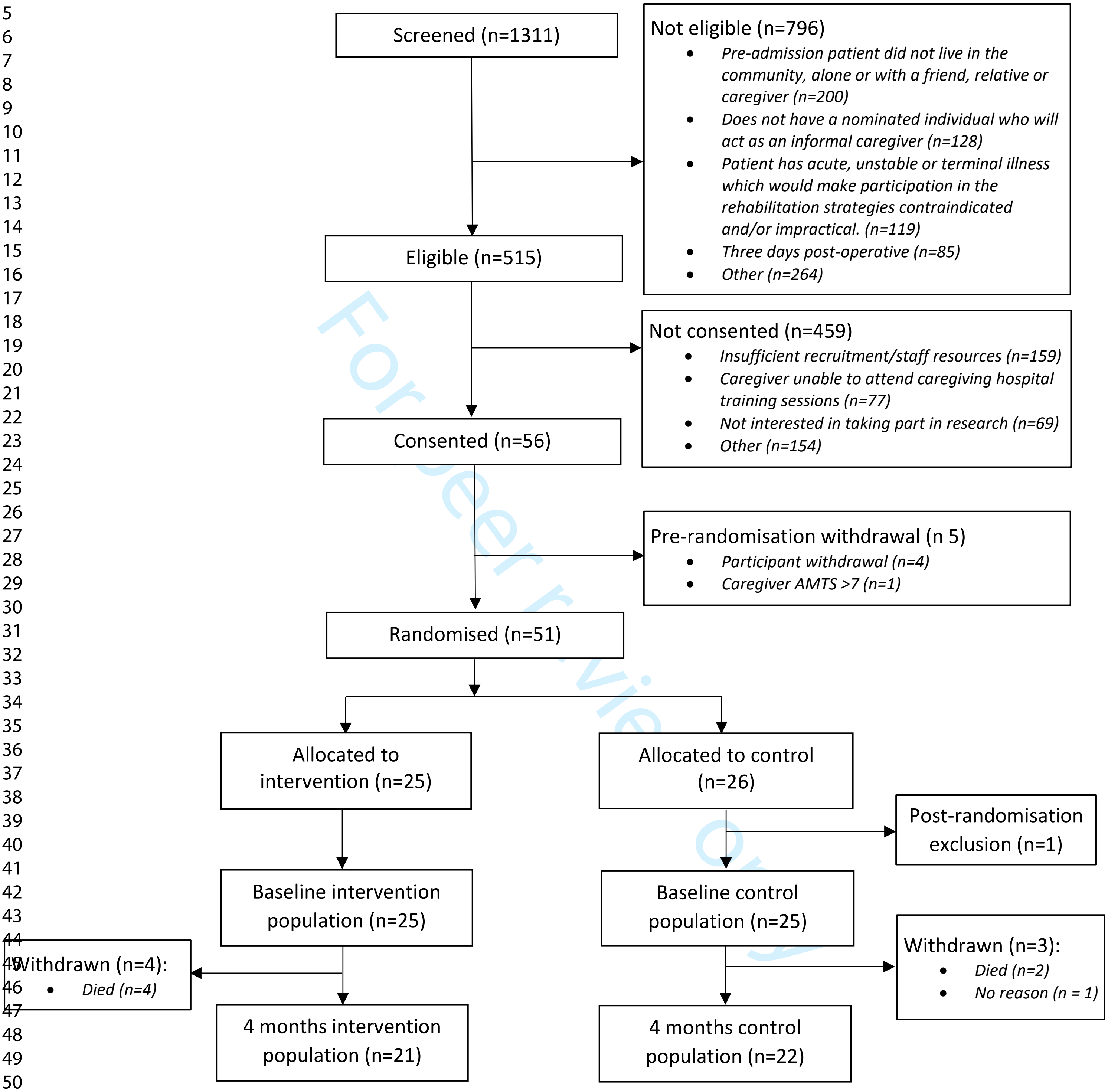

For peer review only

Supplementary File 1: Clinical outcome data collected for patient-participants and caregiver-participants at baseline and four-months post-randomisation.

Patients without cognitive impairment:
- EQ-5D-5L[37]
- Nottingham Activities of Daily Living Scale (NEADL)[23]
- General Self-Efficacy questionnaire[38]
- Center for Epidemiologic Studies Depression Scale (CES-D)[39]
- Numerical rating scale (NRS) for pain (hip and whole body)[40]
- Complications and adverse events including mortality

For all caregivers:
- EQ-5D-5L[37]
- CES-D[39]
- Short Sense of Competence Questionnaire for caregiver burden (SCQ-16)[41]
- Resource Utilization in Dementia questionnaire[9]
- Complications and adverse events including mortality
- Patient and caregiver residential status

PLUS for caregivers of patients with cognitive impairment
- EQ-5D-5L proxy[37]
- Disability Assessment for Dementia Scale-6 (DADS-6) functional score[24]
- Neuropsychiatry Inventory (NPI)[42]
- Abbey Pain Scale[43]

For peer review only - http://bmjopen.bmj.com/site/about/guidelines.xhtml

Supplementary File 2: Reasons for ineligibility in the HIP HELPER study across the five sites.

Site 1	Site 2	Site 3	Site 4	Site 5	Total	Screening	
37	38	11	83	31	200	Pre-admission patient did not live in the community, alone or with a friend, relative or caregiver.	Ineligible Reason
51	22	2	16	37	128	Does not have a nominated individual who will act as an informal caregiver.	
45	12	7	22	33	119	Patient has acute, unstable or terminal illness which would make participation in the rehabilitation strategies contraindicated and/or impractical.	
-	-	-	85	-	85	3+ days post op	
12	1	5	9	27	54	Patients expected by the clinical team to be discharged to a care home (residential or nursing) after their hospital admission	
15	1	1	17	9	43	Patient under age of 65 years	
7	4	-	29	1	41	Patient not willing or able to provide consent or assent depending on the level of cognitive impairment.	
8	1	6	15	8	38	Other	
8	4	-	21	-	33	Caregiver not willing or able to provide consent	
3	2	-	12	2	19	Not undergone hip fracture surgery	
-	-	-	11	2	13	Missing reason	
-	1	-	4	4	9	Participant has significant difficulties reading and/or comprehending English	
-	-	-	2	7	9	Patient under age of 65 years	
-	1	-	-	2	3	Individual caregivers unable to understand written English or have access to a translator.	
1	-	1	-	-	2	Principal (main) caregiver has AMTS score of less than 8	
					**796**	**Total ineligible**	

For peer review only

Supplementary File 3: Reasons for eligible participants not consenting to the HIP HELPER study across the five sites.

Site 1	Site 2	Site 3	Site 4	Site 5	Total	Not Consented	Group
47	26	15	43	27	158	Insufficient recruitment staff resources	Eligible but not consented
10	25	2	16	23	76	Caregiver unable to attend caregiving hospital training sessions	
15	11	6	27	11	70	Not interested in taking part in research	
6	9	1	25	14	55	Other	
21	-	3	13	1	38	Leaving the area	
3	13	1	7	4	28	Missing Reason	
19	-	-	-	1	19	Outlier ward	
-	-	-	-	-	4	Does NOT want to be randomised to receive caregiver intervention	
-	3	1	-	4	4	Persistent post-operative confusion	
-	1	1	-	-	2	Does not have a nominated individual who will act as an informal caregiver.	
-	-	-	-	-	2	Does NOT want to be randomised to receive control (usual care)	
-	-	-	1	2	2	Caregiver not willing or able to provide consent	
-	1	-	-	1	1	Participant has significant difficulties reading and/or comprehending English	
					**459**	**Total not consented**	
-	-	-	-	-	-	Post-operative complication	Not Randomised
	-	-	-	-	1	Persistent post-operative confusion	
-	-	-	-	-	-	Participant no longer wants to take part in research	
-	-	-	-	-	-	Unable to approach caregiver	
-	-	-	-	-	-	Insufficient recruitment staff resources	
2	1	-	-	1	4	Withdrawn	
					**5**	**Total not randomised**	

Supplementary File 4: Recruitment rate presented by each of the five HIP HELPER sites.

Site	Date Opened	Number of Recruited Participants											Total
		April 2021	May 2021	June 2021	July 2021	August 2021	Sept 2021	Oct 2021	Nov 2021	Dec 2021	Jan 2022	Feb 2022	
**Site 1**	07Apr2021	1	3	0	1	1	1	3	0	0			10
**Site 2**	19Apr2021		1	2	2	0	1	2	0	0	0	0	8
**Site 3**	15Jun2021				1	0	0	0	1	1	0	2	5
**Site 4**	29Jun2021				3	1	2	2	0	2	2	1	13
**Site 5**	05Aug2021					0	3	3	4	2	1	2	15
**TOTAL**		**1**	**4**	**2**	**7**	**2**	**7**	**10**	**5**	**5**	**3**	**5**	**51**

For peer review only

Supplementary File 5: Table illustrating the number of participant-dyads that attended intervention sessions.

	Hospital-Based Session			Telephone Booster Calls		
	Session 1 N (%)	Session 2 N (%)	Session 3 N (%)	Telephone 1 N (%)	Telephone 2 N (%)	Telephone 3 N (%)
Yes	24 (96.0)	19 (76.0)	14 (56.0)	13 (54.2)	14 (58.3)	11 (47.8)
No	1 (4.0)	6 (24.0)	11 (44.0)	11 (45.8)	10 (41.7)	12 (52.2)
Missing	0	0	0	1	1	2

For peer review only

Supplementary File 6: Intervention fidelity by site (shown below are those that achieved intervention fidelity).

	Site Number					All Sites
	**1**	**2**	**3**	**4**	**5**	
Intervention fidelity n (%)	2 (40.0)	2 (50.0)	0	3 (50.0)	5 (62.5)	12 (48.0)

N.B. fidelity = 3 in-patient sessions and >= 1 telephone calls

For peer review only

Supplementary File 7: Table illustrating the data completion of clinical outcome scores (baseline and 4-month follow-up).

	Baseline (N; %)		4-Month Follow-up (N; %)	
	**Intervention**	**Control**	**Intervention**	**Control**
Patient participants without Cognitive Impairment (n=41)				
EQ-5D-5L Index	20 (100)	19 (95.0)	14 (70.0)	11 (57.9)
EQ-5D-5L VAS	19 (95.0)	18 (90.0)	14 (70.0)	12 (63.2)
NEADL	15 (75.0)	17 (85.0)	5 (27.8)	9 (52.9)
GSE	19 (95.0)	19 (95.0)	11 (61.1)	10 (58.8)
CES-D	17 (85.0)	16 (80.0)	11 (61.1)	9 (52.9)
NRS pain – hip	20 (100)	18 (90.0)	12 (66.7)	10 (58.8)
NRS pain - body	19 (95.0)	19 (95.0)	12 (66.7)	10 (58.8)
Patient participants with Cognitive Impairment (n=10)				
EQ5D Proxy Index	5 (100)	5 (100)	2 (40.0)	4 (80.0)
EQ5D Proxy VAS	5 (100)	5 (100)	2 (40.0)	4 (80.0)
DADS-6 total (carer)	3 (60.0)	5 (100)	0	4 (80.0)
DADS-6 initiation (carer)	4 (80.0)	5 (100)	0	4 (80.0)
DADS-6 planification (carer)	3 (60.0)	5 (100)	0	4 (80.0)
DADS-6 performance (carer)	4 (80.0)	5 (100)	0	4 (80.0)
NPI severity (carer)	5 (100)	5 (100)	0	3 (60.0)
NPI distress (carer)	5 (100)	5 (100)	0	3 (60.0)
Abbey Pain Scale (carer)	2 (40.0)	5 (100)	0	3 (60.0)
NRS pain – hip	4 (80.0)	4 (80.0)	0	4 (80.0)
NRS pain - body	4 (80.0)	4 (80.0)	0	4 (80.0)
Caregiver participants (n=51)				
EQ-5D-5L Index	24 (96.0)	23 (92.0)	10 (47.6)	11 (50.0)
EQ-5D-5L VAS	24 (96.0)	23 (92.0)	12 (57.1)	12 (54.6)
CESD	20 (80.0)	20 (80.0)	12 (57.1)	9 (40.9)
SCQ total	23 (92.0)	20 (80.0)	12 (57.1)	11 (50.0)
SCQ recipient satisfaction	24 (96.0)	22 (88.0)	12 (57.1)	12 (54.6)
SCQ own satisfaction	23 (92.0)	21 (84.0)	12 (57.1)	12 (54.6)
SCQ consequence	24 (96.0)	23 (92.0	12 (57.1)	11 (50.0)
Patient living in own home: n (%)	24 (96.0)	23 (92.0)	11 (52.4)	11 (50.0)
Caregiver living with participant: n (%)	23 (92.0)	23 (92.0)	12 (57.1)	12 (54.5)

CESD - Center for Epidemiologic Studies Depression Scale; DAD-6 – Disability Assessment for Dementia scale – 6 item; GSE – Generalized Self-Efficacy Scale; NA – not assessed; NEADL - Nottingham Extended Activities of Daily Living scale (NEADL); NPI - Neuropsychiatry Inventory; NRS – numerical rating scale; SCQ – Short Sense of Competence questionnaire for caregiver burden; VAS – visual analogue scale
NB: Deaths prior to 4 month follow-up were assigned zero response accounting for 40% response rate for 2 participants in the patient participants with cognitive impairment responses.

For peer review only

Supplementary File 8: Carer Resource Utilisation in Dementia questionnaire completion statistics at baseline and 4-months

C-RUD Question	Conditional on previous question responses	Baseline		Conditional on previous question responses	4-Months	
		Intervention N=25 n (%)	Control N=25 n (%)		Intervention N=21 n (%)	Control N=22 n (%)
1. Age	No	24 (96.0)	23 (92.0)	No	12 (57.1)	12 (54.6)
2. Gender	No	24 (96.0)	23 (92.0)	No	12 (57.1)	12 (54.6)
3. Relationship to patient	No	23 (92.0)	23 (92.0)	No	12 (57.1)	12 (54.6)
4. Children living with you	No	24 (96.0)	23 (92.0)	No	12 (57.1)	12 (54.6)
5. Do you live with the patient	No	23 (92.0)	23 (92.0)	No	12 (57.1)	12 (54.6)
6. Other caregivers involved	No	23 (92.0)	23 (92.0)	No	12 (57.1)	12 (54.6)
7. Your caring contribution level	No	23 (92.0)	23 (92.0)	No	12 (57.1)	12 (54.6)
8. Sleep in last 30 days	No	24 (96.0)	21 (84.0)	No	12 (57.1)	12 (54.6)
9a. Hours per day assisting patient with tasks such as dressing in last 30 days	No	23 (92.0)	20 (80.0)	No	10 (47.6)	11 (50.0)
9b. Days assisting patient with tasks such as dressing in last 30 days	No	23 (92.0)	20 (80.0)	No	10 (47.6)	11 (50.0)
10a. Hours per day assisting patient with tasks such as shopping in last 30 days	No	23 (92.0)	20 (80.0)	No	12 (57.1)	11 (50.0)
10b. Days assisting patient with tasks such as shopping in last 30 days	No	22 (88.0)	21 (84.0)	No	12 (57.1)	11 (50.0)
11a. Hours per day supervising patient in last 30 days	No	21 (84.0)	21 (84.0)	No	11 (52.4)	12 (54.6)
11b. Days supervising patient in last 30 days	No	21 (84.0)	21 (84.0)	No	11 (52.4)	12 (54.6)
12.Work for pay	No	24 (96.0)	23 (92.0)	No	12 (57.1)	11 (50.0)
13. Stop/reduce working	Yes (N=30)	17 (100)	13 (100)	Yes (N=7)	2 (100)	5 (100)
14. Change of job/working situation	Not Assessed in Baseline C-RUD			Yes (N=7)	2 (100)	5 (100)
15. Hours of paid work per week	Yes (N=17)	7 (100)	10 (100)	Yes (N=1)	1 (100)	0
16. Hours of patient care paid per week	Yes (N=17)	7 (100)	10 (100)	Yes (N=1)	1 (100)	0
17. Hours cut for carer responsibilities in last 30 days	Yes (N=17)	7 (100)	10 (100)	Yes (N=1)	1 (100)	0
18a. Work days missed	No	6 (24.0)	11 (44.0)	Yes (N=7)	2 (100)	3 (60.0)
18b. Part work days missed	No	7 (28.0)	11 (44.0)	Yes (N=7)	2 (100)	5 (100)
19. Stop/reduce working	Not Assessed in Baseline C-RUD			Yes (N=1)	1 (100)	0

For peer review only

20. Admitted to hospital in last 30 days	No	24 (96.0)	23 (92.0)	Yes (N=23)	12 (100)	10 (90.9)
21. Nights in each ward in last 30 days	Yes (N=50)	23 (92.0)	23 (92.0)	Yes (N=23)	2 (16.7)	1 (9.1)
22. Hospital ER care in last 30 days	No	23 (92.0)	23 (92.0)	Yes (N=23)	12 (100)	9 (81.8)
23. Health care professional visits in last 30 days	No	24 (96.0)	22 (88.0)	Yes (N=23)	10 (83.3)	10 (90.9)
24. Current medications	No	22 (88.0)	18 (72.0)	No	8 (38.1)	8 (36.4)
25. Patient change of living accommodation since last visit	Not Assessed in Baseline C-RUD			No	12 (57.1)	11 (50.0)
26. Patient current living accommodation	No	24 (96.0)	23 (92.0)	Yes (N=0)	0	0
27. Date living change occurred	Not Assessed in Baseline C-RUD			Yes (N=0)	0	0
28. Reason for living change	Not Assessed in Baseline C-RUD			Yes (N=0)	0	0
29. Who patient lives with	No	24 (96.0)	23 (92.0)	Not Assessed in Follow-Up C-RUD		
30. Patient temporary accommodation in last 30 days	No	7 (28.0)	6 (24.0)	Yes (N=23)	8 (38.1)	5 (45.5)
31. Patient admitted to hospital in last 30 days	No	23 (92.0)	21 (84.0)	No	10 (47.6)	12 (54.6)
32. Patient nights in each ward in last 30 days	No	19 (76.0)	16 (64.0)	No	4 (19.1)	1 (4.6)
33. Patient hospital ER care in last 30 days	No	24 (96.0)	21 (84.0)	No	11 (52.4)	11 (50.0)
34. Patient health care professional visits in last 30 days	No	21 (84.0)	20 (80.0)	No	7 (33.3)	9 (40.9)
35. Patient service visits in last 30 days	No	22 (88.0)	21 (84.0)	No	8 (38.1)	11 (50.0)

* Questions are deemed completed if the main parts of the question have all been completed (e.g., checkboxes), and completion rates for some questions are conditional on previous responses.

For peer review only

Supplementary File 9: Table illustrating descriptive clinical outcomes presented as median and inter-quartile ranges (baseline and 4-month follow-up).

	Baseline		4-Month Follow-Up	
	**Intervention**	**Control**	**Intervention**	**Control**
Patient participants without Cognitive Impairment				
EQ-5D-5L Index	-0.10 (-0.26, 0.23)	0.05 (-0.15, 0.41)	0.60 (0.22, 0.77)	0.60 (0.34, 0.67)
EQ-5D-5L VAS	50.0 (30.0, 55.0)	47.5 (15.0, 50.0)	55.0 (30.0, 80.0)	52.5 (35.0, 80.0)
NEADL	13 (12, 20)	17(14, 21)	20 (18, 20)	16 (13, 20)
GSE	32 (26, 36)	34 (30, 40)	29.91 (5.87)	30.70 (4.83)
CES-D	23.0 (17.0, 27.0)	22.0 (18.0, 29.0)	14 (13, 18)	19 (16, 20)
NRS pain – hip	90.0 (73.5, 100.0)	82.5 (54.0, 97.0)	20.0 (4.0, 35.0)	14.5 (10.0, 45.0)
NRS pain - body	60.0 (20.0, 90.0)	55.0 (35.0, 70.0)	17.0 (1.5, 35.0)	32.5 (14.0, 64.0)
Patient participants with Cognitive Impairment				
EQ5D Proxy Index	0.15 (-0.20, 0.34)	0.22 (0.01, 0.50)	0 (0, 0)	0.33 (0.07, 0.50)
EQ5D Proxy VAS	50.00 (15.0, 50.0)	60.0 (30.0, 75.0)	0 (0, 0)	57.5 (37.5, 67.5)
DADS-6 total (carer)	2.0 (1.0, 4.00)	1.0 (1.0, 4.0)	NA	0.0 (0.0, 7.5)
DADS-6 initiation (carer)	1.5 (0.5, 3.0)	0.0 (0.0, 1.00)	NA	0.0 (0.0, 2.5)
DADS-6 planification (carer)	0.0 (0.0, 1.0)	1.0 (0.0, 2.0)	NA	0.0 (0.0, 2.5)
DADS-6 performance (carer)	1.5 (0.5, 2.5)	1.0 (0.0, 1.0)	NA	0.0 (0.0, 2.5)
NPI severity (carer)	10 (5, 12)	3 (3, 7)	NA	5 (1, 11)
NPI distress (carer)	9 (1, 11)	1 (0, 2)	NA	1 (1, 11)
Abbey Pain Scale (carer)	6.5 (3.0, 10.0)	5.0 (5.0, 6.0)	NA	5 (4, 5)
NRS pain – hip	100.0 (70.0, 100.0)	60.0(45.0, 75.0)	NA	20 (5, 50)
NRS pain - body	35.0 (20.0, 60.0)	45.0 (20.5, 65.0)	NA	51 (26, 70)
Caregiver participants				
EQ-5D-5L Index	0.80(0.71, 1.00)	1.00 (0.77, 1.00)	0.88 (0.71, 1.00)	0.77 (0.68, 1.00)
EQ-5D-5L VAS	85.0 (80.0, 92.5)	85.0 (80.0, 95.0)	82.5 (72.5, 92.5)	77.5 (67.5, 90.0)
CESD	14.0 (12.0, 19.0)	13.5 (11.5, 19.5)	16.5 (13.5, 18.0)	13.0 (12.0, 19.0)
SCQ total	60.0 (53.0, 65.0)	63.5 (60.0, 68.0)	65.5 (58.0, 74.5)	60.0(55.0, 68.0)
SCQ recipient satisfaction	17.5 (15.5, 20.0)	18.0 (16.0, 20.0)	20.0 (17.0, 20.0)	20.0 (16.0, 20.0)
SCQ own satisfaction	19.0 (17.0, 21.0)	21.0 (18.0, 22.0)	21.0 (19.5, 23.5)	18.0 (17.5, 20.5)
SCQ consequence	26.0 (21.5, 27.0)	27.0 (22.0, 28.0)	27.0 (17.5, 32.0)	24.0 (21.0, 26.0)
Patient living in own home: n=Yes (%)	23 (95.8)	21 (91.3)	11 (100.0)	10 (100.0)
Caregiver living with participant: n=Yes (%)	16 (69.6)	14 (60.9)	11 (91.7)	7 (58.3)

CESD - Center for Epidemiologic Studies Depression Scale; DAD-6 – Disability Assessment for Dementia scale – 6 item; GSE – Generalized Self-Efficacy Scale; NA – not assessed; NEADL - Nottingham Extended Activities of Daily Living scale (NEADL); NPI - Neuropsychiatry Inventory; NRS – numerical rating scale; SCQ – Short Sense of Competence questionnaire for caregiver burden; VAS – visual analogue scale

For peer review only

Supplementary File 10: Acceptability questionnaire (participant) by question number.

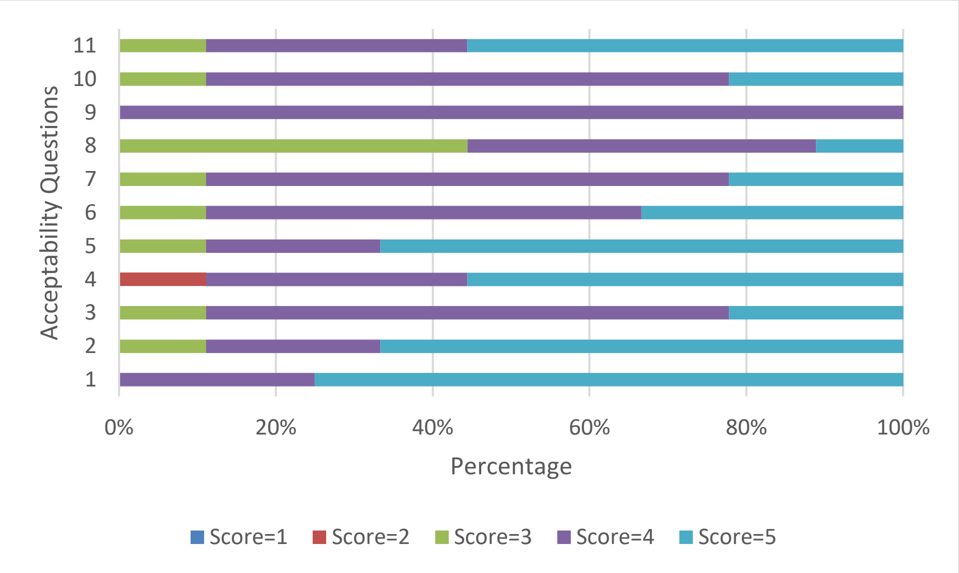

Question 1: How acceptable was the HIP HELPER in hospital training (Missing=13)
Question 2: How acceptable were the 3 HIP HELPER telephone calls? (Missing=12)
Question 3: How acceptable was the HIP HELPER Workbook? (Missing=12)
Question 4: How much effort was it to engage with the HIP HELPER in-hospital training? (Missing=12)
Question 5: How much effort did it take to engage with the HIP HELPER telephone calls? (Missing=12)
Question 6: How much effort did it take to engage with the HIP HELPER workbook? (Missing=12)
Question 7: To what extent does the HIP HELPER programme fit with your belief about recovery after a hip fracture operation? (Missing=12)
Question 8: Is the HIP HELPER programme likely to change your ability to help your friend/family member's recover after a hip fracture operation? (Missing=12)
Question 9: Does the HIP HELPER programme provide you with more confidence on your skills to help someone after a hip fracture operation? (Missing=12)
Question 10: Is it clear how the HIP HELPER intervention could help recovery after a hip fracture operation? (Missing=12)
Question 11: Did doing the HIP HELPER programme interrupt with you other priorities? (Missing=12)

Note: higher scores indicate greater acceptability.

For peer review only

Supplementary File 11: Acceptability questionnaire (caregiver) by question number.

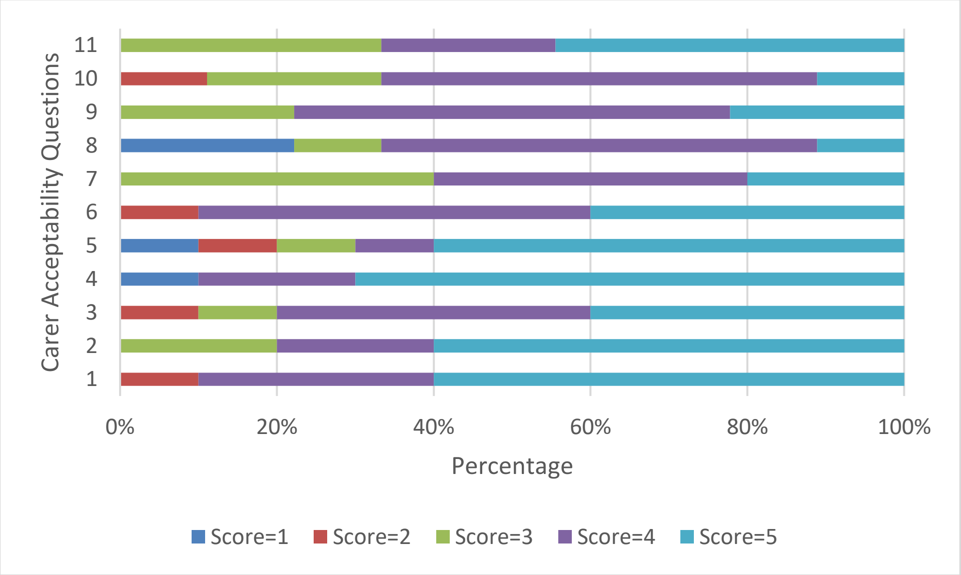

Question 1: How acceptable was the HIP HELPER in hospital training
Question 2: How acceptable were the 3 HIP HELPER telephone calls?
Question 3: How acceptable was the HIP HELPER Workbook?
Question 4: How much effort was it to engage with the HIP HELPER in-hospital training?
Question 5: How much effort did it take to engage with the HIP HELPER telephone calls?
Question 6: How much effort did it take to engage with the HIP HELPER workbook?
Question 7: To what extent does the HIP HELPER programme fit with your belief about recovery after a hip fracture operation?
Question 8: Is the HIP HELPER programme likely to change your ability to help your friend/family member's recover after a hip fracture operation?
Question 9: Does the HIP HELPER programme provide you with more confidence on your skills to help someone after a hip fracture operation?
Question 10: Is it clear how the HIP HELPER intervention could help recovery after a hip fracture operation?
Question 11: Did doing the HIP HELPER programme interrupt with you other priorities?

Note: higher scores indicate greater acceptability.

For peer review only

Supplementary File 12: Summary of safety outcomes at the end of the study for all participants.

Adverse Event	Frequency
Death	7
Falls	5
Joint infection	4
Increased pain (hip)	4
Increased pain (other joints)	4
Anxiety/depression	4
Atrial fibrillation	1
Deep wound infection	1
Wound infection	1
Skin integrity complication	1
PE	1
Stroke	1
Bowel obstruction	1
Barrett's oesophagus	1
**Total**	**36**

For peer review only

Supplementary File 13: Characteristics of qualitative investigation sample.

Person with hip fracture	Intervention	Control
N	7	3
Mean age (years)	77.85	69.33
Gender (M/F)	6 / 1	0 / 3
Ethnicity		
White British	7	3
Site (n)		
1	1	1
2	1	1
3	1	1
4	3	-
5	1	-
**Healthcare Professionals**	**Frequency**	
**Professional Role**		
Physiotherapist	4	
Occupational therapist	2	
Nurse	1	
Researcher	1	
**Site**		
1	2	
2	2	
3	1	
4	2	
5	1	

For peer review only

CONSORT checklist of information to include when reporting a pilot trial*

Section/topic and item No	Standard checklist item	Extension for pilot trials	Page No where item is reported
**Title and abstract**			
1a	Identification as a randomised trial in the title	Identification as a pilot or feasibility randomised trial in the title	Title.
1b	Structured summary of trial design, methods, results, and conclusions (for specific guidance see CONSORT for abstracts)	Structured summary of pilot trial design, methods, results, and conclusions (for specific guidance see CONSORT abstract extension for pilot trials)	Abstract.
**Introduction**			
Background and objectives:			
2a	Scientific background and explanation of rationale	Scientific background and explanation of rationale for future definitive trial, and reasons for randomised pilot trial	Introduction, Para 1-4
2b	Specific objectives or hypotheses	Specific objectives or research questions for pilot trial	Introduction, Para 5.
**Methods**			
Trial design:			
3a	Description of trial design (such as parallel, factorial) including allocation ratio	Description of pilot trial design (such as parallel, factorial) including allocation ratio	Methods, Study Design
3b	Important changes to methods after trial commencement (such as eligibility criteria), with reasons	Important changes to methods after pilot trial commencement (such as eligibility criteria), with reasons	None. Protocol Submitted
Participants:			
4a	Eligibility criteria for participants		
4b	Settings and locations where the data were collected		Methods, Eligibility Criteria
4c		How participants were identified and consented	Methods, Eligibility Criteria.
Interventions:			
5	The interventions for each group with sufficient details to allow replication, including how and when they were actually administered		Methods, Study Treatments.
Outcomes:			
6a	Completely defined prespecified primary and secondary outcome measures, including how and when they were assessed	Completely defined prespecified assessments or measurements to address each pilot trial objective specified in 2b, including how and when they were assessed	Methods, Outcome Measures, Data Collection
6b	Any changes to trial outcomes after the trial commenced, with reasons	Any changes to pilot trial assessments or measurements after the pilot trial commenced, with reasons	None.
6c		If applicable, prespecified criteria used to judge whether, or how, to proceed with future definitive trial	Protocol Submitted.

Sample size:			
7a	How sample size was determined	Rationale for numbers in the pilot trial	Methods, Sample size
7b	When applicable, explanation of any interim analyses and stopping guidelines		
Randomisation:			
Sequence generation:			
8a	Method used to generate the random allocation sequence		Methods, Randomisation + Blinding.
8b	Type of randomisation; details of any restriction (such as blocking and block size)	Type of randomisation(s); details of any restriction (such as blocking and block size)	
Allocation concealment mechanism:			
9	Mechanism used to implement the random allocation sequence (such as sequentially numbered containers), describing any steps taken to conceal the sequence until interventions were assigned		Methods, Randomisation + Blinding
Implementation:			
10	Who generated the random allocation sequence, enrolled participants, and assigned participants to interventions		Methods, Randomisation + Blinding
Blinding:			
11a	If done, who was blinded after assignment to interventions (eg, participants, care providers, those assessing outcomes) and how		Methods, Randomisation + Blinding
11b	If relevant, description of the similarity of interventions		N/A.
Analytical methods:			
12a	Statistical methods used to compare groups for primary and secondary outcomes	Methods used to address each pilot trial objective whether qualitative or quantitative	Methods, Data Analyses + Progression Criteria.
12b	Methods for additional analyses, such as subgroup analyses and adjusted analyses	Not applicable	
**Results**			
Participant flow (a diagram is strongly recommended):			Figure 1.
13a	For each group, the numbers of participants who were randomly assigned, received intended treatment, and were analysed for the primary outcome	For each group, the numbers of participants who were approached and/or assessed for eligibility, randomly assigned, received intended treatment, and were assessed for each objective	Figure 1 Results, Patient Characteristics + Treatments
13b	For each group, losses and exclusions after randomisation, together with reasons		Figure 1.
Recruitment:			

2/4

14a	Dates defining the periods of recruitment and follow-up		N/A.
14b	Why the trial ended or was stopped	Why the pilot trial ended or was stopped	
Baseline data:			
15	A table showing baseline demographic and clinical characteristics for each group		Table 1 + 2.
Numbers analysed:			
16	For each group, number of participants (denominator) included in each analysis and whether the analysis was by original assigned groups	For each objective, number of participants (denominator) included in each analysis. If relevant, these numbers should be by randomised group	Figure 1.
Outcomes and estimation:			
17a	For each primary and secondary outcome, results for each group, and the estimated effect size and its precision (such as 95% confidence interval)	For each objective, results including expressions of uncertainty (such as 95% confidence interval) for any estimates. If relevant, these results should be by randomised group	Supp File 9.
17b	For binary outcomes, presentation of both absolute and relative effect sizes is recommended	Not applicable	Supp File 9.
Ancillary analyses:			
18	Results of any other analyses performed, including subgroup analyses and adjusted analyses, distinguishing prespecified from exploratory	Results of any other analyses performed that could be used to inform the future definitive trial	Supp File 9.
Harms:			
19	All important harms or unintended effects in each group (for specific guidance see CONSORT for harms)		Supp File 10
19a		If relevant, other important unintended consequences	Supp File 10.
**Discussion**			
Limitations:			
20	Trial limitations, addressing sources of potential bias, imprecision, and, if relevant, multiplicity of analyses	Pilot trial limitations, addressing sources of potential bias and remaining uncertainty about feasibility	Discussion, Para 6.
Generalisability:			
21	Generalisability (external validity, applicability) of the trial findings	Generalisability (applicability) of pilot trial methods and findings to future definitive trial and other studies	Discussion, Para 3, 4 + 6.
Interpretation:			
22	Interpretation consistent with results, balancing benefits and harms, and considering other relevant evidence	Interpretation consistent with pilot trial objectives and findings, balancing potential benefits and harms, and considering other relevant evidence	Discussion. Para 1
22a		Implications for progression from pilot to future definitive trial, including any proposed amendments	Conclusion. Table 3.
**Other information**			

3/4

Registration:			
23	Registration number and name of trial registry	Registration number for pilot trial and name of trial registry	Abstract + Acknowledgement + Declaration
Protocol:			
24	Where the full trial protocol can be accessed, if available	Where the pilot trial protocol can be accessed, if available	Ref. 13 + Supp. Upload.
Funding:			
25	Sources of funding and other support (such as supply of drugs), role of funders		Acknowledgement + Declaration
26		Ethical approval or approval by research review committee, confirmed with reference number	Acknowledgement + Declaration

*Here a pilot trial means any randomised study conducted in preparation for a future definitive RCT, where the main objective of the pilot trial is to assess feasibility.

4/4

Hospital-based caregiver intervention for people following hip fracture surgery (HIP HELPER): multi-centre randomised controlled feasibility trial with embedded qualitative study.

Journal:	*BMJ Open*
Manuscript ID	bmjopen-2023-073611.R1
Article Type:	Original research
Date Submitted by the Author:	25-Oct-2023
Complete List of Authors:	Smith, Toby; University of Warwick Warwick Medical School, Warwick Clinical Trials Unit; University of East Anglia, Faculty of Medicine and Health Sciences<br>Khoury, Reema; University of East Anglia, Norwich Medical School<br>Hanson, Sarah; University of East Anglia, School of Health Sciences<br>Welsh, Allie; University of East Anglia, School of Education<br>Grant, Kelly; University of East Anglia, Norwich Medical School<br>Clark, Allan; University of East Anglia, Norwich Medical School<br>Ashford, Polly-Anna; University of East Anglia, Norwich Medical School<br>Hopewell, Sally; University of Oxford, Nuffield Department of Orthopaedics, Rheumatology and Musculoskeletal Sciences<br>Pfeiffer, K; Robert Bosch Hospital, Department of Clinical Gerontology and Geriatric Rehabilitation<br>Logan, Phillipa; University of Nottingham, School of Medicine<br>Crotty, Maria; Flinders University, College of Medicine and Public Health<br>Costa, Matthew; University of Oxford, Nuffield Department of Orthopaedics, Rheumatology, and Musculoskeletal Sciences<br>Lamb, Sarah; University of Exeter, College of Medicine and Health; University of Oxford, NDORMS
**Primary Subject Heading**:	Rehabilitation medicine
Secondary Subject Heading:	Rheumatology
Keywords:	ORTHOPAEDIC & TRAUMA SURGERY, Hip < ORTHOPAEDIC & TRAUMA SURGERY, REHABILITATION MEDICINE, Nursing Care

SCHOLARONE™
Manuscripts

I, the Submitting Author has the right to grant and does grant on behalf of all authors of the Work (as defined in the below author licence), an exclusive licence and/or a non-exclusive licence for contributions from authors who are: i) UK Crown employees; ii) where BMJ has agreed a CC-BY licence shall apply, and/or iii) in accordance with the terms applicable for US Federal Government officers or employees acting as part of their official duties; on a worldwide, perpetual, irrevocable, royalty-free basis to BMJ Publishing Group Ltd ("BMJ") its licensees and where the relevant Journal is co-owned by BMJ to the co-owners of the Journal, to publish the Work in this journal and any other BMJ products and to exploit all rights, as set out in our licence.

The Submitting Author accepts and understands that any supply made under these terms is made by BMJ to the Submitting Author unless you are acting as an employee on behalf of your employer or a postgraduate student of an affiliated institution which is paying any applicable article publishing charge ("APC") for Open Access articles. Where the Submitting Author wishes to make the Work available on an Open Access basis (and intends to pay the relevant APC), the terms of reuse of such Open Access shall be governed by a Creative Commons licence – details of these licences and which Creative Commons licence will apply to this Work are set out in our licence referred to above.

Other than as permitted in any relevant BMJ Author's Self Archiving Policies, I confirm this Work has not been accepted for publication elsewhere, is not being considered for publication elsewhere and does not duplicate material already published. I confirm all authors consent to publication of this Work and authorise the granting of this licence.

For peer review only

BMJ OPEN

TITLE PAGE

Title: Hospital-based caregiver intervention for people following hip fracture surgery (HIP HELPER): multi-centre randomised controlled feasibility trial with embedded qualitative study.

Authors: Smith TO,[1,2] Khoury R,[2] Hanson S,[2] Welsh A,[2] Grant K,[2] Clark A,[2] Ashford P-A,[2] Hopewell S,[3] Pfeiffer K,[4] Logan PA,[5] Crotty M,[6] Costa ML,[3] Lamb SE,[7] on behalf of the HIP HELPER Study Collaborators.

Affiliations

1. Warwick Clinical Trials Unit, Warwick Medical School, University of Warwick, Coventry, UK
2. Faculty of Medicine and Health Sciences, University of East Anglia, Norwich, UK
3. Oxford Trauma and Emergency Care, Nuffield Department of Rheumatology, Orthopaedics and Musculoskeletal Sciences, University of Oxford, Oxford, UK
4. Department of Geriatric Rehabilitation, Robert-Bosch-Hospital, Stuttgart, Germany
5. School of Medicine, University of Nottingham, Nottingham, UK
6. College of Medicine and Public Health, Flinders University, Adelaide, Australia
7. Exeter Medical School, University of Exeter, Exeter, UK

Corresponding Author: Professor Toby Smith, Warwick Medical School, University of Warwick, Coventry, CV1 4AL. Email: toby.smith@uea.ac.uk

For peer review only

ABSTRACT

Objectives: To assess the feasibility of conducting a pragmatic, multi-centre randomised controlled trial (RCT) to test the clinical and cost-effectiveness of an informal caregiver training programme to support the recovery of people following hip fracture surgery.

Design: Two-arm, multi-centre, pragmatic, open, feasibility RCT with embedded qualitative study.

Setting: National Health Service (NHS) providers in five English hospitals.

Participants: Community-dwelling adults, aged 60 years and over, who undergo hip fracture surgery and their informal caregivers.

Intervention: Usual care: usual NHS care. Experimental: usual NHS care *plus* a caregiver-patient dyad training programme (HIP HELPER). This programme comprised of three, one-hour, one-to-one training sessions for a patient and caregiver, delivered by a nurse, physiotherapist or occupational therapist in the hospital setting pre-discharge. After discharge, patients and caregivers were supported through three telephone coaching sessions.

Randomisation and blinding: Central randomisation was computer generated (1:1), stratified by hospital and level of patient cognitive impairment. There was no blinding.

Main outcome measures: Data collected at baseline and four months post-randomisation included: screening logs, intervention logs, fidelity checklists, acceptability data and clinical outcomes. Interviews were conducted with a subset of participants and health professionals.

Results: 102 participants were enrolled (51 patients; 51 caregivers). Thirty-nine percent (515/1311) of patients screened were eligible. Eleven percent (56/515) of eligible patients consented to be randomised. Forty-eight percent (12/25) of the intervention group reached compliance to their allocated intervention. There was no evidence of treatment contamination. Qualitative data demonstrated the trial and HIP HELPER programme was acceptable.

Conclusions: The HIP HELPER programme was acceptable to patient-caregiver dyads and health professionals. The COVID-19 pandemic impacting on site's ability to deliver the research. Modifications are necessary to the design for a viable definitive RCT.

Trial registration number: ISRCTN13270387

Data availability statement: The data that support the findings of this study are available from the corresponding author (TS) upon reasonable request. This includes access to the full protocol, anonymised participant-level dataset and statistical code.

STRENGTHS AND LIMITATIONS OF THIS STUDY

- Mixed-method approach provided useful feasibility and acceptability data.
- Assessment of diverse measures allowed evaluation of data collection for key outcome domains.
- Participant experiences and acceptability data suggest perceived value in the HIP HELPER programme.

For peer review only

- 10% of the cohort were living with cognitive impairment; none were recruited to the qualitative sub-study.
- COVID-19 pandemic affected NHS services, which impacted on study delivery.

KEYWORDS: trauma; femoral fracture; recovery; rehabilitation; domiciliary; carer; home network; RCT

WORD COUNT: 4298 manuscript; 280 abstract

For peer review only

INTRODUCTION

Hip fracture is a serious injury for older people [1]. Approximately 80,000 people aged 60 years and over experience a fragility hip fracture in the United Kingdom (UK) annually [2]. This has an estimated combined health and social cost of over £2 billion [3].

People have frequently experienced poor recovery following hip fracture [4]. The majority never return to pre-injury levels of function [3,5]. Health-related quality of life (HRQoL) is reduced and mortality is high [5,6]. Patients also often experience repeated falls. This leads to reduced independence and confidence in self-caring skills. Approximately 20% of patients who previously lived at home move into institutional care following hip fracture [7]. For those who do return home, informal caregivers who support their friend's/family member's care needs frequently experience physical and mental stress [4]. A high caregiver burden that has previously been reported by 50%, 36%, and 26% at 1-month, 3-months, and 1-year post-surgery [8] shows the multifaceted strain perceived by at least a sub-group of hip-fracture caregivers.

People after hip fracture who return home often need help. This ranges from assistance with personal activities of daily living (ADLs) such as toileting, washing, dressing and eating, to more complex tasks such as managing money, shopping and household chores [9]. Most of this required help is provided by family members or friends. Depending on the pre-fracture status of the patient, some of these informal caregivers continue in their caregiving role, others become a first-time caregiver.

Whilst informal caregivers may be willing to support their friend/family member, they frequently feel under-skilled, and have low confidence to do so [10]. A lack of information sharing, disorganised discharge planning and unclear individual roles have been identified as challenges for patients following hip fracture and their caregivers during care transitions [11]. Teaching caregiver skills to better support patients following hip fracture may improve HRQoL and independence, whilst reducing the burden of impairment for patients and caregivers [10,12].

This study aimed to assess the feasibility of conducting a pragmatic, multi-centre, randomised controlled trial (RCT) to test the clinical and cost-effectiveness of an informal caregiver training programme to support the recovery of people following hip fracture surgery.

METHODS

The study was reported to satisfy the CONSORT extension for reporting pilot and feasibility RCTs[13]. A full protocol has been published previously [14]. The study followed the published protocol with the exception of the introduction of the optional delivery of the HIP HELPER programme through an online approach rather than face-to-face delivery. This was in response to the COVID-19 pandemic, and enacted for one participant-dyad.

Study Design

This was a feasibility study comprising of a parallel, multicentre, pragmatic RCT and embedded qualitative study. The study process evaluation results are presented in this paper.

The study flowchart is presented as **Figure 1**.

For peer review only - http://bmjopen.bmj.com/site/about/guidelines.xhtml

Eligibility Criteria

Participants were recruited from orthopaedic services in five National Health Service (NHS) hospitals in England providing hip fracture surgery. We recruited adults who previously lived in the community (not institutional care), aged 60 years and over, who had undergone hip fracture surgery, could nominate an informal caregiver and provided both patient-caregiver consent to participate. Where a patient-participant did not have capacity, agreement from a consultee was sought.

We excluded people who had acute, unstable or terminal illness or were expected by the clinical team to be discharged to a care home (residential or nursing). Caregivers were ineligible if they had an Abbreviated Mental Test Score (AMTS) [15] of less than eight.

Study Treatments

Usual NHS surgical and rehabilitation care was received by both control and intervention groups [16]. Accordingly, post-hip fracture surgery, all participants received pre-discharge care including nursing, physiotherapy, occupational therapy and social service needs-assessment (where appropriate). Patients and their caregivers in the control group, did not receive the HIP HELPER programme, with no additional in-patient or out-patient caregiver training.

The HIP HELPER intervention has been previously described [14]. In brief, this was a patient-caregiver dyad training programme. The theoretical principle behind the programme is a social learning theory [17].

In practice, people randomised to the experimental group received the usual NHS care in addition to the HIP HELPER programme. The only difference between the groups was the addition of three, 60-minute, health professional-caregiver dyad HIP HELPER training sessions, performed in the hospital setting whilst the patient was an in-patient, and three follow-up telephone calls one, three and six weeks after hospital discharge. In the in-patient sessions, participants were taught about the normal recovery process, and skills in goal-setting, pacing, activity behaviour modification and stress management. They were also taught skills on manual handling, transfers, walking and how to support people with ADLs. The follow-up telephone calls aimed to re-enforce the skills developed in the face-to-face sessions, support any set-backs in recovery and to develop longer-term goals.

Each health professional (physiotherapist, occupational therapist or nurse) who delivered the experimental intervention attended a one-day training session, which taught the components and format of the programme. To promote compliance with the treatment protocol, the Central Trial Team had regular contact with clinical team members, reviewing the first HIP HELPER sessions for intervention fidelity and held monthly meetings regarding study processes.

Data Collection

At the time of enrolment, sites checked eligibility and recorded demographic characteristics in the screening log. Baseline assessments were undertaken after consent was obtained, prior to randomisation. Data collected at baseline included: hospital admission, age, sex, ethnicity, height, weight, patient cognitive impairment assessed using the AMTS [15], past medical history, American Society of Anaesthesiologists (ASA) grade [18], side of hip fracture, operative procedure and hip fracture classification. Caregiver demographic data collected included: relationship of caregiver to patient, age, sex, ethnicity, past medical history, AMTS [15], whether they lived with the patient, employment status and experience of being a caregiver (for this patient and/or for another person).

For peer review only

Participants were followed-up at four months post-randomisation. Data were collected via postal questionnaires by the central trial team.

Outcome Measures

To answer our feasibility objectives, we assessed:

1. *Recruitment feasibility* – by screening log data on: number of potential participants and their caregivers assessed for eligibility, including reasons for exclusion/non-participation, and consented to be randomised; timing and location of approach and consent.

2. *Intervention acceptability* – by qualitative interviews with participants and health professionals; acceptability questionnaire, study attrition at the intervention phase.

3. *Intervention fidelity (healthcare professionals)* – by intervention log data on: HIP HELPER session duration, frequency, location (orthopaedic/orthogeriatric ward, rehabilitation ward or other); Quality Assurance (QA) to monitor HIP HELPER programme delivery.

4. *Intervention fidelity (caregivers)* – by caregiver HIP HELPER programme intervention logs; qualitative interviews.

5. *Randomisation acceptability* – by screening logs, eligibility assessment logs and consent forms; participant attrition; qualitative investigation.

6. *Risk of contamination* - by HIP HELPER programme log data including: QA monitoring visit checklists; delegation logs; qualitative interviews with health professionals.

7. *Completeness of outcome measures* - by completion rates (baseline and four months post-randomisation). Outcome measures collected are included in **Supplementary File 1**.

Randomisation and blinding

Randomisation was at the patient-caregiver dyad level (1:1 experimental and control groups) by stratification for: hospital and the presence of patient cognitive impairment (AMTS[15] < or ≥ 8 points). Sites team members performed randomisation post-baseline data collection. Allocation was concealed prior to randomisation. Randomisation was computer generated, performed by site team members on a secure, online programme, centrally administered by an independent programmer at the Norwich CTU (NCTU). The randomisation sequence was generated by NCTU programmers, tested by the trial statistician.

Due to the participatory nature of the intervention, blinding participants or the site team was not possible. Senior research team members were blinded to treatment allocation for the duration of the study.

Sample Size

We aimed to recruit 120 participants (60 patients; 60 caregivers). This was considered sufficient to answer our feasibility objectives and assess the *a priori* progression criteria based on Teare et al [19] recommendations.

Data Analysis and Progression Criteria

For peer review only

Consent rates, recruitment rates, attrition, missing data rates and intervention fidelity were reported as proportions with 95% confidence intervals (CIs) presented for consent and recruitment rates. The analysis of clinical outcome measures was descriptive, reported as means and standard deviations (SD) or medians and interquartile ranges (IQR) and numbers and percentages for binary and categorical variables. No formal statistical testing was undertaken.

A 'traffic light' system was used as a guide for progression to a definitive trial [20]. The progression criteria were centred around recruitment, retention, intervention fidelity and contamination.

Study Monitoring

A Trial Oversight Committee (TOC) was appointed to independently review data on safety, protocol adherence and study processes.

Patient and Public Involvement

Patient involvement began during protocol development and continued throughout the study. One patient-member (not enrolled in the study) attended TOC meetings. They provided insights into the study conduct, particularly on data collection processes and helped interpret the findings to inform the study's dissemination phase.

Participants who expressed an interest in receiving information on the findings were provided with this.

Embedded Qualitative Study

The aim of the embedded qualitative investigation was to assess the acceptability of the HIP HELPER programme and the research design from the perspective of caregiver dyads and health professionals. Its design was guided by MRC guidance for evaluating complex interventions [21-23], with the intention of understanding contextual factors influencing implementation, theorising how the HIP HELPER programme may work in practice and identifying key uncertainties to enable the programme and research design to be refined.

Six weeks after hospital discharge, caregiver dyads were invited, via a telephone call, to participate in an in-depth, semi-structured interview. They were purposively sampled by age, ethnicity, pre-fracture disability (Nottingham ADL scale (NEADL)[24]), level of cognitive health (AMTS [15]), Disability Assessment for Dementia Scale-6 [25]) and study site. For health professionals, we invited for interview those who had completed the HIP HELPER programme with at least one caregiver-dyad. This sample was purposively sampled by site location and clinical background, to ensure representation across physiotherapy, nursing and occupational therapy professions. All interviews were conducted virtually using Microsoft Teams or telephone. Our topic guide was informed by the MRC guidelines [22,23] and Sekhon's framework of acceptability [26]. All interviews were conducted by the same researcher (AW), an experienced post-doctoral, female, qualitative researcher. AW had no role in recruitment to the study nor intervention delivery. Interviews were audio-recorded, anonymised and transcribed verbatim.

Our analysis took a two-stage approach. Firstly deductive, to assess the quality of implementation and identify contextual factors using the MRC frameworks as a guide [22,23]. An inductive approach further explored participant's experiences and reflections on the intervention from a caregiver dyad perspective. Analysis was independently conducted by one researcher (AW) and then themed with an

For peer review only - http://bmjopen.bmj.com/site/about/guidelines.xhtml

additional two (SH, TS) using Reflexive Thematic Analysis, whereby the highly contextual nature of the data was acknowledged [27,28].

RESULTS

Patient characteristics and treatment

As a result of disruption on NHS services caused by the COVID-19 pandemic, we recruited 102 participants (51 patients; 51 caregivers) from April 2021 to February 2022.

A summary of the patient-cohort characteristics is presented in **Table 1**. Seventy-four percent (37/50) were female, with a mean age of 81.4 years in the intervention group and 77.6 years in the control group. In total, 94% (47/50) were white British or Irish. Ten patient-participants (five per group) had a AMTS [15] of less than eight, indicating cognitive impairment at baseline. The median length of hospital stay was 15 days (IQR: 10, 19) in the intervention group and 11 days in the control group (IQR: 8, 17). As **Table 1** demonstrates, people with hip fracture in the intervention group were older, with more medical co-morbidities and more frequently presented with intra-trochanteric fractures.

A summary of the caregiver-participant characteristics is presented in **Table 2**. Fifty-three percent (36/50) of the cohort were female. Mean age of caregivers in the intervention group was 66 years and 58 years in the control group. Caregivers were most frequently patient-participant's children (53%; 26/50) or a spouse (37%; 18/50). Most caregivers were not working (65%; 32/50); 20% (10/50) were in full-time work.

Feasibility outcomes

The outcomes of the progression criteria traffic-light assessment are presented in **Table 3**.

Recruitment, retention and randomisation acceptability

The CONSORT flow-chart is presented in **Figure 1**. As this illustrates, 1311 potential participants were screened. Of these, 515 (39%; 95% CI: 37% to 42%) were eligible, with 56/515 (11%; 95% CI: 8% to 13%) of eligible participant-dyads consented to participate. Five participant-dyads were withdrawn prior to randomisation. A summary of reasons for being ineligible or being eligible but not consenting are presented in **Supplementary File 2** and **Supplementary File 3**. Recruitment activity per-site is presented in **Supplementary File 4**.

At four-month follow-up, 43/51 participant-dyads (86%) (21 intervention, 22 control) remained in the study. Six participants died, one withdrew without reason; in each instance the complete dyad were withdrawn. At four-months, there were eight patient-participants with cognitive impairment, 35 without cognitive impairment. The groups were largely comparable at baseline (**Table 1**).

Intervention fidelity (health professionals)

Supplementary File 5 illustrate the delivery of the hospital-based and telephone-based HIP HELPER sessions. As summarised in **Supplementary File 6**, 12/25 participant-dyads (48%) of participant-dyads received the minimal compliance level of all three HIP HELPER in-patient sessions and one telephone

For peer review only

call. Reasons for non-compliance were: insufficient staff to deliver the intervention due to staff redeployment and interruption of service provision or visiting of participants due to the COVID-19 pandemic (n=2); patient-participant transferred from a unit (n=2); death (n=4); treatment discontinuation (n=4); or did not answer the telephone (n=3).

Table 4 illustrates that all components of the HIP HELPER programme were delivered during testing. Components which were most frequently delivered were: explanation on recovery expectations (96%; 23/25), goal-setting (92%; 22/25) and pacing and behaviour modification (92%; 22/25). Less frequently delivered components were the more functionally-demanding activities such as washing, dressing and stair and car transfers (38%; 9/25 each).

Intervention fidelity (caregivers)

Only two caregiver-participants returned their caregiver log. Accordingly, there were insufficient data to permit robust assessment of intervention fidelity from the caregiver perspective. This was therefore not analysed.

Contamination

From the qualitative investigations, case report forms for treatment received, protocol deviation reports and delegation logs of treating health professionals, there was no evidence of between-group intervention contamination.

Outcome Data Response Rate

There was limited difference in the completion of the caregiver-participant outcomes at baseline or four months in either group (**Supplementary File 7; Supplementary File 8**). However, there was a notable difference in outcome completion at four months for patients with cognitive impairment and their caregivers. Whilst 80% of caregivers in the control group completed the majority of outcomes, only two caregivers of people with cognitive impairment completed the outcomes (EQ-5D proxy only) in the intervention group. Patient-participants in the intervention group reported a higher response rate to all outcomes at four months except the NEADL[24] (**Supplementary File 7**).

Clinical Outcomes

Supplementary File 9 illustrates the descriptive clinical outcomes presented as median and IQRs for baseline and four-month follow-up. Between-group differences should be interpreted with caution given the level of missing data in both groups (**Supplementary File 7**), a potential baseline difference between the groups for age, presented co-morbidities and fracture type (**Table 1**) and underpowered analyses.

No participant, from either group, experienced a related adverse event or serious adverse event. A summary of the patient-caregiver reported adverse events is presented in **Supplementary File 10**.

For peer review only

Intervention acceptability questionnaire data indicated the HIP HELPER programme was regarded as acceptable by people with hip fracture (**Supplementary File 11**) and caregivers (**Supplementary File 12**).

Qualitative Study

Fourteen caregiver dyads were invited to be interviewed. Ten agreed to participate (Intervention: seven participants; control: three participants). All eight health professionals approached, agreed to be interviewed. **Supplementary File 13** summarises the patient-caregiver's and health professional's characteristics.

Our findings are grouped into three main themes: context, intervention delivery and study procedures.

<u>*Context*</u>

This study was conducted during the COVID-19 pandemic. Patient visitor policies and restrictions were in place. Most caregiver dyads suggested that the opportunity to visit their friend/relative was a main driver for participation.

> *"This trial helped me visit **** once or twice more during her stay in hospital than I would have been allowed to." (*Caregiver 1, Intervention Group, Male, Site 4*)*

For health professionals, allocating visiting time slots created a further challenge of obtaining consent.

> *"With visiting times, we're making sure it's the right carer coming in because it might not be them that have the slots booked that week."* (Occupational Therapist, Female, Site 1).

Changes in visitor policies/restrictions would need to be considered for any future trial.

From the perspective of health professionals, one of the most common reasons for non-participation was attributed to perceived burden on caregivers.

> *"They say, 'oh no that seems like a lot for my daughter to take on or that seems a lot for husband to do they already do enough, or I don't think they'd manage that'. So, it's that perception that they don't want to put any more burden on someone. Seems to be the main reason we find."* (Occupational Therapist, Site 1)

> *"Caregivers who work full-time or spouses who are too frail to make it into hospital just can't."* (Research Physiotherapist, Male, Site 5)

For this study, there was the added complexity of dyad recruitment, as reflected in this comment:

> *"I think getting both the caregiver and the patient consent is a bit of a headache."* (Occupational Therapist, Female, Site 1)

Initially, when health professionals approached the person with hip fracture, a key reason for decline was concerns about feelings of burden on their caregivers. Staff felt recruitment was more successful when the potential caregiver was approached first.

For peer review only

Intervention delivery

Participants perceived the workbook to be helpful in giving a sense of tangible timeframes for recovery. Goal-setting was seen as helping in pushing people out of their comfort zones and allowed them to reflect on progress.

> *"Made us gauge our progress and that he wanted our response to what goals we had. And as I said, going back through it each time we read another page, you realise that we've upped the goal and how far we've progressed"* (Caregiver, Intervention Group, Male, Site 4)

Areas of workbook refinement were identified, such as the volume of information included. Importantly some felt that the workbook did not reflect the life circumstances of younger participants and those still in work.

> "*Like, say, when I did it [the case studies], I was like how am I gonna drag this out for an hour with a patient who can just about get bed to chair*." (Physiotherapist, Male, Site 5)

The follow-up phone calls were seen as helpful. For health professionals, this addition added a rewarding element to their role.

> *"Telephone calls have been really useful. Especially for me because I work on inpatients where don't often get time to follow-up a patient, see how they are, see if there's any concerns. I suppose, from a development point of view, knowing what has worked and what hasn't."* (Physiotherapist, Male, Site 2)

For the patient-caregiver dyads, telephone calls provided encouragement and reassurance to maintain and progress activities.

> *"Reassured me that I'm doing the right things and where I should expect to be."* (Person with Hip Fracture, Intervention Group, Female, Site 4)

Participants perceived value in the telephone calls particularly in navigating additional services and support after discharge. They expressed this would have been challenging without this follow-up.

Study procedures

Respondents repeatedly acknowledged the perceived burden of completing the outcome measures.

> *"It would be a lot for say, the husband to stay at home, trying to fill in 15 pages, when the wife is not there and then they have to try to adjust living by themselves and with all this happening."* (Nurse Practitioner, Female, Site 2)

The outcome measures evaluated patient-caregiver outcomes from a biopsychosocial perspective [29]. Accordingly, some patient reported outcomes posed questions regarding an individual's ability to cope with the physical and psychosocial challenges of trauma. For some respondents, this was reported as emotionally difficult.

> *"So, when you were doing it [questionnaires] with the carer, there was a couple of times where it was uncomfortable. They might not want to say that I have low in mood in front of*

For peer review only

someone. It was quite upsetting for the carer to divulge that information with you, or to bring up things from their past as well." (Physiotherapist, Male, Site 3)

DISCUSSION

The findings from this feasibility study indicate that the HIP HELPER intervention was acceptable to patients, informal caregivers and health professionals but the trial design requires further development to ensure feasibility. Modifications should be made on promoting intervention adherence, prioritising outcome measures to test future effectiveness of data completeness and exploring strategies to support the recruitment of patients with cognitive impairment.

The completion of data from the outcomes at four months was lower than anticipated. This was particularly for participants with cognitive impairment. The qualitative study indicated that participants found the number of outcome measures challenging to complete and future study could better discern what outcome measures are important to people following hip fracture and their caregivers [30]. Streamlining should be made to determine the outcomes which are most valuable to participants and clinical commissioners. A second major modification relates to the acceptability of randomisation. Only 11% of eligible participants were randomised. The qualitative study indicated this may have been because patient-participants did not wish to 'burden their caregiver' with the study when they were initially approached and so declined participation. We originally designed the study approach pre-COVID 19 with an initial approach occurring when both patient and caregiver were together, during visiting hours. This was to facilitate a collaborative decision between the dyad, rather than one member deciding participation. However, due to COVID-19 restrictions on visiting, this was not possible. The qualitative study indicated that the originally planned approach should have been more successful. We recommend that both members of the dyad should be approached simultaneously in future trials, to mitigate such low conversion to randomisation.

A written, information guide about rehabilitation, recovery goals and caregiver responsibilities in the home has been previously reported as valuable to other populations [31]. The findings of this study indicate that whilst the addition of this intervention was beneficial, the HIP HELPER workbook received mixed views from participants. The level of detail, degree of context and order of material covered in the HIP HELPER workbook was considered by many participants as too great. Equally the qualitative findings suggested that the current materials were not representative of all patients and caregivers, most notably younger people who sustain a hip fracture. Further patient and public consultation with the research and clinical hip fracture community is needed to modify this workbook and associated digital offerings of this material.

Approximately 40% of people who sustain a hip fracture present with dementia [32]. Whilst previous authors have acknowledged potential challenges in recruiting people with dementia to drug trials [33], no studies have explored recruitment expectations or strategies to address low recruitment to non-pharmacological interventions [34]. We anticipated recruiting 20 patient-participants with cognitive impairment. In total, 10 participants were recruited with mild cognitive impairment. The qualitative findings suggest that offering further support to research site members who approach patients with cognitive impairment and their caregivers, to promote skills conveying study information, may be beneficial. Furthermore, given the poor response rate in four-month outcome data for these participants, consideration on the appropriateness of the current instruments used and model of delivery of outcome battery for people with cognitive impairment and their caregiver, should be considered in future trials of this population [35].

For peer review only

Previous evidence suggests that health professionals have been inflexible about people with hip fracture and their caregivers to discuss care plans, when these do happen [36-38]. The qualitative study highlighted that those participants who received the HIP HELPER programme appreciated the contact and opportunity to explore skills and knowledge for early recover and caregiver support following hip fracture. The addition of the telephone calls was reported as offering beneficial, additional, post-discharge support in a flexible approach. However, intervention fidelity was lower than anticipated. Unfortunately, it is difficult to separate the challenges which COVID-19 placed on research conduct and service provision and challenges in delivering the HIP HELPER programme in a non-health crisis. Sites were challenged in delivering the intervention due to staffing, patient transfers, visiting restrictions and earlier than planned discharges. This impacted on fidelity of the 'full' HIP HELPER programme to all participants. Deeper exploration on modification to intervention delivery and what components are 'core' ingredients to the programme to estimate compliance thresholds would be warranted.

This study presented with strengths and limitations. A notable strength was the ability to recruit over 100 participants from five NHS organisations during the 2020 COVID-19 pandemic. Whilst a short-fall of 10 participant-dyads, given the challenges in managing site opening and research conduct during the COVID-19 pandemic, the ability to undertake this was considered a success. In the absence of COVID-19 (or such like) restrictions on visiting, patient flow and research activity in NHS settings, we would anticipate that this impact would be negated in future trials of this population. Secondly, although we planned to assess whether caregivers adopted their caregiving knowledge from the intervention into the home environment, only two participants returned these data. This was a major limitation and resulted in an inability to answer an *a prior* progression criterion. The findings from the qualitative study and acceptability questionnaires may suggest carry-over of the intervention into practice. However, we acknowledge that this does not offer the granularity of detail which the original caregiver log would have conveyed. Finally, the follow-up rates and data competition for clinical outcomes were low. Accordingly, it was not possible to confidently assess for a signal of efficacy in the experimental intervention. Further modifications in what and how clinical outcome data are collected should be considered as part of the following work to improve the feasibility of this trial design.

CONCLUSIONS

The HIP HELPER programme was acceptable to participants and health professions. Further modifications to the trial design are needed to ensure feasibility. These findings will form the basis of reflection and refinement to the trial design to test the clinical and cost-effectiveness of the programme in addition to understand the scalability and pathway to implementation.

For peer review only

ACKNOWLEDGEMENTS AND DECLARATIONS

Collaborators: The HIP HELPER Study Collaborators: Penny Clifford (Norfolk, PPI Representative), Lis Freeman (Norfolk, PPI Representative), Rene Gray (Principal Investigator – James Paget University Hospital NHS Trust), Yan Cunningham (Principal Investigator – City Hospitals Sunderland NHS Foundation Trust), Sarah Langford (Principal Investigator - Northumbria Healthcare NHS Foundation Trust), Dr Mark Baxter (Principal Investigator - University Hospital Southampton NHS Foundation Trust), Jessica Pawson – (Principal Investigator - Barts Health NHS Trust), Melissa Taylor (James Paget University Hospital NHS Trust), Anna Mellows (James Paget University Hospital NHS Trust), Kate Lacey (James Paget University Hospital NHS Trust), Alex Herring (City Hospital Sunderland NHS Foundation Trust), Diane Williams (Northumbria Healthcare NHS Foundation Trust), Anna Cromie (Northumbria Healthcare NHS Foundation Trust), Gail Menton (Northumbria Healthcare NHS Foundation Trust), Warren Corbett (University Hospital Southampton NHS Foundation Trust), Helen Jowett (University Hospital Southampton NHS Foundation Trust), Vishwanath Joshi (Barts Health NHS Trust), Maninderpal Matharu (Barts Health NHS Trust), Maria Baggot (University Hospital Southampton NHS Foundation Trust),and David Barker (University Hospital Southampton NHS Foundation Trust).

Oversight Committee Membership: TOC Members: Associate Professor Susan Dutton (Chair; University of Oxford, UK), Professor Opinder Sahota (University of Nottingham, UK), Dr Katie Sheehan (Kings College London, UK).

Authors' Contributors: TS, SHo, SHa, PA, AC, KG, KP, PL, MCo, SEL, MCr researched the topic and devised the study. TS, SHa, PA, RK, AC, KG, SHo, AW, KP, PL, MCo, SEL, MCr provided the first draft of the manuscript. AC provided statistical oversight. TS, SHa, PA, RK, AC, KG, AW, SHo, KP, PL, MCo, SEL, MCr contributed equally to manuscript preparation. TS acts a guarantor.

Funding Statement: This project is funded by the National Institute for Health and Care Research (NIHR) under its Research for Patient Benefit (RfPB) Programme (Grant Reference Number NIHR200731). SL role in this study was supported by the National Institute for Health and Care Research Exeter Biomedical Research Centre. The views expressed are those of the author(s) and not necessarily those of the NIHR or the Department of Health and Social Care.

Competing Interests Statement: None declared.

Disclaimer: MCo and SHo are supported by the National Institute for Health Research (NIHR) Oxford Biomedical Research Centre (BRC). SL is supported by the National Institute for Health and Care Research Exeter Biomedical Research Centre. The views expressed are those of the author(s) and not necessarily those of the NHS, the NIHR or the Department of Health and Social Care.

Ethics Approval: North East - Newcastle & North Tyneside 1 Research Ethics Committee (20/NE/0213) Date: 16 March 2021.

Trial Sponsor: University of East Anglia (Research Sponsor, Research and Innovation Services, The Registry, University of East Anglia, Norwich, NR4 7TJ; Email: researchsponsor@uea.ac.uk) The views expressed are those of the author(s) and not necessarily those of the sponsor.

Provenance and Peer Review: Not commissioned; externally peer reviewed.

Data Availability Statement:
Data are available upon reasonable request. This included access to the full protocol, anonymised participant level dataset and statistical code. Access to the de-identified dataset for purposes of

For peer review only

research other than this study, would be at the discretion of the Chief Investigator, Professor Toby Smith and Norwich CTU. Requests for the de-identified dataset generated during the current study should be made to the Chief Investigator, Professor Toby Smith (email: toby.o.smith@warwick.ac.uk) or Norwich CTU (NorwichCTU@uea.ac.uk). Professor Toby Smith and Norwich CTU will consider requests once the main results from the study have been published up until 31 December 2028.

Patient consent for publication: Not required.

For peer review only

FIGURE AND TABLE LEGENDS

Table 1: Demographic characteristics of the patient participants at baseline.

Table 2: Demographic characteristics of the caregiver participants at baseline.

Table 3: Progression criteria traffic-light summary table.

Table 4: Table illustrating the frequency to-which the components of the HIP HELPER intervention were delivered to participants.

Figure 1: CONSORT diagram reporting the flow of patient-caregiver participants in the HIP HELPER study.

Supplementary File 1: Clinical outcome data collected for patient-participants and caregiver-participants at baseline and four-months post-randomisation.

Supplementary File 2: Reasons for ineligibility in the HIP HELPER study across the five sites.

Supplementary File 3: Reasons for eligible participants not consenting to the HIP HELPER study across the five sites.

Supplementary File 4: Recruitment rate presented by each of the five HIP HELPER sites.

Supplementary File 5: Table illustrating the number of participant-dyads that attended intervention sessions.

Supplementary File 6: Intervention fidelity by site (shown below are those that achieved intervention fidelity).

Supplementary File 7: Table illustrating the data completion of clinical outcome scores (baseline and 4-month follow-up).

Supplementary File 8: Carer Resource Utilisation in Dementia questionnaire completion statistics at baseline and 4-months.

Supplementary File 9: Table illustrating descriptive clinical outcomes presented as median and inter-quartile ranges (baseline and 4-month follow-up).

Supplementary File 10: Acceptability questionnaire (participant) by question number.

Supplementary File 11: Acceptability questionnaire (caregiver) by question number.

Supplementary File 12: Summary of safety outcomes at the end of the study for all participants.

Supplementary File 13: Characteristics of qualitative investigation sample.

For peer review only

REFERENCES

1. National Hip Fracture Database 2023. Assessment benchmark summary 2022. Accessed: 16th February 2023. Available at: https://www.nhfd.co.uk/tables
2. Mitchell P, Bateman K. Dementia, falls and fractures. Integrated approaches to improve quality and reduce costs. UK: Novartis; 2012.
3. Dyer SM, Dyer SM, Crotty M, Fairhall N, Magaziner J, Beaupre LA, Cameron ID, Sherrington C; Fragility Fracture Network (FFN) Rehabilitation Research Special Interest Group. A critical review of the long-term disability outcomes following hip fracture. *BMC Geriatr* 2016;**16**:158.
4. Lawler K, Taylor NF, Shields N. Involving family members in physiotherapy for older people transitioning from hospital to the community: a qualitative analysis. *Disabil Rehabil* 2015;**37**:2061-9.
5. Johnell O, Kanis JA. An estimate of the worldwide prevalence, mortality and disability associated with hip fracture. *Osteopor Int* 2004;**15**:897-902.
6. Griffin XL, Parsons N, Achten J, Fernandez M, Costa ML. Recovery of health-related quality of life in a United Kingdom hip fracture population. The Warwick Hip Trauma Evaluation--a prospective cohort study. *Bone Joint J* 2015;**97-B**:372-82.
7. Nurmi I, Narinen A, Lüthje P, Tanninen S. Cost analysis of hip fracture treatment among the elderly for the public health services: a 1-year prospective study in 106 consecutive patients. *Arch Orthop Trauma Surg* 2003;**123**:551-4.
8. Ariza-Vega P, Ortiz-Piña M, Kristensen MT, Castellote-Caballero Y, Jiménez-Moleón JJ. High perceived caregiver burden for relatives of patients following hip fracture surgery. *Disabil Rehabil* 2017;**16**:1-8
9. Wimo A, Wetterholm AL, Mastey V, Winblad B. Evaluation of the resource utilization and caregiver time in Anti-dementia drug trials - a quantitative battery. In: Wimo A, Karlsson G, Jönsson B, Winblad B (eds). The Health Economics of dementia, 1998. Wiley's, London, UK
10. Saletti-Cuesta L, Tutton E, Langstaff D, Willett K. Understanding informal carers' experiences of caring for older people with a hip fracture: a systematic review of qualitative studies. *Disabil Rehabil* 2018;**40**:740-50.
11. Asif M, Cadel L, Kuluski K, Everall AC, Guilcher SJT. Patient and caregiver experiences on care transitions for adults with a hip fracture: a scoping review. *Disabil Rehabil* 2020;**42**:3549-58.
12. Giosa JL, Stolee P, Dupuis SL, Mock SE, Santi SM. Examination of family caregiver experiences during care transitions of older adults. *Can J Aging* 2014;**33**:137-53.
13. Eldridge SM, Chan CL, Campbell MJ, Bond CM, Hopewell S, Thabane L, Lancaster GA; PAFS consensus group. CONSORT 2010 statement: extension to randomised pilot and feasibility trials. *BMJ* 2016;**355**:i5239.
14. Smith T, Clark L, Khoury R, Man MS, Hanson S, Welsh A, Clark A, Hopewell S, Pfeiffer K, Logan P, Crotty M, Costa M, Lamb SE. A feasibility study to assess the design of a multicentre randomized controlled trial of the clinical and cost-effectiveness of a caregiving intervention for people following hip fracture surgery. *Bone Jt Open* 2021;**2**:909-20.
15. Hodkinson KM. Evaluation of a mental test score for assessment of mental impairment in the elderly. *Age Ageing* 1972;**4**:233-8.
16. National Institute for Health and Care Excellence. Hip fracture: management. Clinical guideline CG124 - 2023. Available at: https://www.nice.org.uk/Guidance/CG124 Accessed on: 16 February 2023.
17. Bandura A. Self-efficacy: towards a unifying theory of behavioural change. *Psychol Rev* 1977; **84**:191–215.
18. Saklad M. Grading of patients for surgical procedures. *Anesthesiology* 1941;**2**:281-4.

19. Teare MD, Dimairo M, Shephard N, Hayman A, Whitehead A, Walters SJ. Sample size requirements to estimate key design parameters from external pilot randomised controlled trials: a simulation study. *Trials* 2014;**15**:264
20. Avery KN, Williamson PR, Gamble C, O'Connell Francischetto E, Metcalfe C, Davidson P, Williams H, Blazeby JM; members of the Internal Pilot Trials Workshop supported by the Hubs for Trials Methodology Research. Informing efficient randomised controlled trials: exploration of challenges in developing progression criteria for internal pilot studies. *BMJ Open* 2017;**7**:e013537.
21. Craig P, Dieppe P, Macintyre S, Michie S, Nazareth I, Petticrew M; Medical Research Council Guidance. Developing and evaluating complex interventions: the new Medical Research Council guidance. *BMJ* 2008;**337**:a1655.
22. Moore GF, Audrey S, Barker M, Bond L, Bonell C, Hardeman W, et al. Process evaluation of complex interventions: Medical Research Council guidance. *BMJ* 2015;**350**:h1258.
23. Skivington K, Matthews L, Simpson SA, Craig P, Baird J, Blazeby JM, Boyd KA, Craig N, French DP, McIntosh E, Petticrew M, Rycroft-Malone J, White M, Moore L. A new framework for developing and evaluating complex interventions: update of Medical Research Council guidance. *BMJ* 2021;**374**:n2061.
24. Gladman JR, Lincoln NB, Adams SA. Use of the extended ADL scale with stroke patients. *Age Ageing* 1993;**22**:419–24.
25. de Rotrou J, Wu YH, Hugonot-Diener L, Thomas-Antérion C, Vidal JS, Plichart M, Rigaud AS, Hanon O. DAD-6: A 6-Item version of the Disability Assessment for Dementia scale which may differentiate Alzheimer's disease and mild cognitive impairment from controls. *Dement Geriatr Cogn Disord* 2012;**33**:210-8.
26. Sekhon M, Cartwright M, Francis JJ. Acceptability of healthcare interventions: an overview of reviews and development of a theoretical framework. *BMC Health Serv Res* 2017;**17**:88.
27. Braun V, Clarke V. Using thematic analysis in psychology. *Qual Res Psychol* 2006;**3**:77-101.
28. Braun V, Clarke V. One size fits all? What counts as quality practice in (reflexive) thematic analysis? *Qual Res Psychol* 2020;**3**:1-25.
29. Engel GL. The need for a new medical model: a challenge for biomedicine. *Science* 1977;**196**:129-36.
30. Treweek S, Miyakoda V, Burke D, Shiely F. Getting it wrong most of the time? Comparing trialists' choice of primary outcome with what patients and health professionals want. *Trials* 2022;**23**:537.
31. Schiller C, Franke T, Belle J, Sims-Gould J, Sale J, Ashe MC. Words of wisdom - patient perspectives to guide recovery for older adults after hip fracture: a qualitative study. *Patient Prefer Adherence* 2015;**9**:57-64.
32. Seitz DP, Adunuri N, Gill SS, Rochon PA. Prevalence of dementia and cognitive impairment among older adults with hip fractures. *J Am Med Direct Assoc* 2011;**12**:556-64.
33. Cooper C, Ketley D, Livingston G. Systematic review and meta-analysis to estimate potential recruitment to dementia intervention studies. *Int J Geriatr Psychiatry* 2014;**29**:515-25.
34. Brambilla M, Parra MA, Della Sala S, Alemanno F, Pomati S. Challenges to recruitment of participants with MCI in a multicentric neuropsychological study. *Aging Clin Exp Res* 2021;**33**:2007-10.
35. Kramer JM, Schwartz A. Reducing barriers to patient-reported outcome measures for people with cognitive impairments. *Arch Phys Med Rehabil* 2017;**98**:1705-15.
36. Elliott J, Forbes D, Chesworth BM, Ceci C, Stolee P. Information sharing with rural family caregivers during care transitions of hip fracture patients. *Int J Integr Care* 2014;**14**:e018.

For peer review only

37. Glenny C, Stolee P, Sheiban L, Jaglal S. Communicating during care transitions for older hip fracture patients: family caregiver and health care provider's perspectives. *Int J Integr Care* 2013;**13**:e044.
38. Nahm ES, Resnick B, Orwig D, Magaziner J, Degrezia M. Exploration of informal caregiving following hip fracture. *Geriatr Nurs* 2010;**31**:254-62.

.

.

For peer review only

Table 1: Demographic characteristics of the patient participants at baseline.

	Intervention (N=25)	Control (N=25)
Gender: n (%)		
Male	4 (16.0)	9 (36.0)
Female	21 (84.0)	16 (64.0)
Age in years: mean (SD)	81.4 (8.1)	77.6 (8.6)
Ethnicity: n (%)		
White British	22 (88.0)	23 (92.0)
White Irish	1 (4.0)	1 (4.0)
Indian	0	1 (4.0)
Bangladeshi	2 (8.0)	0
Height (cm): mean (SD)	164.2 (10.6)	166.81 (10.0)
Weight (kg): mean (SD)	65.1 (13.9)	72.6 (17.2)
BMI: mean (SD)	24.1 (4.5)	26.1 (6.2)
AMTS score: median (IQR)	10 (9, 10)	10 (9, 10)

AMTS category		
Cognitive Impairment (score<8)	5 (20.0)	5 (20.0)
No Cognitive Impairment (score≥8)	20 (80.0)	20 (80.0)
Side of hip fracture: n (%)		
Left	10 (40.0)	15 (60.0)
Right	15 (60.0)	10 (40.0)
Hip Fracture classification: n (%)		
Intra-capsular	14 (56.0)	17 (68.0)
Intertrochanteric	8 (32.0)	5 (20.0)
Sub-trochanteric	3 (12.0)	3 (12.0)
Operative Procedure: n (%)		
Hemiarthroplasty	10 (41.7)	15 (60.0)
THR	2 (8.3)	1 (4.0)
Cannulated screws	3 (12.5)	1 (4.0)
DHS	4 (16.7)	5 (20.0)
Intramedullary device	5 (20.8)	3 (12.0)
Missing	1	0
Length of hospital stay (days): median (IQR)	15 (10, 19)	11 (8, 17)
ASA Grade: median (IQR)	3 (3, 3)	3 (2, 3)
Missing	1	1
Current Medical Diagnoses: n (%)		
Cardiac	8 (32.0)	5 (20.0)
Asthma	2 (8.0)	1 (4.0)
COPD	6 (24.0)	7 (28.0)
Hypertension	12 (48.0)	14 (56.0)
Diabetes	2 (8.0)	5 (20.0)
Stroke	0	2 (8.0)
Cancer	7 (28.0)	3 (12.0)
Osteoarthritis	5 (20.0)	3 (12.0)
Low back pain	4 (16.0)	3 (12.0)
Depression	0	0
Anxiety	0	0
Dementia	2 (8.0)	2 (8.0)
Other	12 (48.0)	11 (44.0)

AMTS – Abbreviated Mental Test Score; ASA - American Society of Anesthesiologists OPD – chronic obstructive pulmonary disease; BMI – body mass index; cm – centimetres; DHS – dynamic hip screw; IQR – inter-quartile range; kg – kilograms; SD – standard deviation; THR – total hip replacement

For peer review only

Table 2: Demographic characteristics of the caregiver participants at baseline.

	Intervention (N=25)	Control (N=25)
Gender: n (%)		
Male	14 (56.0)	9 (37.5)
Female	11 (44.0)	15 (62.5)
Missing	0	1
Age in years: mean (SD)	66.2 (13.6)	57.7 (12.9)
Missing	1	2
Ethnicity: n (%)		
White British	20 (80.0)	23 (95.8)
White Irish	1 (4.0)	0
White Other	1 4.0)	1 (4.2)
Mixed - Other	1 (4.0)	0
Bangladeshi	2 (8.0)	0
Missing	0	1
AMTS score: median (IQR)	10 (10, 10)	10 (10, 10)
Missing	0	1
Relationship to participant: n (%)		
Spouse	10 (40.0)	8 (33.3)
Daughter/Son	13 (52.0)	13 (54.2)
Grandchild	0	1 (4.2)
Other	2 (8.0)	2 (8.3)
Missing	0	1
Caregiver living with participant: n (%)		
Yes	16 (69.6%)	14 (60.9%)
No	7 (30.4%)	9 (39.1%)
Missing	2	2
Occupation: n (%)		
Not working	17 (68.0)	15 (62.5)
Part-time	3 (12.0)	4 (16.7)
Full-time	5 (20.0)	5 (20.8)
Missing	0	1
Current Medical Diagnoses: n (%)		
Cardiac	2 (8.0)	1 (4.0)
Asthma	0	4 (16.0)
COPD	0	0
Hypertension	2 (8.0)	1 (4.0)
Diabetes	1 (4.0)	2 (8.0)
Stroke	0	0
Cancer	1 (4.0)	0
Osteoarthritis	3 (12.0)	1 (4.0)
Low back pain	3 (12.0)	4 (16.0)
Depression	0	3 (12.0)
Anxiety	0	6 (24.0)
Other	3 (12.0)	2 (8.0)

AMTS – Abbreviated Mental Test Score; COPD – chronic obstructive pulmonary disease; SD – standard deviation

For peer review only

Table 3: Progression criteria traffic-light summary table.

	Green (Go)	Amber (Amend)	Red (Stop)	Judgement
**Recruitment**	> 40% of patients screened would be eligible	30% to 40% would be eligible	< 30% would be eligible	39% participants were eligible
**Randomisation acceptability**	> 40% of eligible patients consent to be randomised	20% to 40% would be randomised	< 20% would be randomised	11% of eligible participants were randomised
**Intervention fidelity (healthcare professionals)**	> 70% of participants compliant with their allocated intervention as randomised	50% to 70% received intervention as randomised	< 50% received intervention as randomised	48% received 'complete intervention' as randomised, limited by COVID-19
**Intervention fidelity (caregivers)**	> 90% of participants adopted HIP HELPER intervention post-discharge	60% to 90% adopted HIP HELPER post-discharge	< 60% adopted HIP HELPER post-discharge	Unable to assess with insufficient caregiver logs
**Contamination**	< 5% of participants in either group received majority of their allocated treatment crossover	5% to 10% of participants crossover	> 10% of participants crossover	0% evidence of contamination

For peer review only

Table 4: Table illustrating the frequency to-which the components of the HIP HELPER intervention were delivered to participants.

Item	Intervention (N=25)			
	Session 1 N (%)	Session 2 N (%)	Session 3 N (%)	At least one occurrence during Sessions 1-3
Practical skills-transfers:				
Bed to chair	16 (66.7)	13 (68.4)	8 (57.1)	20 (83.3)
Toilet	4 (16.7)	7 (36.8)	4 (28.6)	12 (50.0)
Walking and walking aids	15 (62.5)	12 (63.2)	9 (64.3)	19 (79.2)
In/out bed	16 (66.7)	12 (63.2)	8 (57.1)	20 (83.3)
Car	3 (12.5)	6 (31.6)	2 (14.3)	9 (37.5)
Stairs	3 (12.5)	6 (31.6)	3 (21.4)	9 (37.5)
Goal Setting theory	21 (87.5)	13 (68.4)	4 (28.6)	22 (91.7)
Goal setting practice	15 (62.5)	15 (79.0)	6 (42.9)	22 (91.7)
Pacing and behaviour theory	20 (83.3)	9 (47.4)	6 (42.9)	22 (91.7)
Pacing and behaviour task	13 (54.2)	10 (52.6)	5 (35.7)	18 (75.0)
Expectations of recovery pathways	22 (91.7)	9 (47.4)	5 (35.7)	23 (95.8)
Practical skills:				
Washing	1 (4.2)	7 (36.8)	1 (7.1)	9 (37.5)
Dressing	1 (4.2)	9 (47.4)	1 (7.1)	9 (37.5)
Caregiver:				
Management discussion	3 (12.5)	7 (36.8)	14 (100)	18 (75.0)
Pacing discussion	4 (16.7)	6 (31.6)	10 (71.4)	16 (66.7)
Case scenario discussion	1 (4.2)	3 (15.8)	9 (64.3)	12 (50.0)
Provision and discussion on HIP HELPER manual	15 (62.5)	4 (21.1)	10 (71.4)	22 (91.7)
Confirmation of HIP HELPER telephone calls	0	0	13 (92.9)	13 (54.2)
Other 1: ("pain relief", "lack of hip precautions", "caregiver questions", "equipment ordering")	1 (4.2)	2 (10.5)	1 (7.1)	2 (8.3)
Other 2: ("equipment ordering")	0	0	1 (7.1)	1 (4.2)
Other 3: ("sleep")	0	0	1 (7.1)	1 (4.2)

Note: Number of planned sessions not performed were: one Session 1, six Session 2, 11 Session 3; Missing data not given, percentages are out of non-missing data.

For peer review only

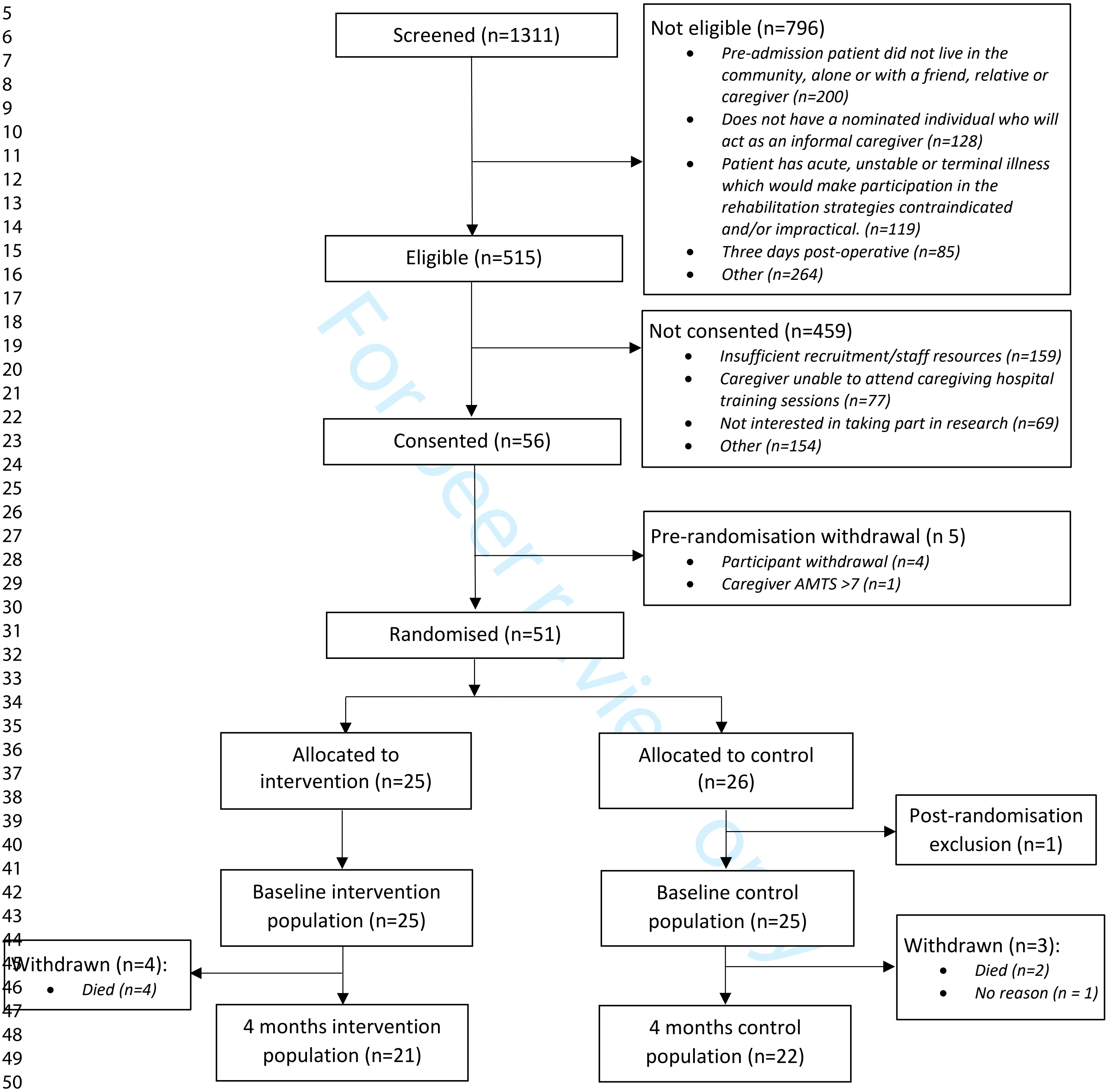
Screened (n=1311)
Not eligible (n=796)
Pre-admission patient did not live in the community, alone or with a friend, relative or caregiver (n=200)
Does not have a nominated individual who will act as an informal caregiver (n=128)
Patient has acute, unstable or terminal illness which would make participation in the rehabilitation strategies contraindicated and/or impractical. (n=119)
Three days post-operative (n=85)
Other (n=264)
Eligible (n=515)
Not consented (n=459)
Insufficient recruitment/staff resources (n=159)
Caregiver unable to attend caregiving hospital training sessions (n=77)
Not interested in taking part in research (n=69)
Other (n=154)
Consented (n=56)
Pre-randomisation withdrawal (n 5)
Participant withdrawal (n=4)
Caregiver AMTS >7 (n=1)
Randomised (n=51)
Allocated to intervention (n=25)
Allocated to control (n=26)
Post-randomisation exclusion (n=1)
Baseline intervention population (n=25)
Baseline control population (n=25)
Withdrawn (n=4):
Died (n=4)
Withdrawn (n=3):
Died (n=2)
No reason (n = 1)
4 months intervention population (n=21)
4 months control population (n=22)

Supplementary File 1: Clinical outcome data collected for patient-participants and caregiver-participants at baseline and four-months post-randomisation.

Patients without cognitive impairment:

- EQ-5D-5L[38]
- Nottingham Activities of Daily Living Scale (NEADL)[24]
- General Self-Efficacy questionnaire
- Center for Epidemiologic Studies Depression Scale (CES-D)
- Numerical rating scale (NRS) for pain (hip and whole body)
- Complications and adverse events including mortality

For all caregivers:

- EQ-5D-5L[38]
- CES-D
- Short Sense of Competence Questionnaire for caregiver burden (SCQ-16)
- Resource Utilization in Dementia questionnaire[9]
- Complications and adverse events including mortality
- Patient and caregiver residential status

PLUS for caregivers of patients with cognitive impairment

- EQ-5D-5L proxy[38]
- Disability Assessment for Dementia Scale-6 (DADS-6) functional score[25]
- Neuropsychiatry Inventory (NPI)
- Abbey Pain Scale

For peer review only

Supplementary File 2: Reasons for ineligibility in the HIP HELPER study across the five sites.

Site 1	Site 2	Site 3	Site 4	Site 5	Total	Screening	
37	38	11	83	31	200	Pre-admission patient did not live in the community, alone or with a friend, relative or caregiver.	Ineligible Reason
51	22	2	16	37	128	Does not have a nominated individual who will act as an informal caregiver.	
45	12	7	22	33	119	Patient has acute, unstable or terminal illness which would make participation in the rehabilitation strategies contraindicated and/or impractical.	
-	-	-	85	-	85	3+ days post op	
12	1	5	9	27	54	Patients expected by the clinical team to be discharged to a care home (residential or nursing) after their hospital admission	
15	1	1	17	9	43	Patient under age of 65 years	
7	4	-	29	1	41	Patient not willing or able to provide consent or assent depending on the level of cognitive impairment.	
8	1	6	15	8	38	Other	
8	4	-	21	-	33	Caregiver not willing or able to provide consent	
3	2	-	12	2	19	Not undergone hip fracture surgery	
-	-	-	11	2	13	Missing reason	
-	1	-	4	4	9	Participant has significant difficulties reading and/or comprehending English	
-	-	-	2	7	9	Patient under age of 65 years	
-	1	-	-	2	3	Individual caregivers unable to understand written English or have access to a translator.	
1	-	1	-	-	2	Principal (main) caregiver has AMTS score of less than 8	
					**796**	**Total ineligible**	

For peer review only

Supplementary File 3: Reasons for eligible participants not consenting to the HIP HELPER study across the five sites.

Site 1	Site 2	Site 3	Site 4	Site 5	Total	Not Consented	
47	26	15	43	27	158	Insufficient recruitment staff resources	Eligible but not consented
10	25	2	16	23	76	Caregiver unable to attend caregiving hospital training sessions	
15	11	6	27	11	70	Not interested in taking part in research	
6	9	1	25	14	55	Other	
21	-	3	13	1	38	Leaving the area	
3	13	1	7	4	28	Missing Reason	
19	-	-	-	1	19	Outlier ward	
-	-	-	-	-	4	Does NOT want to be randomised to receive caregiver intervention	
-	3	1	-	4	4	Persistent post-operative confusion	
-	1	1	-	-	2	Does not have a nominated individual who will act as an informal caregiver.	
-	-	-	-	-	2	Does NOT want to be randomised to receive control (usual care)	
-	-	-	1	2	2	Caregiver not willing or able to provide consent	
-	1	-	-	1	1	Participant has significant difficulties reading and/or comprehending English	
					**459**	**Total not consented**	
-	-	-	-	-	-	Post-operative complication	Not Randomised
	-	-	-	-	1	Persistent post-operative confusion	
-	-	-	-	-	-	Participant no longer wants to take part in research	
-	-	-	-	-	-	Unable to approach caregiver	
-	-	-	-	-	-	Insufficient recruitment staff resources	
2	1	-	-	1	4	Withdrawn	
					**5**	**Total not randomised**	

For peer review only

Supplementary File 4: Recruitment rate presented by each of the five HIP HELPER sites.

Site	Date Opened	Number of Recruited Participants											Total
		**April 2021**	**May 2021**	**June 2021**	**July 2021**	**August 2021**	**Sept 2021**	**Oct 2021**	**Nov 2021**	**Dec 2021**	**Jan 2022**	**Feb 2022**	
**Site 1**	07Apr2021	1	3	0	1	1	1	3	0	0			10
**Site 2**	19Apr2021		1	2	2	0	1	2	0	0	0	0	8
**Site 3**	15Jun2021				1	0	0	0	1	1	0	2	5
**Site 4**	29Jun2021				3	1	2	2	0	2	2	1	13
**Site 5**	05Aug2021					0	3	3	4	2	1	2	15
**TOTAL**		**1**	**4**	**2**	**7**	**2**	**7**	**10**	**5**	**5**	**3**	**5**	**51**

For peer review only

Supplementary File 5: Table illustrating the number of participant-dyads that attended intervention sessions.

	Hospital-Based Session			Telephone Booster Calls		
	Session 1 N (%)	Session 2 N (%)	Session 3 N (%)	Telephone 1 N (%)	Telephone 2 N (%)	Telephone 3 N (%)
Yes	24 (96.0)	19 (76.0)	14 (56.0)	13 (54.2)	14 (58.3)	11 (47.8)
No	1 (4.0)	6 (24.0)	11 (44.0)	11 (45.8)	10 (41.7)	12 (52.2)
Missing	0	0	0	1	1	2

For peer review only

Supplementary File 6: Intervention fidelity by site (shown below are those that achieved intervention fidelity).

	Site Number					All Sites
	**1**	**2**	**3**	**4**	**5**	
Intervention fidelity n (%)	2 (40.0)	2 (50.0)	0	3 (50.0)	5 (62.5)	12 (48.0)

N.B. fidelity = 3 in-patient sessions and >= 1 telephone calls

For peer review only

Supplementary File 7: Table illustrating the data completion of clinical outcome scores (baseline and 4-month follow-up).

	**Baseline (N; %)**		**4-Month Follow-up (N; %)**	
	**Intervention**	**Control**	**Intervention**	**Control**
Patient participants without Cognitive Impairment (n=41)				
EQ-5D-5L Index	20 (100)	19 (95.0)	14 (70.0)	11 (57.9)
EQ-5D-5L VAS	19 (95.0)	18 (90.0)	14 (70.0)	12 (63.2)
NEADL	15 (75.0)	17 (85.0)	5 (27.8)	9 (52.9)
GSE	19 (95.0)	19 (95.0)	11 (61.1)	10 (58.8)
CES-D	17 (85.0)	16 (80.0)	11 (61.1)	9 (52.9)
NRS pain – hip	20 (100)	18 (90.0)	12 (66.7)	10 (58.8)
NRS pain - body	19 (95.0)	19 (95.0)	12 (66.7)	10 (58.8)
Patient participants with Cognitive Impairment (n=10)				
EQ5D Proxy Index	5 (100)	5 (100)	2 (40.0)	4 (80.0)
EQ5D Proxy VAS	5 (100)	5 (100)	2 (40.0)	4 (80.0)
DADS-6 total (carer)	3 (60.0)	5 (100)	0	4 (80.0)
DADS-6 initiation (carer)	4 (80.0)	5 (100)	0	4 (80.0)
DADS-6 planification (carer)	3 (60.0)	5 (100)	0	4 (80.0)
DADS-6 performance (carer)	4 (80.0)	5 (100)	0	4 (80.0)
NPI severity (carer)	5 (100)	5 (100)	0	3 (60.0)
NPI distress (carer)	5 (100)	5 (100)	0	3 (60.0)
Abbey Pain Scale (carer)	2 (40.0)	5 (100)	0	3 (60.0)
NRS pain – hip	4 (80.0)	4 (80.0)	0	4 (80.0)
NRS pain - body	4 (80.0)	4 (80.0)	0	4 (80.0)
Caregiver participants (n=51)				
EQ-5D-5L Index	24 (96.0)	23 (92.0)	10 (47.6)	11 (50.0)
EQ-5D-5L VAS	24 (96.0)	23 (92.0)	12 (57.1)	12 (54.6)
CESD	20 (80.0)	20 (80.0)	12 (57.1)	9 (40.9)
SCQ total	23 (92.0)	20 (80.0)	12 (57.1)	11 (50.0)
SCQ recipient satisfaction	24 (96.0)	22 (88.0)	12 (57.1)	12 (54.6)
SCQ own satisfaction	23 (92.0)	21 (84.0)	12 (57.1)	12 (54.6)
SCQ consequence	24 (96.0)	23 (92.0	12 (57.1)	11 (50.0)
Patient living in own home: n (%)	24 (96.0)	23 (92.0)	11 (52.4)	11 (50.0)
Caregiver living with participant: n (%)	23 (92.0)	23 (92.0)	12 (57.1)	12 (54.5)

CESD - Center for Epidemiologic Studies Depression Scale; DAD-6 – Disability Assessment for Dementia scale – 6 item; GSE – Generalized Self-Efficacy Scale; NA – not assessed; NEADL - Nottingham Extended Activities of Daily Living scale (NEADL); NPI - Neuropsychiatry Inventory; NRS – numerical rating scale; SCQ – Short Sense of Competence questionnaire for caregiver burden; VAS – visual analogue scale
NB: Deaths prior to 4 month follow-up were assigned zero response accounting for 40% response rate for 2 participants in the patient participants with cognitive impairment responses.

For peer review only

Supplementary File 8: Carer Resource Utilisation in Dementia questionnaire completion statistics at baseline and 4-months

C-RUD Question	Conditional on previous question responses	Baseline		Conditional on previous question responses	4-Months	
		Intervention N=25 n (%)	Control N=25 n (%)		Intervention N=21 n (%)	Control N=22 n (%)
1. Age	No	24 (96.0)	23 (92.0)	No	12 (57.1)	12 (54.6)
2. Gender	No	24 (96.0)	23 (92.0)	No	12 (57.1)	12 (54.6)
3. Relationship to patient	No	23 (92.0)	23 (92.0)	No	12 (57.1)	12 (54.6)
4. Children living with you	No	24 (96.0)	23 (92.0)	No	12 (57.1)	12 (54.6)
5. Do you live with the patient	No	23 (92.0)	23 (92.0)	No	12 (57.1)	12 (54.6)
6. Other caregivers involved	No	23 (92.0)	23 (92.0)	No	12 (57.1)	12 (54.6)
7. Your caring contribution level	No	23 (92.0)	23 (92.0)	No	12 (57.1)	12 (54.6)
8. Sleep in last 30 days	No	24 (96.0)	21 (84.0)	No	12 (57.1)	12 (54.6)
9a. Hours per day assisting patient with tasks such as dressing in last 30 days	No	23 (92.0)	20 (80.0)	No	10 (47.6)	11 (50.0)
9b. Days assisting patient with tasks such as dressing in last 30 days	No	23 (92.0)	20 (80.0)	No	10 (47.6)	11 (50.0)
10a. Hours per day assisting patient with tasks such as shopping in last 30 days	No	23 (92.0)	20 (80.0)	No	12 (57.1)	11 (50.0)
10b. Days assisting patient with tasks such as shopping in last 30 days	No	22 (88.0)	21 (84.0)	No	12 (57.1)	11 (50.0)
11a. Hours per day supervising patient in last 30 days	No	21 (84.0)	21 (84.0)	No	11 (52.4)	12 (54.6)
11b. Days supervising patient in last 30 days	No	21 (84.0)	21 (84.0)	No	11 (52.4)	12 (54.6)
12.Work for pay	No	24 (96.0)	23 (92.0)	No	12 (57.1)	11 (50.0)
13. Stop/reduce working	Yes (N=30)	17 (100)	13 (100)	Yes (N=7)	2 (100)	5 (100)
14. Change of job/working situation	Not Assessed in Baseline C-RUD			Yes (N=7)	2 (100)	5 (100)
15. Hours of paid work per week	Yes (N=17)	7 (100)	10 (100)	Yes (N=1)	1 (100)	0
16. Hours of patient care paid per week	Yes (N=17)	7 (100)	10 (100)	Yes (N=1)	1 (100)	0
17. Hours cut for carer responsibilities in last 30 days	Yes (N=17)	7 (100)	10 (100)	Yes (N=1)	1 (100)	0
18a. Work days missed	No	6 (24.0)	11 (44.0)	Yes (N=7)	2 (100)	3 (60.0)
18b. Part work days missed	No	7 (28.0)	11 (44.0)	Yes (N=7)	2 (100)	5 (100)
19. Stop/reduce working	Not Assessed in Baseline C-RUD			Yes (N=1)	1 (100)	0

For peer review only

20. Admitted to hospital in last 30 days	No	24 (96.0)	23 (92.0)	Yes (N=23)	12 (100)	10 (90.9)
21. Nights in each ward in last 30 days	Yes (N=50)	23 (92.0)	23 (92.0)	Yes (N=23)	2 (16.7)	1 (9.1)
22. Hospital ER care in last 30 days	No	23 (92.0)	23 (92.0)	Yes (N=23)	12 (100)	9 (81.8)
23. Health care professional visits in last 30 days	No	24 (96.0)	22 (88.0)	Yes (N=23)	10 (83.3)	10 (90.9)
24. Current medications	No	22 (88.0)	18 (72.0)	No	8 (38.1)	8 (36.4)
25. Patient change of living accommodation since last visit	Not Assessed in Baseline C-RUD			No	12 (57.1)	11 (50.0)
26. Patient current living accommodation	No	24 (96.0)	23 (92.0)	Yes (N=0)	0	0
27. Date living change occurred	Not Assessed in Baseline C-RUD			Yes (N=0)	0	0
28. Reason for living change	Not Assessed in Baseline C-RUD			Yes (N=0)	0	0
29. Who patient lives with	No	24 (96.0)	23 (92.0)	Not Assessed in Follow-Up C-RUD		
30. Patient temporary accommodation in last 30 days	No	7 (28.0)	6 (24.0)	Yes (N=23)	8 (38.1)	5 (45.5)
31. Patient admitted to hospital in last 30 days	No	23 (92.0)	21 (84.0)	No	10 (47.6)	12 (54.6)
32. Patient nights in each ward in last 30 days	No	19 (76.0)	16 (64.0)	No	4 (19.1)	1 (4.6)
33. Patient hospital ER care in last 30 days	No	24 (96.0)	21 (84.0)	No	11 (52.4)	11 (50.0)
34. Patient health care professional visits in last 30 days	No	21 (84.0)	20 (80.0)	No	7 (33.3)	9 (40.9)
35. Patient service visits in last 30 days	No	22 (88.0)	21 (84.0)	No	8 (38.1)	11 (50.0)

* Questions are deemed completed if the main parts of the question have all been completed (e.g., checkboxes), and completion rates for some questions are conditional on previous responses.

For peer review only

Supplementary File 9: Table illustrating descriptive clinical outcomes presented as median and inter-quartile ranges (baseline and 4-month follow-up).

	**Baseline**		**4-Month Follow-Up**	
	**Intervention**	**Control**	**Intervention**	**Control**
Patient participants without Cognitive Impairment				
EQ-5D-5L Index	-0.10 (-0.26, 0.23)	0.05 (-0.15, 0.41)	0.60 (0.22, 0.77)	0.60 (0.34, 0.67)
EQ-5D-5L VAS	50.0 (30.0, 55.0)	47.5 (15.0, 50.0)	55.0 (30.0, 80.0)	52.5 (35.0, 80.0)
NEADL	13 (12, 20)	17(14, 21)	20 (18, 20)	16 (13, 20)
GSE	32 (26, 36)	34 (30, 40)	29.91 (5.87)	30.70 (4.83)
CES-D	23.0 (17.0, 27.0)	22.0 (18.0, 29.0)	14 (13, 18)	19 (16, 20)
NRS pain – hip	90.0 (73.5, 100.0)	82.5 (54.0, 97.0)	20.0 (4.0, 35.0)	14.5 (10.0, 45.0)
NRS pain - body	60.0 (20.0, 90.0)	55.0 (35.0, 70.0)	17.0 (1.5, 35.0)	32.5 (14.0, 64.0)
Patient participants with Cognitive Impairment				
EQ5D Proxy Index	0.15 (-0.20, 0.34)	0.22 (0.01, 0.50)	0 (0, 0)	0.33 (0.07, 0.50)
EQ5D Proxy VAS	50.00 (15.0, 50.0)	60.0 (30.0, 75.0)	0 (0, 0)	57.5 (37.5, 67.5)
DADS-6 total (carer)	2.0 (1.0, 4.00)	1.0 (1.0, 4.0)	NA	0.0 (0.0, 7.5)
DADS-6 initiation (carer)	1.5 (0.5, 3.0)	0.0 (0.0, 1.00)	NA	0.0 (0.0, 2.5)
DADS-6 planification (carer)	0.0 (0.0, 1.0)	1.0 (0.0, 2.0)	NA	0.0 (0.0, 2.5)
DADS-6 performance (carer)	1.5 (0.5, 2.5)	1.0 (0.0, 1.0)	NA	0.0 (0.0, 2.5)
NPI severity (carer)	10 (5, 12)	3 (3, 7)	NA	5 (1, 11)
NPI distress (carer)	9 (1, 11)	1 (0, 2)	NA	1 (1, 11)
Abbey Pain Scale (carer)	6.5 (3.0, 10.0)	5.0 (5.0, 6.0)	NA	5 (4, 5)
NRS pain – hip	100.0 (70.0, 100.0)	60.0(45.0, 75.0)	NA	20 (5, 50)
NRS pain - body	35.0 (20.0, 60.0)	45.0 (20.5, 65.0)	NA	51 (26, 70)
Caregiver participants				
EQ-5D-5L Index	0.80(0.71, 1.00)	1.00 (0.77, 1.00)	0.88 (0.71, 1.00)	0.77 (0.68, 1.00)
EQ-5D-5L VAS	85.0 (80.0, 92.5)	85.0 (80.0, 95.0)	82.5 (72.5, 92.5)	77.5 (67.5, 90.0)
CESD	14.0 (12.0, 19.0)	13.5 (11.5, 19.5)	16.5 (13.5, 18.0)	13.0 (12.0, 19.0)
SCQ total	60.0 (53.0, 65.0)	63.5 (60.0, 68.0)	65.5 (58.0, 74.5)	60.0(55.0, 68.0)
SCQ recipient satisfaction	17.5 (15.5, 20.0)	18.0 (16.0, 20.0)	20.0 (17.0, 20.0)	20.0 (16.0, 20.0)
SCQ own satisfaction	19.0 (17.0, 21.0)	21.0 (18.0, 22.0)	21.0 (19.5, 23.5)	18.0 (17.5, 20.5)
SCQ consequence	26.0 (21.5, 27.0)	27.0 (22.0, 28.0)	27.0 (17.5, 32.0)	24.0 (21.0, 26.0)
Patient living in own home: n=Yes (%)	23 (95.8)	21 (91.3)	11 (100.0)	10 (100.0)
Caregiver living with participant: n=Yes (%)	16 (69.6)	14 (60.9)	11 (91.7)	7 (58.3)

CESD - Center for Epidemiologic Studies Depression Scale; DAD-6 – Disability Assessment for Dementia scale – 6 item; GSE – Generalized Self-Efficacy Scale; NA – not assessed; NEADL - Nottingham Extended Activities of Daily Living scale (NEADL); NPI - Neuropsychiatry Inventory; NRS – numerical rating scale; SCQ – Short Sense of Competence questionnaire for caregiver burden; VAS – visual analogue scale

For peer review only

Supplementary File 10: Acceptability questionnaire (participant) by question number.

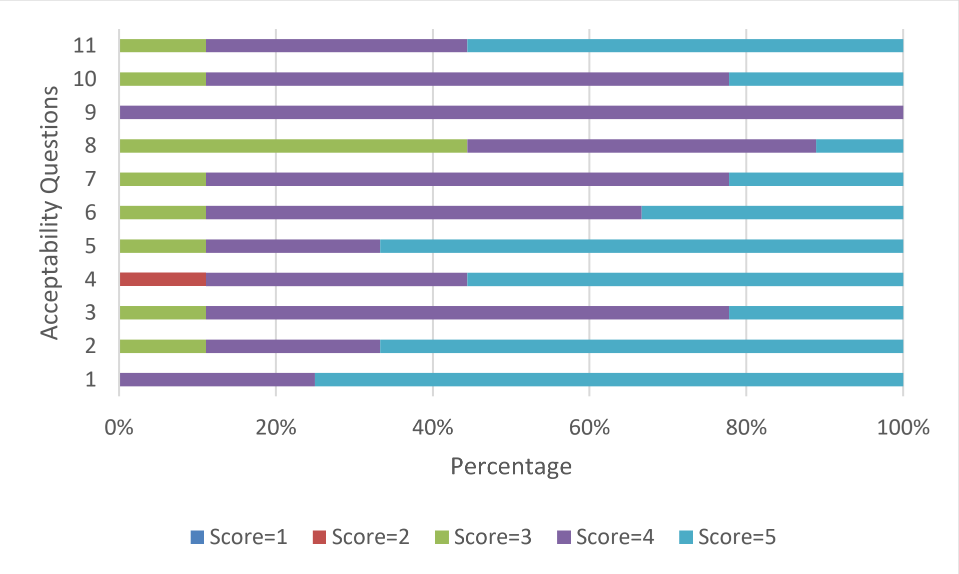

Question 1: How acceptable was the HIP HELPER in hospital training (Missing=13)
Question 2: How acceptable were the 3 HIP HELPER telephone calls? (Missing=12)
Question 3: How acceptable was the HIP HELPER Workbook? (Missing=12)
Question 4: How much effort was it to engage with the HIP HELPER in-hospital training? (Missing=12)
Question 5: How much effort did it take to engage with the HIP HELPER telephone calls? (Missing=12)
Question 6: How much effort did it take to engage with the HIP HELPER workbook? (Missing=12)
Question 7: To what extent does the HIP HELPER programme fit with your belief about recovery after a hip fracture operation? (Missing=12)
Question 8: Is the HIP HELPER programme likely to change your ability to help your friend/family member's recover after a hip fracture operation? (Missing=12)
Question 9: Does the HIP HELPER programme provide you with more confidence on your skills to help someone after a hip fracture operation? (Missing=12)
Question 10: Is it clear how the HIP HELPER intervention could help recovery after a hip fracture operation? (Missing=12)
Question 11: Did doing the HIP HELPER programme interrupt with you other priorities? (Missing=12)

Note: higher scores indicate greater acceptability.

For peer review only

Supplementary File 11: Acceptability questionnaire (caregiver) by question number.

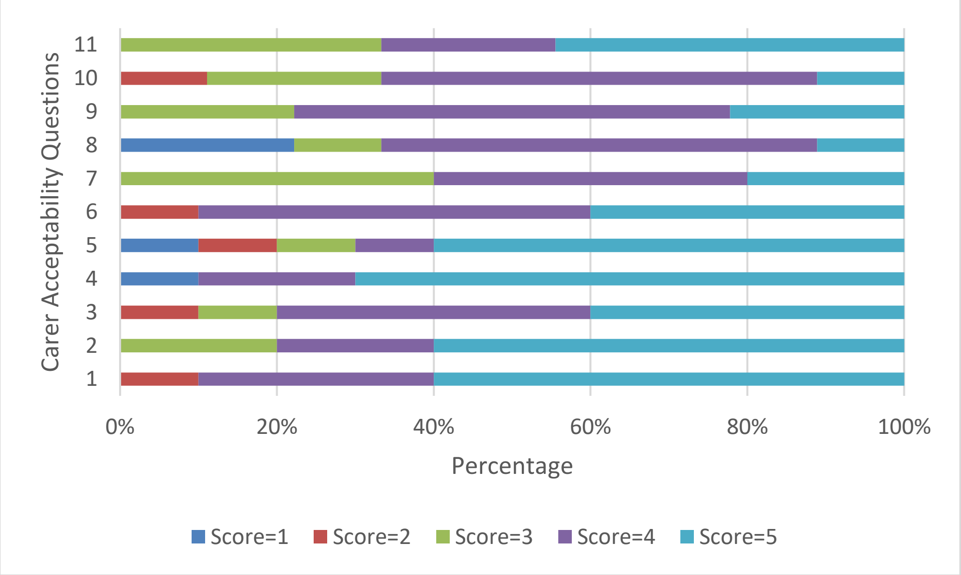

Question 1: How acceptable was the HIP HELPER in hospital training
Question 2: How acceptable were the 3 HIP HELPER telephone calls?
Question 3: How acceptable was the HIP HELPER Workbook?
Question 4: How much effort was it to engage with the HIP HELPER in-hospital training?
Question 5: How much effort did it take to engage with the HIP HELPER telephone calls?
Question 6: How much effort did it take to engage with the HIP HELPER workbook?
Question 7: To what extent does the HIP HELPER programme fit with your belief about recovery after a hip fracture operation?
Question 8: Is the HIP HELPER programme likely to change your ability to help your friend/family member's recover after a hip fracture operation?
Question 9: Does the HIP HELPER programme provide you with more confidence on your skills to help someone after a hip fracture operation?
Question 10: Is it clear how the HIP HELPER intervention could help recovery after a hip fracture operation?
Question 11: Did doing the HIP HELPER programme interrupt with you other priorities?

Note: higher scores indicate greater acceptability.

For peer review only

Supplementary File 12: Summary of safety outcomes at the end of the study for all participants.

**Adverse Event**	**Frequency**
Death	7
Falls	5
Joint infection	4
Increased pain (hip)	4
Increased pain (other joints)	4
Anxiety/depression	4
Atrial fibrillation	1
Deep wound infection	1
Wound infection	1
Skin integrity complication	1
PE	1
Stroke	1
Bowel obstruction	1
Barrett's oesophagus	1
**Total**	**36**

For peer review only

Supplementary File 13: Characteristics of qualitative investigation sample.

Person with hip fracture	Intervention	Control
N	7	3
Mean age (years)	77.85	69.33
Gender (M/F)	6 / 1	0 / 3
Ethnicity		
White British	7	3
Site (n)		
1	1	1
2	1	1
3	1	1
4	3	-
5	1	-
**Healthcare Professionals**	**Frequency**	
**Professional Role**		
Physiotherapist	4	
Occupational therapist	2	
Nurse	1	
Researcher	1	
**Site**		
1	2	
2	2	
3	1	
4	2	
5	1	

For peer review only

CONSORT checklist of information to include when reporting a pilot trial*

Section/topic and item No	Standard checklist item	Extension for pilot trials	Page No where item is reported
**Title and abstract**			
1a	Identification as a randomised trial in the title	Identification as a pilot or feasibility randomised trial in the title	Title.
1b	Structured summary of trial design, methods, results, and conclusions (for specific guidance see CONSORT for abstracts)	Structured summary of pilot trial design, methods, results, and conclusions (for specific guidance see CONSORT abstract extension for pilot trials)	Abstract.
**Introduction**			
Background and objectives:			
2a	Scientific background and explanation of rationale	Scientific background and explanation of rationale for future definitive trial, and reasons for randomised pilot trial	Introduction, Para 1-4
2b	Specific objectives or hypotheses	Specific objectives or research questions for pilot trial	Introduction, Para 5.
**Methods**			
Trial design:			
3a	Description of trial design (such as parallel, factorial) including allocation ratio	Description of pilot trial design (such as parallel, factorial) including allocation ratio	Methods, Study Design
3b	Important changes to methods after trial commencement (such as eligibility criteria), with reasons	Important changes to methods after pilot trial commencement (such as eligibility criteria), with reasons	None. Protocol Submitted
Participants:			
4a	Eligibility criteria for participants		
4b	Settings and locations where the data were collected		Methods, Eligibility Criteria
4c		How participants were identified and consented	Methods, Eligibility Criteria.
Interventions:			
5	The interventions for each group with sufficient details to allow replication, including how and when they were actually administered		Methods, Study Treatments.
Outcomes:			
6a	Completely defined prespecified primary and secondary outcome measures, including how and when they were assessed	Completely defined prespecified assessments or measurements to address each pilot trial objective specified in 2b, including how and when they were assessed	Methods, Outcome Measures + Data Collection
6b	Any changes to trial outcomes after the trial commenced, with reasons	Any changes to pilot trial assessments or measurements after the pilot trial commenced, with reasons	None.
6c		If applicable, prespecified criteria used to judge whether, or how, to proceed with future definitive trial	Protocol Submitted.

Sample size:			
7a	How sample size was determined	Rationale for numbers in the pilot trial	Methods, Sample size
7b	When applicable, explanation of any interim analyses and stopping guidelines		
Randomisation:			
Sequence generation:			
8a	Method used to generate the random allocation sequence		Methods, Randomisation + Blinding.
8b	Type of randomisation; details of any restriction (such as blocking and block size)	Type of randomisation(s); details of any restriction (such as blocking and block size)	
Allocation concealment mechanism:			
9	Mechanism used to implement the random allocation sequence (such as sequentially numbered containers), describing any steps taken to conceal the sequence until interventions were assigned		Methods, Randomisation + Blinding
Implementation:			
10	Who generated the random allocation sequence, enrolled participants, and assigned participants to interventions		Methods, Randomisation + Blinding
Blinding:			
11a	If done, who was blinded after assignment to interventions (eg, participants, care providers, those assessing outcomes) and how		Methods, Randomisation + Blinding
11b	If relevant, description of the similarity of interventions		N/A.
Analytical methods:			
12a	Statistical methods used to compare groups for primary and secondary outcomes	Methods used to address each pilot trial objective whether qualitative or quantitative	Methods, Data Analyses + Progression Criteria.
12b	Methods for additional analyses, such as subgroup analyses and adjusted analyses	Not applicable	
**Results**			
Participant flow (a diagram is strongly recommended):			Figure 1.
13a	For each group, the numbers of participants who were randomly assigned, received intended treatment, and were analysed for the primary outcome	For each group, the numbers of participants who were approached and/or assessed for eligibility, randomly assigned, received intended treatment, and were assessed for each objective	Figure 1 Results, Patient Characteristics + Treatments
13b	For each group, losses and exclusions after randomisation, together with reasons		Figure 1.
Recruitment:			

2/4

14a	Dates defining the periods of recruitment and follow-up		N/A.
14b	Why the trial ended or was stopped	Why the pilot trial ended or was stopped	
Baseline data:			
15	A table showing baseline demographic and clinical characteristics for each group		Table 1+2.
Numbers analysed:			
16	For each group, number of participants (denominator) included in each analysis and whether the analysis was by original assigned groups	For each objective, number of participants (denominator) included in each analysis. If relevant, these numbers should be by randomised group	Figure 1.
Outcomes and estimation:			
17a	For each primary and secondary outcome, results for each group, and the estimated effect size and its precision (such as 95% confidence interval)	For each objective, results including expressions of uncertainty (such as 95% confidence interval) for any estimates. If relevant, these results should be by randomised group	Supp File 9.
17b	For binary outcomes, presentation of both absolute and relative effect sizes is recommended	Not applicable	Supp File 9.
Ancillary analyses:			
18	Results of any other analyses performed, including subgroup analyses and adjusted analyses, distinguishing prespecified from exploratory	Results of any other analyses performed that could be used to inform the future definitive trial	Supp File 9.
Harms:			
19	All important harms or unintended effects in each group (for specific guidance see CONSORT for harms)		Supp File 10
19a		If relevant, other important unintended consequences	Supp File 10.
**Discussion**			
Limitations:			
20	Trial limitations, addressing sources of potential bias, imprecision, and, if relevant, multiplicity of analyses	Pilot trial limitations, addressing sources of potential bias and remaining uncertainty about feasibility	Discussion, Para 6.
Generalisability:			
21	Generalisability (external validity, applicability) of the trial findings	Generalisability (applicability) of pilot trial methods and findings to future definitive trial and other studies	Discussion, Para 3, 4 + 6
Interpretation:			
22	Interpretation consistent with results, balancing benefits and harms, and considering other relevant evidence	Interpretation consistent with pilot trial objectives and findings, balancing potential benefits and harms, and considering other relevant evidence	Discussion. Para 1
22a		Implications for progression from pilot to future definitive trial, including any proposed amendments	Conclusion. Table 3.
**Other information**			

Registration:			
23	Registration number and name of trial registry	Registration number for pilot trial and name of trial registry	Abstract + Acknowledgement + Declaration
Protocol:			
24	Where the full trial protocol can be accessed, if available	Where the pilot trial protocol can be accessed, if available	Ref. 13 + Supp. upload.
Funding:			
25	Sources of funding and other support (such as supply of drugs), role of funders		Acknowledgement + Declaration
26		Ethical approval or approval by research review committee, confirmed with reference number	Acknowledgement + Declaration

*Here a pilot trial means any randomised study conducted in preparation for a future definitive RCT, where the main objective of the pilot trial is to assess feasibility.

BMJ Open

Hospital-based caregiver intervention for people following hip fracture surgery (HIP HELPER): multi-centre randomised controlled feasibility trial with embedded qualitative study in England.

Journal:	*BMJ Open*
Manuscript ID	bmjopen-2023-073611.R2
Article Type:	Original research
Date Submitted by the Author:	14-Nov-2023
Complete List of Authors:	Smith, Toby; University of Warwick Warwick Medical School, Warwick Clinical Trials Unit; University of East Anglia, Faculty of Medicine and Health Sciences<br>Khoury, Reema; University of East Anglia, Norwich Medical School<br>Hanson, Sarah; University of East Anglia, School of Health Sciences<br>Welsh, Allie; University of East Anglia, School of Education<br>Grant, Kelly; University of East Anglia, Norwich Medical School<br>Clark, Allan; University of East Anglia, Norwich Medical School<br>Ashford, Polly-Anna; University of East Anglia, Norwich Medical School<br>Hopewell, Sally; University of Oxford, Nuffield Department of Orthopaedics, Rheumatology and Musculoskeletal Sciences<br>Pfeiffer, K; Robert Bosch Hospital, Department of Clinical Gerontology and Geriatric Rehabilitation<br>Logan, Phillipa; University of Nottingham, School of Medicine<br>Crotty, Maria; Flinders University, College of Medicine and Public Health<br>Costa, Matthew; University of Oxford, Nuffield Department of Orthopaedics, Rheumatology, and Musculoskeletal Sciences<br>Lamb, Sarah; University of Exeter, College of Medicine and Health; University of Oxford, NDORMS
**Primary Subject Heading**:	Rehabilitation medicine
Secondary Subject Heading:	Rheumatology
Keywords:	ORTHOPAEDIC & TRAUMA SURGERY, Hip < ORTHOPAEDIC & TRAUMA SURGERY, REHABILITATION MEDICINE, Nursing Care

SCHOLARONE™
Manuscripts

I, the Submitting Author has the right to grant and does grant on behalf of all authors of the Work (as defined in the below author licence), an exclusive licence and/or a non-exclusive licence for contributions from authors who are: i) UK Crown employees; ii) where BMJ has agreed a CC-BY licence shall apply, and/or iii) in accordance with the terms applicable for US Federal Government officers or employees acting as part of their official duties; on a worldwide, perpetual, irrevocable, royalty-free basis to BMJ Publishing Group Ltd ("BMJ") its licensees and where the relevant Journal is co-owned by BMJ to the co-owners of the Journal, to publish the Work in this journal and any other BMJ products and to exploit all rights, as set out in our licence.

The Submitting Author accepts and understands that any supply made under these terms is made by BMJ to the Submitting Author unless you are acting as an employee on behalf of your employer or a postgraduate student of an affiliated institution which is paying any applicable article publishing charge ("APC") for Open Access articles. Where the Submitting Author wishes to make the Work available on an Open Access basis (and intends to pay the relevant APC), the terms of reuse of such Open Access shall be governed by a Creative Commons licence – details of these licences and which Creative Commons licence will apply to this Work are set out in our licence referred to above.

Other than as permitted in any relevant BMJ Author's Self Archiving Policies, I confirm this Work has not been accepted for publication elsewhere, is not being considered for publication elsewhere and does not duplicate material already published. I confirm all authors consent to publication of this Work and authorise the granting of this licence.

For peer review only

BMJ OPEN

TITLE PAGE

Title: Hospital-based caregiver intervention for people following hip fracture surgery (HIP HELPER): multi-centre randomised controlled feasibility trial with embedded qualitative study in England.

Authors: Smith TO,[1,2] Khoury R,[2] Hanson S,[2] Welsh A,[2] Grant K,[2] Clark A,[2] Ashford P-A,[2] Hopewell S,[3] Pfeiffer K,[4] Logan PA,[5] Crotty M,[6] Costa ML,[3] Lamb SE,[7] on behalf of the HIP HELPER Study Collaborators.

Affiliations

1. Warwick Clinical Trials Unit, Warwick Medical School, University of Warwick, Coventry, UK
2. Faculty of Medicine and Health Sciences, University of East Anglia, Norwich, UK
3. Oxford Trauma and Emergency Care, Nuffield Department of Rheumatology, Orthopaedics and Musculoskeletal Sciences, University of Oxford, Oxford, UK
4. Department of Geriatric Rehabilitation, Robert-Bosch-Hospital, Stuttgart, Germany
5. School of Medicine, University of Nottingham, Nottingham, UK
6. College of Medicine and Public Health, Flinders University, Adelaide, Australia
7. Exeter Medical School, University of Exeter, Exeter, UK

Corresponding Author: Professor Toby Smith, Warwick Medical School, University of Warwick, Coventry, CV1 4AL. Email: toby.smith@uea.ac.uk

For peer review only

ABSTRACT

Objectives: To assess the feasibility of conducting a pragmatic, multi-centre randomised controlled trial (RCT) to test the clinical and cost-effectiveness of an informal caregiver training programme to support the recovery of people following hip fracture surgery.

Design: Two-arm, multi-centre, pragmatic, open, feasibility RCT with embedded qualitative study.

Setting: National Health Service (NHS) providers in five English hospitals.

Participants: Community-dwelling adults, aged 60 years and over, who undergo hip fracture surgery and their informal caregivers.

Intervention: Usual care: usual NHS care. Experimental: usual NHS care *plus* a caregiver-patient dyad training programme (HIP HELPER). This programme comprised of three, one-hour, one-to-one training sessions for a patient and caregiver, delivered by a nurse, physiotherapist or occupational therapist in the hospital setting pre-discharge. After discharge, patients and caregivers were supported through three telephone coaching sessions.

Randomisation and blinding: Central randomisation was computer generated (1:1), stratified by hospital and level of patient cognitive impairment. There was no blinding.

Main outcome measures: Data collected at baseline and four months post-randomisation included: screening logs, intervention logs, fidelity checklists, acceptability data and clinical outcomes. Interviews were conducted with a subset of participants and health professionals.

Results: 102 participants were enrolled (51 patients; 51 caregivers). Thirty-nine percent (515/1311) of patients screened were eligible. Eleven percent (56/515) of eligible patients consented to be randomised. Forty-eight percent (12/25) of the intervention group reached compliance to their allocated intervention. There was no evidence of treatment contamination. Qualitative data demonstrated the trial and HIP HELPER programme was acceptable.

Conclusions: The HIP HELPER programme was acceptable to patient-caregiver dyads and health professionals. The COVID-19 pandemic impacting on site's ability to deliver the research. Modifications are necessary to the design for a viable definitive RCT.

Trial registration number: ISRCTN13270387

Data availability statement: The data that support the findings of this study are available from the corresponding author (TS) upon reasonable request. This includes access to the full protocol, anonymised participant-level dataset and statistical code.

STRENGTHS AND LIMITATIONS OF THIS STUDY

- Mixed-method approach provided useful feasibility and acceptability data.
- Assessment of diverse measures allowed evaluation of data collection for key outcome domains.
- Participant experiences and acceptability data suggest perceived value in the HIP HELPER programme.

For peer review only

- 10% of the cohort were living with cognitive impairment; none were recruited to the qualitative sub-study.
- COVID-19 pandemic affected NHS services, which impacted on study delivery.

KEYWORDS: trauma; femoral fracture; recovery; rehabilitation; domiciliary; carer; home network; RCT

WORD COUNT: 4298 manuscript; 280 abstract

For peer review only

INTRODUCTION

Hip fracture is a serious injury for older people [1]. Approximately 80,000 people aged 60 years and over experience a fragility hip fracture in the United Kingdom (UK) annually [2]. This has an estimated combined health and social cost of over £2 billion [3].

People have frequently experienced poor recovery following hip fracture [4]. The majority never return to pre-injury levels of function [3,5]. Health-related quality of life (HRQoL) is reduced and mortality is high [5,6]. Patients also often experience repeated falls. This leads to reduced independence and confidence in self-caring skills. Approximately 20% of patients who previously lived at home move into institutional care following hip fracture [7]. For those who do return home, informal caregivers who support their friend's/family member's care needs frequently experience physical and mental stress [4]. A high caregiver burden that has previously been reported by 50%, 36%, and 26% at 1-month, 3-months, and 1-year post-surgery [8] shows the multifaceted strain perceived by at least a sub-group of hip-fracture caregivers.

People after hip fracture who return home often need help. This ranges from assistance with personal activities of daily living (ADLs) such as toileting, washing, dressing and eating, to more complex tasks such as managing money, shopping and household chores [9]. Most of this required help is provided by family members or friends. Depending on the pre-fracture status of the patient, some of these informal caregivers continue in their caregiving role, others become a first-time caregiver.

Whilst informal caregivers may be willing to support their friend/family member, they frequently feel under-skilled, and have low confidence to do so [10]. A lack of information sharing, disorganised discharge planning and unclear individual roles have been identified as challenges for patients following hip fracture and their caregivers during care transitions [11]. Teaching caregiver skills to better support patients following hip fracture may improve HRQoL and independence, whilst reducing the burden of impairment for patients and caregivers [10,12].

This study aimed to assess the feasibility of conducting a pragmatic, multi-centre, randomised controlled trial (RCT) to test the clinical and cost-effectiveness of an informal caregiver training programme to support the recovery of people following hip fracture surgery.

METHODS

The study was reported to satisfy the CONSORT extension for reporting pilot and feasibility RCTs[13]. A full protocol has been published previously [14]. The study followed the published protocol with the exception of the introduction of the optional delivery of the HIP HELPER programme through an online approach rather than face-to-face delivery. This was in response to the COVID-19 pandemic, and enacted for one participant-dyad.

Study Design

This was a feasibility study comprising of a parallel, multicentre, pragmatic RCT and embedded qualitative study. The study process evaluation results are presented in this paper.

The study flowchart is presented as **Figure 1**.

For peer review only

Eligibility Criteria

Participants were recruited from orthopaedic services in five National Health Service (NHS) hospitals in England providing hip fracture surgery. We recruited adults who previously lived in the community (not institutional care), aged 60 years and over, who had undergone hip fracture surgery, could nominate an informal caregiver and provided both patient-caregiver consent to participate. Where a patient-participant did not have capacity, agreement from a consultee was sought.

We excluded people who had acute, unstable or terminal illness or were expected by the clinical team to be discharged to a care home (residential or nursing). Caregivers were ineligible if they had an Abbreviated Mental Test Score (AMTS) [15] of less than eight.

Study Treatments

Usual NHS surgical and rehabilitation care was received by both control and intervention groups [16]. Accordingly, post-hip fracture surgery, all participants received pre-discharge care including nursing, physiotherapy, occupational therapy and social service needs-assessment (where appropriate). Patients and their caregivers in the control group, did not receive the HIP HELPER programme, with no additional in-patient or out-patient caregiver training.

The HIP HELPER intervention has been previously described [14]. In brief, this was a patient-caregiver dyad training programme. The theoretical principle behind the programme is a social learning theory [17].

In practice, people randomised to the experimental group received the usual NHS care in addition to the HIP HELPER programme. The only difference between the groups was the addition of three, 60-minute, health professional-caregiver dyad HIP HELPER training sessions, performed in the hospital setting whilst the patient was an in-patient, and three follow-up telephone calls one, three and six weeks after hospital discharge. In the in-patient sessions, participants were taught about the normal recovery process, and skills in goal-setting, pacing, activity behaviour modification and stress management. They were also taught skills on manual handling, transfers, walking and how to support people with ADLs. The follow-up telephone calls aimed to re-enforce the skills developed in the face-to-face sessions, support any set-backs in recovery and to develop longer-term goals.

Each health professional (physiotherapist, occupational therapist or nurse) who delivered the experimental intervention attended a one-day training session, which taught the components and format of the programme. To promote compliance with the treatment protocol, the Central Trial Team had regular contact with clinical team members, reviewing the first HIP HELPER sessions for intervention fidelity and held monthly meetings regarding study processes.

Data Collection

At the time of enrolment, sites checked eligibility and recorded demographic characteristics in the screening log. Baseline assessments were undertaken after consent was obtained, prior to randomisation. Data collected at baseline included: hospital admission, age, sex, ethnicity, height, weight, patient cognitive impairment assessed using the AMTS [15], past medical history, American Society of Anaesthesiologists (ASA) grade [18], side of hip fracture, operative procedure and hip fracture classification. Caregiver demographic data collected included: relationship of caregiver to patient, age, sex, ethnicity, past medical history, AMTS [15], whether they lived with the patient, employment status and experience of being a caregiver (for this patient and/or for another person).

For peer review only

Participants were followed-up at four months post-randomisation. Data were collected via postal questionnaires by the central trial team.

Outcome Measures

To answer our feasibility objectives, we assessed:

1. *Recruitment feasibility* – by screening log data on: number of potential participants and their caregivers assessed for eligibility, including reasons for exclusion/non-participation, and consented to be randomised; timing and location of approach and consent.

2. *Intervention acceptability* – by qualitative interviews with participants and health professionals; acceptability questionnaire, study attrition at the intervention phase.

3. *Intervention fidelity (healthcare professionals)* – by intervention log data on: HIP HELPER session duration, frequency, location (orthopaedic/orthogeriatric ward, rehabilitation ward or other); Quality Assurance (QA) to monitor HIP HELPER programme delivery.

4. *Intervention fidelity (caregivers)* – by caregiver HIP HELPER programme intervention logs; qualitative interviews.

5. *Randomisation acceptability* – by screening logs, eligibility assessment logs and consent forms; participant attrition; qualitative investigation.

6. *Risk of contamination* - by HIP HELPER programme log data including: QA monitoring visit checklists; delegation logs; qualitative interviews with health professionals.

7. *Completeness of outcome measures* - by completion rates (baseline and four months post-randomisation). Outcome measures collected are included in **Supplementary File 1**.

Randomisation and blinding

Randomisation was at the patient-caregiver dyad level (1:1 experimental and control groups) by stratification for: hospital and the presence of patient cognitive impairment (AMTS[15] $<$ or $\geq$ 8 points). Sites team members performed randomisation post-baseline data collection. Allocation was concealed prior to randomisation. Randomisation was computer generated, performed by site team members on a secure, online programme, centrally administered by an independent programmer at the Norwich CTU (NCTU). The randomisation sequence was generated by NCTU programmers, tested by the trial statistician.

Due to the participatory nature of the intervention, blinding participants or the site team was not possible. Senior research team members were blinded to treatment allocation for the duration of the study.

Sample Size

We aimed to recruit 120 participants (60 patients; 60 caregivers). This was considered sufficient to answer our feasibility objectives and assess the *a priori* progression criteria based on Teare et al [19] recommendations.

Data Analysis and Progression Criteria

For peer review only

Consent rates, recruitment rates, attrition, missing data rates and intervention fidelity were reported as proportions with 95% confidence intervals (CIs) presented for consent and recruitment rates. The analysis of clinical outcome measures was descriptive, reported as means and standard deviations (SD) or medians and interquartile ranges (IQR) and numbers and percentages for binary and categorical variables. No formal statistical testing was undertaken.

A ‘traffic light’ system was used as a guide for progression to a definitive trial [20]. The progression criteria were centred around recruitment, retention, intervention fidelity and contamination.

Study Monitoring

A Trial Oversight Committee (TOC) was appointed to independently review data on safety, protocol adherence and study processes.

Patient and Public Involvement

Patient involvement began during protocol development and continued throughout the study. One patient-member (not enrolled in the study) attended TOC meetings. They provided insights into the study conduct, particularly on data collection processes and helped interpret the findings to inform the study’s dissemination phase.

Participants who expressed an interest in receiving information on the findings were provided with this.

Embedded Qualitative Study

The aim of the embedded qualitative investigation was to assess the acceptability of the HIP HELPER programme and the research design from the perspective of caregiver dyads and health professionals. Its design was guided by MRC guidance for evaluating complex interventions [21-23], with the intention of understanding contextual factors influencing implementation, theorising how the HIP HELPER programme may work in practice and identifying key uncertainties to enable the programme and research design to be refined.

Six weeks after hospital discharge, caregiver dyads were invited, via a telephone call, to participate in an in-depth, semi-structured interview. They were purposively sampled by age, ethnicity, pre-fracture disability (Nottingham ADL scale (NEADL)[24]), level of cognitive health (AMTS [15]), Disability Assessment for Dementia Scale-6 [25]) and study site. For health professionals, we invited for interview those who had completed the HIP HELPER programme with at least one caregiver-dyad. This sample was purposively sampled by site location and clinical background, to ensure representation across physiotherapy, nursing and occupational therapy professions. All interviews were conducted virtually using Microsoft Teams or telephone. Our topic guide was informed by the MRC guidelines [22,23] and Sekhon’s framework of acceptability [26]. All interviews were conducted by the same researcher (AW), an experienced post-doctoral, female, qualitative researcher. AW had no role in recruitment to the study nor intervention delivery. Interviews were audio-recorded, anonymised and transcribed verbatim.

Our analysis took a two-stage approach. Firstly deductive, to assess the quality of implementation and identify contextual factors using the MRC frameworks as a guide [22,23]. An inductive approach further explored participant’s experiences and reflections on the intervention from a caregiver dyad perspective. Analysis was independently conducted by one researcher (AW) and then themed with an

For peer review only

additional two (SH, TS) using Reflexive Thematic Analysis, whereby the highly contextual nature of the data was acknowledged [27,28].

RESULTS

Patient characteristics and treatment

As a result of disruption on NHS services caused by the COVID-19 pandemic, we recruited 102 participants (51 patients; 51 caregivers) from April 2021 to February 2022.

A summary of the patient-cohort characteristics is presented in **Table 1**. Seventy-four percent (37/50) were female, with a mean age of 81.4 years in the intervention group and 77.6 years in the control group. In total, 94% (47/50) were white British or Irish. Ten patient-participants (five per group) had a AMTS [15] of less than eight, indicating cognitive impairment at baseline. The median length of hospital stay was 15 days (IQR: 10, 19) in the intervention group and 11 days in the control group (IQR: 8, 17). As **Table 1** demonstrates, people with hip fracture in the intervention group were older, with more medical co-morbidities and more frequently presented with intra-trochanteric fractures.

A summary of the caregiver-participant characteristics is presented in **Table 2**. Fifty-three percent (36/50) of the cohort were female. Mean age of caregivers in the intervention group was 66 years and 58 years in the control group. Caregivers were most frequently patient-participant's children (53%; 26/50) or a spouse (37%; 18/50). Most caregivers were not working (65%; 32/50); 20% (10/50) were in full-time work.

Feasibility outcomes

The outcomes of the progression criteria traffic-light assessment are presented in **Table 3**.

Recruitment, retention and randomisation acceptability

The CONSORT flow-chart is presented in **Figure 1**. As this illustrates, 1311 potential participants were screened. Of these, 515 (39%; 95% CI: 37% to 42%) were eligible, with 56/515 (11%; 95% CI: 8% to 13%) of eligible participant-dyads consented to participate. Five participant-dyads were withdrawn prior to randomisation. A summary of reasons for being ineligible or being eligible but not consenting are presented in **Supplementary File 2** and **Supplementary File 3**. Recruitment activity per-site is presented in **Supplementary File 4**.

At four-month follow-up, 43/51 participant-dyads (86%) (21 intervention, 22 control) remained in the study. Six participants died, one withdrew without reason; in each instance the complete dyad were withdrawn. At four-months, there were eight patient-participants with cognitive impairment, 35 without cognitive impairment. The groups were largely comparable at baseline (**Table 1**).

Intervention fidelity (health professionals)

Supplementary File 5 illustrate the delivery of the hospital-based and telephone-based HIP HELPER sessions. As summarised in **Supplementary File 6**, 12/25 participant-dyads (48%) of participant-dyads received the minimal compliance level of all three HIP HELPER in-patient sessions and one telephone

For peer review only

call. Reasons for non-compliance were: insufficient staff to deliver the intervention due to staff redeployment and interruption of service provision or visiting of participants due to the COVID-19 pandemic (n=2); patient-participant transferred from a unit (n=2); death (n=4); treatment discontinuation (n=4); or did not answer the telephone (n=3).

Table 4 illustrates that all components of the HIP HELPER programme were delivered during testing. Components which were most frequently delivered were: explanation on recovery expectations (96%; 23/25), goal-setting (92%; 22/25) and pacing and behaviour modification (92%; 22/25). Less frequently delivered components were the more functionally-demanding activities such as washing, dressing and stair and car transfers (38%; 9/25 each).

Intervention fidelity (caregivers)

Only two caregiver-participants returned their caregiver log. Accordingly, there were insufficient data to permit robust assessment of intervention fidelity from the caregiver perspective. This was therefore not analysed.

Contamination

From the qualitative investigations, case report forms for treatment received, protocol deviation reports and delegation logs of treating health professionals, there was no evidence of between-group intervention contamination.

Outcome Data Response Rate

There was limited difference in the completion of the caregiver-participant outcomes at baseline or four months in either group (**Supplementary File 7; Supplementary File 8**). However, there was a notable difference in outcome completion at four months for patients with cognitive impairment and their caregivers. Whilst 80% of caregivers in the control group completed the majority of outcomes, only two caregivers of people with cognitive impairment completed the outcomes (EQ-5D proxy only) in the intervention group. Patient-participants in the intervention group reported a higher response rate to all outcomes at four months except the NEADL[24] (**Supplementary File 7**).

Clinical Outcomes

Supplementary File 9 illustrates the descriptive clinical outcomes presented as median and IQRs for baseline and four-month follow-up. Between-group differences should be interpreted with caution given the level of missing data in both groups (**Supplementary File 7**), a potential baseline difference between the groups for age, presented co-morbidities and fracture type (**Table 1**) and underpowered analyses.

No participant, from either group, experienced a related adverse event or serious adverse event. A summary of the patient-caregiver reported adverse events is presented in **Supplementary File 10**.

For peer review only

Intervention acceptability questionnaire data indicated the HIP HELPER programme was regarded as acceptable by people with hip fracture (**Supplementary File 11**) and caregivers (**Supplementary File 12**).

Qualitative Study

Fourteen caregiver dyads were invited to be interviewed. Ten agreed to participate (Intervention: seven participants; control: three participants). All eight health professionals approached, agreed to be interviewed. **Supplementary File 13** summarises the patient-caregiver's and health professional's characteristics.

Our findings are grouped into three main themes: context, intervention delivery and study procedures.

<u>*Context*</u>

This study was conducted during the COVID-19 pandemic. Patient visitor policies and restrictions were in place. Most caregiver dyads suggested that the opportunity to visit their friend/relative was a main driver for participation.

> *"This trial helped me visit **** once or twice more during her stay in hospital than I would have been allowed to." (*Caregiver 1, Intervention Group, Male, Site 4*)*

For health professionals, allocating visiting time slots created a further challenge of obtaining consent.

> *"With visiting times, we're making sure it's the right carer coming in because it might not be them that have the slots booked that week."* (Occupational Therapist, Female, Site 1).

Changes in visitor policies/restrictions would need to be considered for any future trial.

From the perspective of health professionals, one of the most common reasons for non-participation was attributed to perceived burden on caregivers.

> *"They say, 'oh no that seems like a lot for my daughter to take on or that seems a lot for husband to do they already do enough, or I don't think they'd manage that'. So, it's that perception that they don't want to put any more burden on someone. Seems to be the main reason we find*." (Occupational Therapist, Site 1)

> *"Caregivers who work full-time or spouses who are too frail to make it into hospital just can't."* (Research Physiotherapist, Male, Site 5)

For this study, there was the added complexity of dyad recruitment, as reflected in this comment:

> *"I think getting both the caregiver and the patient consent is a bit of a headache."* (Occupational Therapist, Female, Site 1)

Initially, when health professionals approached the person with hip fracture, a key reason for decline was concerns about feelings of burden on their caregivers. Staff felt recruitment was more successful when the potential caregiver was approached first.

For peer review only

Intervention delivery

Participants perceived the workbook to be helpful in giving a sense of tangible timeframes for recovery. Goal-setting was seen as helping in pushing people out of their comfort zones and allowed them to reflect on progress.

> *"Made us gauge our progress and that he wanted our response to what goals we had. And as I said, going back through it each time we read another page, you realise that we've upped the goal and how far we've progressed"* (Caregiver, Intervention Group, Male, Site 4)

Areas of workbook refinement were identified, such as the volume of information included. Importantly some felt that the workbook did not reflect the life circumstances of younger participants and those still in work.

> "*Like, say, when I did it [the case studies], I was like how am I gonna drag this out for an hour with a patient who can just about get bed to chair*." (Physiotherapist, Male, Site 5)

The follow-up phone calls were seen as helpful. For health professionals, this addition added a rewarding element to their role.

> *"Telephone calls have been really useful. Especially for me because I work on inpatients where don't often get time to follow-up a patient, see how they are, see if there's any concerns. I suppose, from a development point of view, knowing what has worked and what hasn't."* (Physiotherapist, Male, Site 2)

For the patient-caregiver dyads, telephone calls provided encouragement and reassurance to maintain and progress activities.

> *"Reassured me that I'm doing the right things and where I should expect to be."* (Person with Hip Fracture, Intervention Group, Female, Site 4)

Participants perceived value in the telephone calls particularly in navigating additional services and support after discharge. They expressed this would have been challenging without this follow-up.

Study procedures

Respondents repeatedly acknowledged the perceived burden of completing the outcome measures.

> *"It would be a lot for say, the husband to stay at home, trying to fill in 15 pages, when the wife is not there and then they have to try to adjust living by themselves and with all this happening."* (Nurse Practitioner, Female, Site 2)

The outcome measures evaluated patient-caregiver outcomes from a biopsychosocial perspective [29]. Accordingly, some patient reported outcomes posed questions regarding an individual's ability to cope with the physical and psychosocial challenges of trauma. For some respondents, this was reported as emotionally difficult.

> *"So, when you were doing it [questionnaires] with the carer, there was a couple of times where it was uncomfortable. They might not want to say that I have low in mood in front of*

For peer review only

someone. It was quite upsetting for the carer to divulge that information with you, or to bring up things from their past as well." (Physiotherapist, Male, Site 3)

DISCUSSION

The findings from this feasibility study indicate that the HIP HELPER intervention was acceptable to patients, informal caregivers and health professionals but the trial design requires further development to ensure feasibility. Modifications should be made on promoting intervention adherence, prioritising outcome measures to test future effectiveness of data completeness and exploring strategies to support the recruitment of patients with cognitive impairment.

The completion of data from the outcomes at four months was lower than anticipated. This was particularly for participants with cognitive impairment. The qualitative study indicated that participants found the number of outcome measures challenging to complete and future study could better discern what outcome measures are important to people following hip fracture and their caregivers [30]. Streamlining should be made to determine the outcomes which are most valuable to participants and clinical commissioners. A second major modification relates to the acceptability of randomisation. Only 11% of eligible participants were randomised. The qualitative study indicated this may have been because patient-participants did not wish to 'burden their caregiver' with the study when they were initially approached and so declined participation. We originally designed the study approach pre-COVID 19 with an initial approach occurring when both patient and caregiver were together, during visiting hours. This was to facilitate a collaborative decision between the dyad, rather than one member deciding participation. However, due to COVID-19 restrictions on visiting, this was not possible. The qualitative study indicated that the originally planned approach should have been more successful. We recommend that both members of the dyad should be approached simultaneously in future trials, to mitigate such low conversion to randomisation.

A written, information guide about rehabilitation, recovery goals and caregiver responsibilities in the home has been previously reported as valuable to other populations [31]. The findings of this study indicate that whilst the addition of this intervention was beneficial, the HIP HELPER workbook received mixed views from participants. The level of detail, degree of context and order of material covered in the HIP HELPER workbook was considered by many participants as too great. Equally the qualitative findings suggested that the current materials were not representative of all patients and caregivers, most notably younger people who sustain a hip fracture. Further patient and public consultation with the research and clinical hip fracture community is needed to modify this workbook and associated digital offerings of this material.

Approximately 40% of people who sustain a hip fracture present with dementia [32]. Whilst previous authors have acknowledged potential challenges in recruiting people with dementia to drug trials [33], no studies have explored recruitment expectations or strategies to address low recruitment to non-pharmacological interventions [34]. We anticipated recruiting 20 patient-participants with cognitive impairment. In total, 10 participants were recruited with mild cognitive impairment. The qualitative findings suggest that offering further support to research site members who approach patients with cognitive impairment and their caregivers, to promote skills conveying study information, may be beneficial. Furthermore, given the poor response rate in four-month outcome data for these participants, consideration on the appropriateness of the current instruments used and model of delivery of outcome battery for people with cognitive impairment and their caregiver, should be considered in future trials of this population [35].

For peer review only - http://bmjopen.bmj.com/site/about/guidelines.xhtml

Previous evidence suggests that health professionals have been inflexible about people with hip fracture and their caregivers to discuss care plans, when these do happen [36-38]. The qualitative study highlighted that those participants who received the HIP HELPER programme appreciated the contact and opportunity to explore skills and knowledge for early recover and caregiver support following hip fracture. The addition of the telephone calls was reported as offering beneficial, additional, post-discharge support in a flexible approach. However, intervention fidelity was lower than anticipated. Unfortunately, it is difficult to separate the challenges which COVID-19 placed on research conduct and service provision and challenges in delivering the HIP HELPER programme in a non-health crisis. Sites were challenged in delivering the intervention due to staffing, patient transfers, visiting restrictions and earlier than planned discharges. This impacted on fidelity of the 'full' HIP HELPER programme to all participants. Deeper exploration on modification to intervention delivery and what components are 'core' ingredients to the programme to estimate compliance thresholds would be warranted.

This study presented with strengths and limitations. A notable strength was the ability to recruit over 100 participants from five NHS organisations during the 2020 COVID-19 pandemic. Whilst a short-fall of 10 participant-dyads, given the challenges in managing site opening and research conduct during the COVID-19 pandemic, the ability to undertake this was considered a success. In the absence of COVID-19 (or such like) restrictions on visiting, patient flow and research activity in NHS settings, we would anticipate that this impact would be negated in future trials of this population. Secondly, although we planned to assess whether caregivers adopted their caregiving knowledge from the intervention into the home environment, only two participants returned these data. This was a major limitation and resulted in an inability to answer an *a prior* progression criterion. The findings from the qualitative study and acceptability questionnaires may suggest carry-over of the intervention into practice. However, we acknowledge that this does not offer the granularity of detail which the original caregiver log would have conveyed. Finally, the follow-up rates and data competition for clinical outcomes were low. Accordingly, it was not possible to confidently assess for a signal of efficacy in the experimental intervention. Further modifications in what and how clinical outcome data are collected should be considered as part of the following work to improve the feasibility of this trial design.

CONCLUSIONS

The HIP HELPER programme was acceptable to participants and health professions. Further modifications to the trial design are needed to ensure feasibility. These findings will form the basis of reflection and refinement to the trial design to test the clinical and cost-effectiveness of the programme in addition to understand the scalability and pathway to implementation.

For peer review only

ACKNOWLEDGEMENTS AND DECLARATIONS

Collaborators: The HIP HELPER Study Collaborators: Penny Clifford (Norfolk, PPI Representative), Lis Freeman (Norfolk, PPI Representative), Rene Gray (Principal Investigator – James Paget University Hospital NHS Trust), Yan Cunningham (Principal Investigator – City Hospitals Sunderland NHS Foundation Trust), Sarah Langford (Principal Investigator - Northumbria Healthcare NHS Foundation Trust), Dr Mark Baxter (Principal Investigator - University Hospital Southampton NHS Foundation Trust), Jessica Pawson – (Principal Investigator - Barts Health NHS Trust), Melissa Taylor (James Paget University Hospital NHS Trust), Anna Mellows (James Paget University Hospital NHS Trust), Kate Lacey (James Paget University Hospital NHS Trust), Alex Herring (City Hospital Sunderland NHS Foundation Trust), Diane Williams (Northumbria Healthcare NHS Foundation Trust), Anna Cromie (Northumbria Healthcare NHS Foundation Trust), Gail Menton (Northumbria Healthcare NHS Foundation Trust), Warren Corbett (University Hospital Southampton NHS Foundation Trust), Helen Jowett (University Hospital Southampton NHS Foundation Trust), Vishwanath Joshi (Barts Health NHS Trust), Maninderpal Matharu (Barts Health NHS Trust), Maria Baggot (University Hospital Southampton NHS Foundation Trust),and David Barker (University Hospital Southampton NHS Foundation Trust).

Oversight Committee Membership: TOC Members: Associate Professor Susan Dutton (Chair; University of Oxford, UK), Professor Opinder Sahota (University of Nottingham, UK), Dr Katie Sheehan (Kings College London, UK).

Authors' Contributors: TS, SHo, SHa, PA, AC, KG, KP, PL, MCo, SEL, MCr researched the topic and devised the study. TS, SHa, PA, RK, AC, KG, SHo, AW, KP, PL, MCo, SEL, MCr provided the first draft of the manuscript. AC provided statistical oversight. TS, SHa, PA, RK, AC, KG, AW, SHo, KP, PL, MCo, SEL, MCr contributed equally to manuscript preparation. TS acts a guarantor.

Funding Statement: This project is funded by the National Institute for Health and Care Research (NIHR) under its Research for Patient Benefit (RfPB) Programme (Grant Reference Number NIHR200731). SL role in this study was supported by the National Institute for Health and Care Research Exeter Biomedical Research Centre. The views expressed are those of the author(s) and not necessarily those of the NIHR or the Department of Health and Social Care.

Competing Interests Statement: None declared.

Disclaimer: MCo and SHo are supported by the National Institute for Health Research (NIHR) Oxford Biomedical Research Centre (BRC). SL is supported by the National Institute for Health and Care Research Exeter Biomedical Research Centre. The views expressed are those of the author(s) and not necessarily those of the NHS, the NIHR or the Department of Health and Social Care.

Ethics Approval: North East - Newcastle & North Tyneside 1 Research Ethics Committee (20/NE/0213) Date: 16 March 2021.

Trial Sponsor: University of East Anglia (Research Sponsor, Research and Innovation Services, The Registry, University of East Anglia, Norwich, NR4 7TJ; Email: researchsponsor@uea.ac.uk) The views expressed are those of the author(s) and not necessarily those of the sponsor.

Provenance and Peer Review: Not commissioned; externally peer reviewed.

Data Availability Statement:
Data are available upon reasonable request. This included access to the full protocol, anonymised participant level dataset and statistical code. Access to the de-identified dataset for purposes of

For peer review only

research other than this study, would be at the discretion of the Chief Investigator, Professor Toby Smith and Norwich CTU. Requests for the de-identified dataset generated during the current study should be made to the Chief Investigator, Professor Toby Smith (email: toby.o.smith@warwick.ac.uk) or Norwich CTU (NorwichCTU@uea.ac.uk). Professor Toby Smith and Norwich CTU will consider requests once the main results from the study have been published up until 31 December 2028.

Patient consent for publication: Not required.

For peer review only

FIGURE AND TABLE LEGENDS

Table 1: Demographic characteristics of the patient participants at baseline.

Table 2: Demographic characteristics of the caregiver participants at baseline.

Table 3: Progression criteria traffic-light summary table.

Table 4: Table illustrating the frequency to-which the components of the HIP HELPER intervention were delivered to participants.

Figure 1: CONSORT diagram reporting the flow of patient-caregiver participants in the HIP HELPER study.

Supplementary File 1: Clinical outcome data collected for patient-participants and caregiver-participants at baseline and four-months post-randomisation.

Supplementary File 2: Reasons for ineligibility in the HIP HELPER study across the five sites.

Supplementary File 3: Reasons for eligible participants not consenting to the HIP HELPER study across the five sites.

Supplementary File 4: Recruitment rate presented by each of the five HIP HELPER sites.

Supplementary File 5: Table illustrating the number of participant-dyads that attended intervention sessions.

Supplementary File 6: Intervention fidelity by site (shown below are those that achieved intervention fidelity).

Supplementary File 7: Table illustrating the data completion of clinical outcome scores (baseline and 4-month follow-up).

Supplementary File 8: Carer Resource Utilisation in Dementia questionnaire completion statistics at baseline and 4-months.

Supplementary File 9: Table illustrating descriptive clinical outcomes presented as median and inter-quartile ranges (baseline and 4-month follow-up).

Supplementary File 10: Acceptability questionnaire (participant) by question number.

Supplementary File 11: Acceptability questionnaire (caregiver) by question number.

Supplementary File 12: Summary of safety outcomes at the end of the study for all participants.

Supplementary File 13: Characteristics of qualitative investigation sample.

For peer review only

REFERENCES

1. National Hip Fracture Database 2023. Assessment benchmark summary 2022. Accessed: 16th February 2023. Available at: https://www.nhfd.co.uk/tables
2. Mitchell P, Bateman K. Dementia, falls and fractures. Integrated approaches to improve quality and reduce costs. UK: Novartis; 2012.
3. Dyer SM, Dyer SM, Crotty M, Fairhall N, Magaziner J, Beaupre LA, Cameron ID, Sherrington C; Fragility Fracture Network (FFN) Rehabilitation Research Special Interest Group. A critical review of the long-term disability outcomes following hip fracture. *BMC Geriatr* 2016;**16**:158.
4. Lawler K, Taylor NF, Shields N. Involving family members in physiotherapy for older people transitioning from hospital to the community: a qualitative analysis. *Disabil Rehabil* 2015;**37**:2061-9.
5. Johnell O, Kanis JA. An estimate of the worldwide prevalence, mortality and disability associated with hip fracture. *Osteopor Int* 2004;**15**:897-902.
6. Griffin XL, Parsons N, Achten J, Fernandez M, Costa ML. Recovery of health-related quality of life in a United Kingdom hip fracture population. The Warwick Hip Trauma Evaluation--a prospective cohort study. *Bone Joint J* 2015;**97-B**:372-82.
7. Nurmi I, Narinen A, Lüthje P, Tanninen S. Cost analysis of hip fracture treatment among the elderly for the public health services: a 1-year prospective study in 106 consecutive patients. *Arch Orthop Trauma Surg* 2003;**123**:551-4.
8. Ariza-Vega P, Ortiz-Piña M, Kristensen MT, Castellote-Caballero Y, Jiménez-Moleón JJ. High perceived caregiver burden for relatives of patients following hip fracture surgery. *Disabil Rehabil* 2017;**16**:1-8
9. Wimo A, Wetterholm AL, Mastey V, Winblad B. Evaluation of the resource utilization and caregiver time in Anti-dementia drug trials - a quantitative battery. In: Wimo A, Karlsson G, Jönsson B, Winblad B (eds). The Health Economics of dementia, 1998. Wiley's, London, UK
10. Saletti-Cuesta L, Tutton E, Langstaff D, Willett K. Understanding informal carers' experiences of caring for older people with a hip fracture: a systematic review of qualitative studies. *Disabil Rehabil* 2018;**40**:740-50.
11. Asif M, Cadel L, Kuluski K, Everall AC, Guilcher SJT. Patient and caregiver experiences on care transitions for adults with a hip fracture: a scoping review. *Disabil Rehabil* 2020;**42**:3549-58.
12. Giosa JL, Stolee P, Dupuis SL, Mock SE, Santi SM. Examination of family caregiver experiences during care transitions of older adults. *Can J Aging* 2014;**33**:137-53.
13. Eldridge SM, Chan CL, Campbell MJ, Bond CM, Hopewell S, Thabane L, Lancaster GA; PAFS consensus group. CONSORT 2010 statement: extension to randomised pilot and feasibility trials. *BMJ* 2016;**355**:i5239.
14. Smith T, Clark L, Khoury R, Man MS, Hanson S, Welsh A, Clark A, Hopewell S, Pfeiffer K, Logan P, Crotty M, Costa M, Lamb SE. A feasibility study to assess the design of a multicentre randomized controlled trial of the clinical and cost-effectiveness of a caregiving intervention for people following hip fracture surgery. *Bone Jt Open* 2021;**2**:909-20.
15. Hodkinson KM. Evaluation of a mental test score for assessment of mental impairment in the elderly. *Age Ageing* 1972;**4**:233-8.
16. National Institute for Health and Care Excellence. Hip fracture: management. Clinical guideline CG124 - 2023. Available at: https://www.nice.org.uk/Guidance/CG124 Accessed on: 16 February 2023.
17. Bandura A. Self-efficacy: towards a unifying theory of behavioural change. *Psychol Rev* 1977; **84**:191–215.
18. Saklad M. Grading of patients for surgical procedures. *Anesthesiology* 1941;**2**:281-4.

19. Teare MD, Dimairo M, Shephard N, Hayman A, Whitehead A, Walters SJ. Sample size requirements to estimate key design parameters from external pilot randomised controlled trials: a simulation study. *Trials* 2014;**15**:264
20. Avery KN, Williamson PR, Gamble C, O'Connell Francischetto E, Metcalfe C, Davidson P, Williams H, Blazeby JM; members of the Internal Pilot Trials Workshop supported by the Hubs for Trials Methodology Research. Informing efficient randomised controlled trials: exploration of challenges in developing progression criteria for internal pilot studies. *BMJ Open* 2017;**7**:e013537.
21. Craig P, Dieppe P, Macintyre S, Michie S, Nazareth I, Petticrew M; Medical Research Council Guidance. Developing and evaluating complex interventions: the new Medical Research Council guidance. *BMJ* 2008;**337**:a1655.
22. Moore GF, Audrey S, Barker M, Bond L, Bonell C, Hardeman W, et al. Process evaluation of complex interventions: Medical Research Council guidance. *BMJ* 2015;**350**:h1258.
23. Skivington K, Matthews L, Simpson SA, Craig P, Baird J, Blazeby JM, Boyd KA, Craig N, French DP, McIntosh E, Petticrew M, Rycroft-Malone J, White M, Moore L. A new framework for developing and evaluating complex interventions: update of Medical Research Council guidance. *BMJ* 2021;**374**:n2061.
24. Gladman JR, Lincoln NB, Adams SA. Use of the extended ADL scale with stroke patients. *Age Ageing* 1993;**22**:419–24.
25. de Rotrou J, Wu YH, Hugonot-Diener L, Thomas-Antérion C, Vidal JS, Plichart M, Rigaud AS, Hanon O. DAD-6: A 6-Item version of the Disability Assessment for Dementia scale which may differentiate Alzheimer's disease and mild cognitive impairment from controls. *Dement Geriatr Cogn Disord* 2012;**33**:210-8.
26. Sekhon M, Cartwright M, Francis JJ. Acceptability of healthcare interventions: an overview of reviews and development of a theoretical framework. *BMC Health Serv Res* 2017;**17**:88.
27. Braun V, Clarke V. Using thematic analysis in psychology. *Qual Res Psychol* 2006;**3**:77-101.
28. Braun V, Clarke V. One size fits all? What counts as quality practice in (reflexive) thematic analysis? *Qual Res Psychol* 2020;**3**:1-25.
29. Engel GL. The need for a new medical model: a challenge for biomedicine. *Science* 1977;**196**:129-36.
30. Treweek S, Miyakoda V, Burke D, Shiely F. Getting it wrong most of the time? Comparing trialists' choice of primary outcome with what patients and health professionals want. *Trials* 2022;**23**:537.
31. Schiller C, Franke T, Belle J, Sims-Gould J, Sale J, Ashe MC. Words of wisdom - patient perspectives to guide recovery for older adults after hip fracture: a qualitative study. *Patient Prefer Adherence* 2015;**9**:57-64.
32. Seitz DP, Adunuri N, Gill SS, Rochon PA. Prevalence of dementia and cognitive impairment among older adults with hip fractures. *J Am Med Direct Assoc* 2011;**12**:556-64.
33. Cooper C, Ketley D, Livingston G. Systematic review and meta-analysis to estimate potential recruitment to dementia intervention studies. *Int J Geriatr Psychiatry* 2014;**29**:515-25.
34. Brambilla M, Parra MA, Della Sala S, Alemanno F, Pomati S. Challenges to recruitment of participants with MCI in a multicentric neuropsychological study. *Aging Clin Exp Res* 2021;**33**:2007-10.
35. Kramer JM, Schwartz A. Reducing barriers to patient-reported outcome measures for people with cognitive impairments. *Arch Phys Med Rehabil* 2017;**98**:1705-15.
36. Elliott J, Forbes D, Chesworth BM, Ceci C, Stolee P. Information sharing with rural family caregivers during care transitions of hip fracture patients. *Int J Integr Care* 2014;**14**:e018.

For peer review only

37. Glenny C, Stolee P, Sheiban L, Jaglal S. Communicating during care transitions for older hip fracture patients: family caregiver and health care provider's perspectives. *Int J Integr Care* 2013;**13**:e044.
38. Nahm ES, Resnick B, Orwig D, Magaziner J, Degrezia M. Exploration of informal caregiving following hip fracture. *Geriatr Nurs* 2010;**31**:254-62.

For peer review only

Table 1: Demographic characteristics of the patient participants at baseline.

	Intervention (N=25)	Control (N=25)
Gender: n (%)		
Male	4 (16.0)	9 (36.0)
Female	21 (84.0)	16 (64.0)
Age in years: mean (SD)	81.4 (8.1)	77.6 (8.6)
Ethnicity: n (%)		
White British	22 (88.0)	23 (92.0)
White Irish	1 (4.0)	1 (4.0)
Indian	0	1 (4.0)
Bangladeshi	2 (8.0)	0
Height (cm): mean (SD)	164.2 (10.6)	166.81 (10.0)
Weight (kg): mean (SD)	65.1 (13.9)	72.6 (17.2)
BMI: mean (SD)	24.1 (4.5)	26.1 (6.2)
AMTS score: median (IQR)	10 (9, 10)	10 (9, 10)
AMTS category		
Cognitive Impairment (score<8)	5 (20.0)	5 (20.0)
No Cognitive Impairment (score≥8)	20 (80.0)	20 (80.0)
Side of hip fracture: n (%)		
Left	10 (40.0)	15 (60.0)
Right	15 (60.0)	10 (40.0)
Hip Fracture classification: n (%)		
Intra-capsular	14 (56.0)	17 (68.0)
Intertrochanteric	8 (32.0)	5 (20.0)
Sub-trochanteric	3 (12.0)	3 (12.0)
Operative Procedure: n (%)		
Hemiarthroplasty	10 (41.7)	15 (60.0)
THR	2 (8.3)	1 (4.0)
Cannulated screws	3 (12.5)	1 (4.0)
DHS	4 (16.7)	5 (20.0)
Intramedullary device	5 (20.8)	3 (12.0)
Missing	1	0
Length of hospital stay (days): median (IQR)	15 (10, 19)	11 (8, 17)
ASA Grade: median (IQR)	3 (3, 3)	3 (2, 3)
Missing	1	1
Current Medical Diagnoses: n (%)		
Cardiac	8 (32.0)	5 (20.0)
Asthma	2 (8.0)	1 (4.0)
COPD	6 (24.0)	7 (28.0)
Hypertension	12 (48.0)	14 (56.0)
Diabetes	2 (8.0)	5 (20.0)
Stroke	0	2 (8.0)
Cancer	7 (28.0)	3 (12.0)
Osteoarthritis	5 (20.0)	3 (12.0)
Low back pain	4 (16.0)	3 (12.0)
Depression	0	0
Anxiety	0	0
Dementia	2 (8.0)	2 (8.0)
Other	12 (48.0)	11 (44.0)

AMTS – Abbreviated Mental Test Score; ASA - American Society of Anesthesiologists OPD – chronic obstructive pulmonary disease; BMI – body mass index; cm – centimetres; DHS – dynamic hip screw; IQR – inter-quartile range; kg – kilograms; SD – standard deviation; THR – total hip replacement

For peer review only

Table 2: Demographic characteristics of the caregiver participants at baseline.

	Intervention (N=25)	Control (N=25)
Gender: n (%)		
Male	14 (56.0)	9 (37.5)
Female	11 (44.0)	15 (62.5)
Missing	0	1
Age in years: mean (SD)	66.2 (13.6)	57.7 (12.9)
Missing	1	2
Ethnicity: n (%)		
White British	20 (80.0)	23 (95.8)
White Irish	1 (4.0)	0
White Other	1 4.0)	1 (4.2)
Mixed - Other	1 (4.0)	0
Bangladeshi	2 (8.0)	0
Missing	0	1
AMTS score: median (IQR)	10 (10, 10)	10 (10, 10)
Missing	0	1
Relationship to participant: n (%)		
Spouse	10 (40.0)	8 (33.3)
Daughter/Son	13 (52.0)	13 (54.2)
Grandchild	0	1 (4.2)
Other	2 (8.0)	2 (8.3)
Missing	0	1
Caregiver living with participant: n (%)		
Yes	16 (69.6%)	14 (60.9%)
No	7 (30.4%)	9 (39.1%)
Missing	2	2
Occupation: n (%)		
Not working	17 (68.0)	15 (62.5)
Part-time	3 (12.0)	4 (16.7)
Full-time	5 (20.0)	5 (20.8)
Missing	0	1
Current Medical Diagnoses: n (%)		
Cardiac	2 (8.0)	1 (4.0)
Asthma	0	4 (16.0)
COPD	0	0
Hypertension	2 (8.0)	1 (4.0)
Diabetes	1 (4.0)	2 (8.0)
Stroke	0	0
Cancer	1 (4.0)	0
Osteoarthritis	3 (12.0)	1 (4.0)
Low back pain	3 (12.0)	4 (16.0)
Depression	0	3 (12.0)
Anxiety	0	6 (24.0)
Other	3 (12.0)	2 (8.0)

AMTS – Abbreviated Mental Test Score; COPD – chronic obstructive pulmonary disease; SD – standard deviation

For peer review only

Table 3: Progression criteria traffic-light summary table.

	Green (Go)	Amber (Amend)	Red (Stop)	Judgement
**Recruitment**	> 40% of patients screened would be eligible	30% to 40% would be eligible	< 30% would be eligible	39% participants were eligible
**Randomisation acceptability**	> 40% of eligible patients consent to be randomised	20% to 40% would be randomised	< 20% would be randomised	11% of eligible participants were randomised
**Intervention fidelity (healthcare professionals)**	> 70% of participants compliant with their allocated intervention as randomised	50% to 70% received intervention as randomised	< 50% received intervention as randomised	48% received 'complete intervention' as randomised, limited by COVID-19
**Intervention fidelity (caregivers)**	> 90% of participants adopted HIP HELPER intervention post-discharge	60% to 90% adopted HIP HELPER post-discharge	< 60% adopted HIP HELPER post-discharge	Unable to assess with insufficient caregiver logs
**Contamination**	< 5% of participants in either group received majority of their allocated treatment crossover	5% to 10% of participants crossover	> 10% of participants crossover	0% evidence of contamination

For peer review only

Table 4: Table illustrating the frequency to-which the components of the HIP HELPER intervention were delivered to participants.

Item	Intervention (N=25)			
	Session 1 N (%)	Session 2 N (%)	Session 3 N (%)	At least one occurrence during Sessions 1-3
Practical skills-transfers:				
Bed to chair	16 (66.7)	13 (68.4)	8 (57.1)	20 (83.3)
Toilet	4 (16.7)	7 (36.8)	4 (28.6)	12 (50.0)
Walking and walking aids	15 (62.5)	12 (63.2)	9 (64.3)	19 (79.2)
In/out bed	16 (66.7)	12 (63.2)	8 (57.1)	20 (83.3)
Car	3 (12.5)	6 (31.6)	2 (14.3)	9 (37.5)
Stairs	3 (12.5)	6 (31.6)	3 (21.4)	9 (37.5)
Goal Setting theory	21 (87.5)	13 (68.4)	4 (28.6)	22 (91.7)
Goal setting practice	15 (62.5)	15 (79.0)	6 (42.9)	22 (91.7)
Pacing and behaviour theory	20 (83.3)	9 (47.4)	6 (42.9)	22 (91.7)
Pacing and behaviour task	13 (54.2)	10 (52.6)	5 (35.7)	18 (75.0)
Expectations of recovery pathways	22 (91.7)	9 (47.4)	5 (35.7)	23 (95.8)
Practical skills:				
Washing	1 (4.2)	7 (36.8)	1 (7.1)	9 (37.5)
Dressing	1 (4.2)	9 (47.4)	1 (7.1)	9 (37.5)
Caregiver:				
Management discussion	3 (12.5)	7 (36.8)	14 (100)	18 (75.0)
Pacing discussion	4 (16.7)	6 (31.6)	10 (71.4)	16 (66.7)
Case scenario discussion	1 (4.2)	3 (15.8)	9 (64.3)	12 (50.0)
Provision and discussion on HIP HELPER manual	15 (62.5)	4 (21.1)	10 (71.4)	22 (91.7)
Confirmation of HIP HELPER telephone calls	0	0	13 (92.9)	13 (54.2)
Other 1: (“pain relief”, “lack of hip precautions”, “caregiver questions”, “equipment ordering”)	1 (4.2)	2 (10.5)	1 (7.1)	2 (8.3)
Other 2: (“equipment ordering”)	0	0	1 (7.1)	1 (4.2)
Other 3: (“sleep”)	0	0	1 (7.1)	1 (4.2)

Note: Number of planned sessions not performed were: one Session 1, six Session 2, 11 Session 3; Missing data not given, percentages are out of non-missing data.

For peer review only

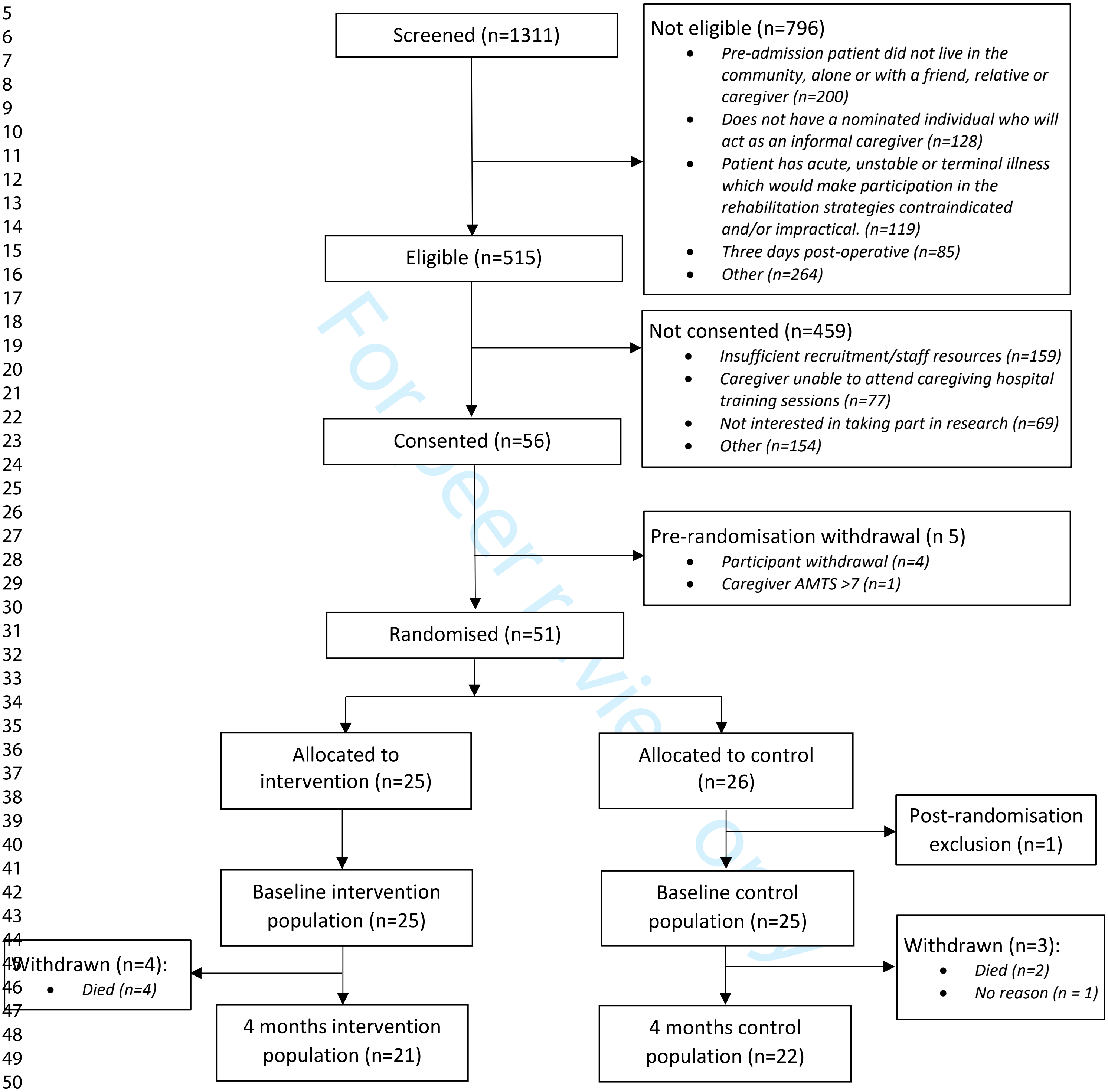
Screened (n=1311)
Not eligible (n=796)
Pre-admission patient did not live in the community, alone or with a friend, relative or caregiver (n=200)
Does not have a nominated individual who will act as an informal caregiver (n=128)
Patient has acute, unstable or terminal illness which would make participation in the rehabilitation strategies contraindicated and/or impractical. (n=119)
Three days post-operative (n=85)
Other (n=264)
Eligible (n=515)
Not consented (n=459)
Insufficient recruitment/staff resources (n=159)
Caregiver unable to attend caregiving hospital training sessions (n=77)
Not interested in taking part in research (n=69)
Other (n=154)
Consented (n=56)
Pre-randomisation withdrawal (n 5)
Participant withdrawal (n=4)
Caregiver AMTS >7 (n=1)
Randomised (n=51)
Allocated to intervention (n=25)
Allocated to control (n=26)
Post-randomisation exclusion (n=1)
Baseline intervention population (n=25)
Baseline control population (n=25)
Withdrawn (n=4):
Died (n=4)
Withdrawn (n=3):
Died (n=2)
No reason (n = 1)
4 months intervention population (n=21)
4 months control population (n=22)

Supplementary File 1: Clinical outcome data collected for patient-participants and caregiver-participants at baseline and four-months post-randomisation.

Patients without cognitive impairment:

- EQ-5D-5L
- Nottingham Activities of Daily Living Scale (NEADL)
- General Self-Efficacy questionnaire
- Center for Epidemiologic Studies Depression Scale (CES-D)
- Numerical rating scale (NRS) for pain (hip and whole body)
- Complications and adverse events including mortality

For all caregivers:

- EQ-5D-5L
- CES-D
- Short Sense of Competence Questionnaire for caregiver burden (SCQ-16)
- Resource Utilization in Dementia questionnaire
- Complications and adverse events including mortality
- Patient and caregiver residential status

PLUS for caregivers of patients with cognitive impairment

- EQ-5D-5L proxy
- Disability Assessment for Dementia Scale-6 (DADS-6) functional score
- Neuropsychiatry Inventory (NPI)
- Abbey Pain Scale

iew only

Supplementary File 2: Reasons for ineligibility in the HIP HELPER study across the five sites.

Site 1	Site 2	Site 3	Site 4	Site 5	Total	Screening	
37	38	11	83	31	200	Pre-admission patient did not live in the community, alone or with a friend, relative or caregiver.	Ineligible Reason
51	22	2	16	37	128	Does not have a nominated individual who will act as an informal caregiver.	
45	12	7	22	33	119	Patient has acute, unstable or terminal illness which would make participation in the rehabilitation strategies contraindicated and/or impractical.	
-	-	-	85	-	85	3+ days post op	
12	1	5	9	27	54	Patients expected by the clinical team to be discharged to a care home (residential or nursing) after their hospital admission	
15	1	1	17	9	43	Patient under age of 65 years	
7	4	-	29	1	41	Patient not willing or able to provide consent or assent depending on the level of cognitive impairment.	
8	1	6	15	8	38	Other	
8	4	-	21	-	33	Caregiver not willing or able to provide consent	
3	2	-	12	2	19	Not undergone hip fracture surgery	
-	-	-	11	2	13	Missing reason	
-	1	-	4	4	9	Participant has significant difficulties reading and/or comprehending English	
-	-	-	2	7	9	Patient under age of 65 years	
-	1	-	-	2	3	Individual caregivers unable to understand written English or have access to a translator.	
1	-	1	-	-	2	Principal (main) caregiver has AMTS score of less than 8	
					**796**	**Total ineligible**	

For peer review only

Supplementary File 3: Reasons for eligible participants not consenting to the HIP HELPER study across the five sites.

Site 1	Site 2	Site 3	Site 4	Site 5	Total	Not Consented	
47	26	15	43	27	158	Insufficient recruitment staff resources	Eligible but not consented
10	25	2	16	23	76	Caregiver unable to attend caregiving hospital training sessions	
15	11	6	27	11	70	Not interested in taking part in research	
6	9	1	25	14	55	Other	
21	-	3	13	1	38	Leaving the area	
3	13	1	7	4	28	Missing Reason	
19	-	-	-	1	19	Outlier ward	
-	-	-	-	-	4	Does NOT want to be randomised to receive caregiver intervention	
-	3	1	-	4	4	Persistent post-operative confusion	
-	1	1	-	-	2	Does not have a nominated individual who will act as an informal caregiver.	
-	-	-	-	-	2	Does NOT want to be randomised to receive control (usual care)	
-	-	-	1	2	2	Caregiver not willing or able to provide consent	
-	1	-	-	1	1	Participant has significant difficulties reading and/or comprehending English	
					**459**	**Total not consented**	
-	-	-	-	-	-	Post-operative complication	Not Randomised
	-	-	-	-	1	Persistent post-operative confusion	
-	-	-	-	-	-	Participant no longer wants to take part in research	
-	-	-	-	-	-	Unable to approach caregiver	
-	-	-	-	-	-	Insufficient recruitment staff resources	
2	1	-	-	1	4	Withdrawn	
					**5**	**Total not randomised**	

For peer review only

Supplementary File 4: Recruitment rate presented by each of the five HIP HELPER sites.

Site	Date Opened	Number of Recruited Participants											Total
		April 2021	May 2021	June 2021	July 2021	August 2021	Sept 2021	Oct 2021	Nov 2021	Dec 2021	Jan 2022	Feb 2022	
**Site 1**	07Apr2021	1	3	0	1	1	1	3	0	0			10
**Site 2**	19Apr2021		1	2	2	0	1	2	0	0	0	0	8
**Site 3**	15Jun2021				1	0	0	0	1	1	0	2	5
**Site 4**	29Jun2021				3	1	2	2	0	2	2	1	13
**Site 5**	05Aug2021					0	3	3	4	2	1	2	15
**TOTAL**		**1**	**4**	**2**	**7**	**2**	**7**	**10**	**5**	**5**	**3**	**5**	**51**

For peer review only

Supplementary File 5: Table illustrating the number of participant-dyads that attended intervention sessions.

	Hospital-Based Session			Telephone Booster Calls		
	Session 1 N (%)	Session 2 N (%)	Session 3 N (%)	Telephone 1 N (%)	Telephone 2 N (%)	Telephone 3 N (%)
Yes	24 (96.0)	19 (76.0)	14 (56.0)	13 (54.2)	14 (58.3)	11 (47.8)
No	1 (4.0)	6 (24.0)	11 (44.0)	11 (45.8)	10 (41.7)	12 (52.2)
Missing	0	0	0	1	1	2

For peer review only

Supplementary File 6: Intervention fidelity by site (shown below are those that achieved intervention fidelity).

	Site Number					All Sites
	**1**	**2**	**3**	**4**	**5**	
Intervention fidelity n (%)	2 (40.0)	2 (50.0)	0	3 (50.0)	5 (62.5)	12 (48.0)

N.B. fidelity = 3 in-patient sessions and >= 1 telephone calls

For peer review only

Supplementary File 7: Table illustrating the data completion of clinical outcome scores (baseline and 4-month follow-up).

	Baseline (N; %)		4-Month Follow-up (N; %)	
	Intervention	Control	Intervention	Control
Patient participants without Cognitive Impairment (n=41)				
EQ-5D-5L Index	20 (100)	19 (95.0)	14 (70.0)	11 (57.9)
EQ-5D-5L VAS	19 (95.0)	18 (90.0)	14 (70.0)	12 (63.2)
NEADL	15 (75.0)	17 (85.0)	5 (27.8)	9 (52.9)
GSE	19 (95.0)	19 (95.0)	11 (61.1)	10 (58.8)
CES-D	17 (85.0)	16 (80.0)	11 (61.1)	9 (52.9)
NRS pain – hip	20 (100)	18 (90.0)	12 (66.7)	10 (58.8)
NRS pain - body	19 (95.0)	19 (95.0)	12 (66.7)	10 (58.8)
Patient participants with Cognitive Impairment (n=10)				
EQ5D Proxy Index	5 (100)	5 (100)	2 (40.0)	4 (80.0)
EQ5D Proxy VAS	5 (100)	5 (100)	2 (40.0)	4 (80.0)
DADS-6 total (carer)	3 (60.0)	5 (100)	0	4 (80.0)
DADS-6 initiation (carer)	4 (80.0)	5 (100)	0	4 (80.0)
DADS-6 planification (carer)	3 (60.0)	5 (100)	0	4 (80.0)
DADS-6 performance (carer)	4 (80.0)	5 (100)	0	4 (80.0)
NPI severity (carer)	5 (100)	5 (100)	0	3 (60.0)
NPI distress (carer)	5 (100)	5 (100)	0	3 (60.0)
Abbey Pain Scale (carer)	2 (40.0)	5 (100)	0	3 (60.0)
NRS pain – hip	4 (80.0)	4 (80.0)	0	4 (80.0)
NRS pain - body	4 (80.0)	4 (80.0)	0	4 (80.0)
Caregiver participants (n=51)				
EQ-5D-5L Index	24 (96.0)	23 (92.0)	10 (47.6)	11 (50.0)
EQ-5D-5L VAS	24 (96.0)	23 (92.0)	12 (57.1)	12 (54.6)
CESD	20 (80.0)	20 (80.0)	12 (57.1)	9 (40.9)
SCQ total	23 (92.0)	20 (80.0)	12 (57.1)	11 (50.0)
SCQ recipient satisfaction	24 (96.0)	22 (88.0)	12 (57.1)	12 (54.6)
SCQ own satisfaction	23 (92.0)	21 (84.0)	12 (57.1)	12 (54.6)
SCQ consequence	24 (96.0)	23 (92.0	12 (57.1)	11 (50.0)
Patient living in own home: n (%)	24 (96.0)	23 (92.0)	11 (52.4)	11 (50.0)
Caregiver living with participant: n (%)	23 (92.0)	23 (92.0)	12 (57.1)	12 (54.5)

CESD - Center for Epidemiologic Studies Depression Scale; DAD-6 – Disability Assessment for Dementia scale – 6 item; GSE – Generalized Self-Efficacy Scale; NA – not assessed; NEADL - Nottingham Extended Activities of Daily Living scale (NEADL); NPI - Neuropsychiatry Inventory; NRS – numerical rating scale; SCQ – Short Sense of Competence questionnaire for caregiver burden; VAS – visual analogue scale
NB: Deaths prior to 4 month follow-up were assigned zero response accounting for 40% response rate for 2 participants in the patient participants with cognitive impairment responses.

For peer review only

Supplementary File 8: Carer Resource Utilisation in Dementia questionnaire completion statistics at baseline and 4-months

C-RUD Question	Conditional on previous question responses	Baseline		Conditional on previous question responses	4-Months	
		Intervention N=25 n (%)	Control N=25 n (%)		Intervention N=21 n (%)	Control N=22 n (%)
1. Age	No	24 (96.0)	23 (92.0)	No	12 (57.1)	12 (54.6)
2. Gender	No	24 (96.0)	23 (92.0)	No	12 (57.1)	12 (54.6)
3. Relationship to patient	No	23 (92.0)	23 (92.0)	No	12 (57.1)	12 (54.6)
4. Children living with you	No	24 (96.0)	23 (92.0)	No	12 (57.1)	12 (54.6)
5. Do you live with the patient	No	23 (92.0)	23 (92.0)	No	12 (57.1)	12 (54.6)
6. Other caregivers involved	No	23 (92.0)	23 (92.0)	No	12 (57.1)	12 (54.6)
7. Your caring contribution level	No	23 (92.0)	23 (92.0)	No	12 (57.1)	12 (54.6)
8. Sleep in last 30 days	No	24 (96.0)	21 (84.0)	No	12 (57.1)	12 (54.6)
9a. Hours per day assisting patient with tasks such as dressing in last 30 days	No	23 (92.0)	20 (80.0)	No	10 (47.6)	11 (50.0)
9b. Days assisting patient with tasks such as dressing in last 30 days	No	23 (92.0)	20 (80.0)	No	10 (47.6)	11 (50.0)
10a. Hours per day assisting patient with tasks such as shopping in last 30 days	No	23 (92.0)	20 (80.0)	No	12 (57.1)	11 (50.0)
10b. Days assisting patient with tasks such as shopping in last 30 days	No	22 (88.0)	21 (84.0)	No	12 (57.1)	11 (50.0)
11a. Hours per day supervising patient in last 30 days	No	21 (84.0)	21 (84.0)	No	11 (52.4)	12 (54.6)
11b. Days supervising patient in last 30 days	No	21 (84.0)	21 (84.0)	No	11 (52.4)	12 (54.6)
12.Work for pay	No	24 (96.0)	23 (92.0)	No	12 (57.1)	11 (50.0)
13. Stop/reduce working	Yes (N=30)	17 (100)	13 (100)	Yes (N=7)	2 (100)	5 (100)
14. Change of job/working situation	Not Assessed in Baseline C-RUD			Yes (N=7)	2 (100)	5 (100)
15. Hours of paid work per week	Yes (N=17)	7 (100)	10 (100)	Yes (N=1)	1 (100)	0
16. Hours of patient care paid per week	Yes (N=17)	7 (100)	10 (100)	Yes (N=1)	1 (100)	0
17. Hours cut for carer responsibilities in last 30 days	Yes (N=17)	7 (100)	10 (100)	Yes (N=1)	1 (100)	0
18a. Work days missed	No	6 (24.0)	11 (44.0)	Yes (N=7)	2 (100)	3 (60.0)
18b. Part work days missed	No	7 (28.0)	11 (44.0)	Yes (N=7)	2 (100)	5 (100)
19. Stop/reduce working	Not Assessed in Baseline C-RUD			Yes (N=1)	1 (100)	0

For peer review only

20. Admitted to hospital in last 30 days	No	24 (96.0)	23 (92.0)	Yes (N=23)	12 (100)	10 (90.9)
21. Nights in each ward in last 30 days	Yes (N=50)	23 (92.0)	23 (92.0)	Yes (N=23)	2 (16.7)	1 (9.1)
22. Hospital ER care in last 30 days	No	23 (92.0)	23 (92.0)	Yes (N=23)	12 (100)	9 (81.8)
23. Health care professional visits in last 30 days	No	24 (96.0)	22 (88.0)	Yes (N=23)	10 (83.3)	10 (90.9)
24. Current medications	No	22 (88.0)	18 (72.0)	No	8 (38.1)	8 (36.4)
25. Patient change of living accommodation since last visit	Not Assessed in Baseline C-RUD			No	12 (57.1)	11 (50.0)
26. Patient current living accommodation	No	24 (96.0)	23 (92.0)	Yes (N=0)	0	0
27. Date living change occurred	Not Assessed in Baseline C-RUD			Yes (N=0)	0	0
28. Reason for living change	Not Assessed in Baseline C-RUD			Yes (N=0)	0	0
29. Who patient lives with	No	24 (96.0)	23 (92.0)	Not Assessed in Follow-Up C-RUD		
30. Patient temporary accommodation in last 30 days	No	7 (28.0)	6 (24.0)	Yes (N=23)	8 (38.1)	5 (45.5)
31. Patient admitted to hospital in last 30 days	No	23 (92.0)	21 (84.0)	No	10 (47.6)	12 (54.6)
32. Patient nights in each ward in last 30 days	No	19 (76.0)	16 (64.0)	No	4 (19.1)	1 (4.6)
33. Patient hospital ER care in last 30 days	No	24 (96.0)	21 (84.0)	No	11 (52.4)	11 (50.0)
34. Patient health care professional visits in last 30 days	No	21 (84.0)	20 (80.0)	No	7 (33.3)	9 (40.9)
35. Patient service visits in last 30 days	No	22 (88.0)	21 (84.0)	No	8 (38.1)	11 (50.0)

* Questions are deemed completed if the main parts of the question have all been completed (e.g., checkboxes), and completion rates for some questions are conditional on previous responses.

For peer review only

Supplementary File 9: Table illustrating descriptive clinical outcomes presented as median and inter-quartile ranges (baseline and 4-month follow-up).

	**Baseline**		**4-Month Follow-Up**	
	**Intervention**	**Control**	**Intervention**	**Control**
Patient participants without Cognitive Impairment				
EQ-5D-5L Index	-0.10 (-0.26, 0.23)	0.05 (-0.15, 0.41)	0.60 (0.22, 0.77)	0.60 (0.34, 0.67)
EQ-5D-5L VAS	50.0 (30.0, 55.0)	47.5 (15.0, 50.0)	55.0 (30.0, 80.0)	52.5 (35.0, 80.0)
NEADL	13 (12, 20)	17(14, 21)	20 (18, 20)	16 (13, 20)
GSE	32 (26, 36)	34 (30, 40)	29.91 (5.87)	30.70 (4.83)
CES-D	23.0 (17.0, 27.0)	22.0 (18.0, 29.0)	14 (13, 18)	19 (16, 20)
NRS pain – hip	90.0 (73.5, 100.0)	82.5 (54.0, 97.0)	20.0 (4.0, 35.0)	14.5 (10.0, 45.0)
NRS pain - body	60.0 (20.0, 90.0)	55.0 (35.0, 70.0)	17.0 (1.5, 35.0)	32.5 (14.0, 64.0)
Patient participants with Cognitive Impairment				
EQ5D Proxy Index	0.15 (-0.20, 0.34)	0.22 (0.01, 0.50)	0 (0, 0)	0.33 (0.07, 0.50)
EQ5D Proxy VAS	50.00 (15.0, 50.0)	60.0 (30.0, 75.0)	0 (0, 0)	57.5 (37.5, 67.5)
DADS-6 total (carer)	2.0 (1.0, 4.00)	1.0 (1.0, 4.0)	NA	0.0 (0.0, 7.5)
DADS-6 initiation (carer)	1.5 (0.5, 3.0)	0.0 (0.0, 1.00)	NA	0.0 (0.0, 2.5)
DADS-6 planification (carer)	0.0 (0.0, 1.0)	1.0 (0.0, 2.0)	NA	0.0 (0.0, 2.5)
DADS-6 performance (carer)	1.5 (0.5, 2.5)	1.0 (0.0, 1.0)	NA	0.0 (0.0, 2.5)
NPI severity (carer)	10 (5, 12)	3 (3, 7)	NA	5 (1, 11)
NPI distress (carer)	9 (1, 11)	1 (0, 2)	NA	1 (1, 11)
Abbey Pain Scale (carer)	6.5 (3.0, 10.0)	5.0 (5.0, 6.0)	NA	5 (4, 5)
NRS pain – hip	100.0 (70.0, 100.0)	60.0(45.0, 75.0)	NA	20 (5, 50)
NRS pain - body	35.0 (20.0, 60.0)	45.0 (20.5, 65.0)	NA	51 (26, 70)
Caregiver participants				
EQ-5D-5L Index	0.80(0.71, 1.00)	1.00 (0.77, 1.00)	0.88 (0.71, 1.00)	0.77 (0.68, 1.00)
EQ-5D-5L VAS	85.0 (80.0, 92.5)	85.0 (80.0, 95.0)	82.5 (72.5, 92.5)	77.5 (67.5, 90.0)
CESD	14.0 (12.0, 19.0)	13.5 (11.5, 19.5)	16.5 (13.5, 18.0)	13.0 (12.0, 19.0)
SCQ total	60.0 (53.0, 65.0)	63.5 (60.0, 68.0)	65.5 (58.0, 74.5)	60.0(55.0, 68.0)
SCQ recipient satisfaction	17.5 (15.5, 20.0)	18.0 (16.0, 20.0)	20.0 (17.0, 20.0)	20.0 (16.0, 20.0)
SCQ own satisfaction	19.0 (17.0, 21.0)	21.0 (18.0, 22.0)	21.0 (19.5, 23.5)	18.0 (17.5, 20.5)
SCQ consequence	26.0 (21.5, 27.0)	27.0 (22.0, 28.0)	27.0 (17.5, 32.0)	24.0 (21.0, 26.0)
Patient living in own home: n=Yes (%)	23 (95.8)	21 (91.3)	11 (100.0)	10 (100.0)
Caregiver living with participant: n=Yes (%)	16 (69.6)	14 (60.9)	11 (91.7)	7 (58.3)

CESD - Center for Epidemiologic Studies Depression Scale; DAD-6 – Disability Assessment for Dementia scale – 6 item; GSE – Generalized Self-Efficacy Scale; NA – not assessed; NEADL - Nottingham Extended Activities of Daily Living scale (NEADL); NPI - Neuropsychiatry Inventory; NRS – numerical rating scale; SCQ – Short Sense of Competence questionnaire for caregiver burden; VAS – visual analogue scale

For peer review only

Supplementary File 10: Acceptability questionnaire (participant) by question number.

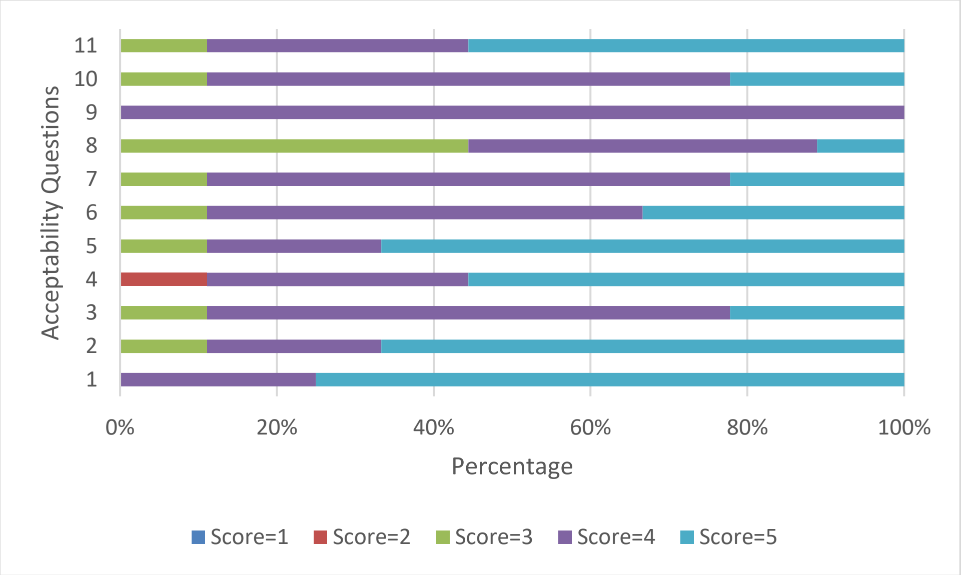

Question 1: How acceptable was the HIP HELPER in hospital training (Missing=13)
Question 2: How acceptable were the 3 HIP HELPER telephone calls? (Missing=12)
Question 3: How acceptable was the HIP HELPER Workbook? (Missing=12)
Question 4: How much effort was it to engage with the HIP HELPER in-hospital training? (Missing=12)
Question 5: How much effort did it take to engage with the HIP HELPER telephone calls? (Missing=12)
Question 6: How much effort did it take to engage with the HIP HELPER workbook? (Missing=12)
Question 7: To what extent does the HIP HELPER programme fit with your belief about recovery after a hip fracture operation? (Missing=12)
Question 8: Is the HIP HELPER programme likely to change your ability to help your friend/family member's recover after a hip fracture operation? (Missing=12)
Question 9: Does the HIP HELPER programme provide you with more confidence on your skills to help someone after a hip fracture operation? (Missing=12)
Question 10: Is it clear how the HIP HELPER intervention could help recovery after a hip fracture operation? (Missing=12)
Question 11: Did doing the HIP HELPER programme interrupt with you other priorities? (Missing=12)

Note: higher scores indicate greater acceptability.

Supplementary File 11: Acceptability questionnaire (caregiver) by question number.

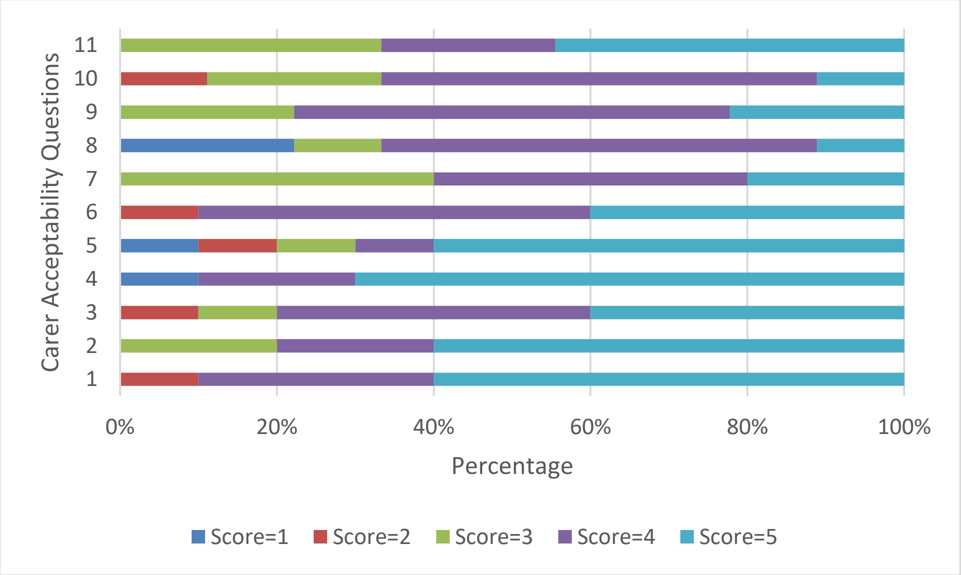

Question 1: How acceptable was the HIP HELPER in hospital training
Question 2: How acceptable were the 3 HIP HELPER telephone calls?
Question 3: How acceptable was the HIP HELPER Workbook?
Question 4: How much effort was it to engage with the HIP HELPER in-hospital training?
Question 5: How much effort did it take to engage with the HIP HELPER telephone calls?
Question 6: How much effort did it take to engage with the HIP HELPER workbook?
Question 7: To what extent does the HIP HELPER programme fit with your belief about recovery after a hip fracture operation?
Question 8: Is the HIP HELPER programme likely to change your ability to help your friend/family member's recover after a hip fracture operation?
Question 9: Does the HIP HELPER programme provide you with more confidence on your skills to help someone after a hip fracture operation?
Question 10: Is it clear how the HIP HELPER intervention could help recovery after a hip fracture operation?
Question 11: Did doing the HIP HELPER programme interrupt with you other priorities?

Note: higher scores indicate greater acceptability.

For peer review only

Supplementary File 12: Summary of safety outcomes at the end of the study for all participants.

**Adverse Event**	**Frequency**
Death	7
Falls	5
Joint infection	4
Increased pain (hip)	4
Increased pain (other joints)	4
Anxiety/depression	4
Atrial fibrillation	1
Deep wound infection	1
Wound infection	1
Skin integrity complication	1
PE	1
Stroke	1
Bowel obstruction	1
Barrett's oesophagus	1
**Total**	**36**

For peer review only

Supplementary File 13: Characteristics of qualitative investigation sample.

Person with hip fracture	Intervention	Control
N	7	3
Mean age (years)	77.85	69.33
Gender (M/F)	6 / 1	0 / 3
Ethnicity		
White British	7	3
Site (n)		
1	1	1
2	1	1
3	1	1
4	3	-
5	1	-
**Healthcare Professionals**	**Frequency**	
**Professional Role**		
Physiotherapist	4	
Occupational therapist	2	
Nurse	1	
Researcher	1	
**Site**		
1	2	
2	2	
3	1	
4	2	
5	1	

For peer review only

CONSORT checklist of information to include when reporting a pilot trial*

Section/topic and item No	Standard checklist item	Extension for pilot trials	Page No where item is reported
**Title and abstract**			
1a	Identification as a randomised trial in the title	Identification as a pilot or feasibility randomised trial in the title	Title.
1b	Structured summary of trial design, methods, results, and conclusions (for specific guidance see CONSORT for abstracts)	Structured summary of pilot trial design, methods, results, and conclusions (for specific guidance see CONSORT abstract extension for pilot trials)	Abstract.
**Introduction**			
Background and objectives:			
2a	Scientific background and explanation of rationale	Scientific background and explanation of rationale for future definitive trial, and reasons for randomised pilot trial	Introduction, Para 1-4
2b	Specific objectives or hypotheses	Specific objectives or research questions for pilot trial	Introduction, Para 5.
**Methods**			
Trial design:			
3a	Description of trial design (such as parallel, factorial) including allocation ratio	Description of pilot trial design (such as parallel, factorial) including allocation ratio	Methods, Study Design
3b	Important changes to methods after trial commencement (such as eligibility criteria), with reasons	Important changes to methods after pilot trial commencement (such as eligibility criteria), with reasons	None. Protocol Submitted
Participants:			
4a	Eligibility criteria for participants		Methods, Eligibility Criteria
4b	Settings and locations where the data were collected		
4c		How participants were identified and consented	Methods, Eligibility Criteria.
Interventions:			
5	The interventions for each group with sufficient details to allow replication, including how and when they were actually administered		Methods, Study Treatments.
Outcomes:			
6a	Completely defined prespecified primary and secondary outcome measures, including how and when they were assessed	Completely defined prespecified assessments or measurements to address each pilot trial objective specified in 2b, including how and when they were assessed	Methods, Outcome Measures + Data Collection
6b	Any changes to trial outcomes after the trial commenced, with reasons	Any changes to pilot trial assessments or measurements after the pilot trial commenced, with reasons	None.
6c		If applicable, prespecified criteria used to judge whether, or how, to proceed with future definitive trial	Protocol Submitted.

Sample size:			
7a	How sample size was determined	Rationale for numbers in the pilot trial	Methods, Sample size
7b	When applicable, explanation of any interim analyses and stopping guidelines		
Randomisation:			
Sequence generation:			
8a	Method used to generate the random allocation sequence		Methods, Randomisation + Blinding.
8b	Type of randomisation; details of any restriction (such as blocking and block size)	Type of randomisation(s); details of any restriction (such as blocking and block size)	
Allocation concealment mechanism:			
9	Mechanism used to implement the random allocation sequence (such as sequentially numbered containers), describing any steps taken to conceal the sequence until interventions were assigned		Methods, Randomisation + Blinding
Implementation:			
10	Who generated the random allocation sequence, enrolled participants, and assigned participants to interventions		Methods, Randomisation + Blinding
Blinding:			
11a	If done, who was blinded after assignment to interventions (eg, participants, care providers, those assessing outcomes) and how		Methods, Randomisation + Blinding
11b	If relevant, description of the similarity of interventions		N/A.
Analytical methods:			
12a	Statistical methods used to compare groups for primary and secondary outcomes	Methods used to address each pilot trial objective whether qualitative or quantitative	Methods, Data Analyses + Progression Criteria.
12b	Methods for additional analyses, such as subgroup analyses and adjusted analyses	Not applicable	
**Results**			
Participant flow (a diagram is strongly recommended):			Figure 1.
13a	For each group, the numbers of participants who were randomly assigned, received intended treatment, and were analysed for the primary outcome	For each group, the numbers of participants who were approached and/or assessed for eligibility, randomly assigned, received intended treatment, and were assessed for each objective	Figure 1 Results, Patient Characteristics + Treatments
13b	For each group, losses and exclusions after randomisation, together with reasons		Figure 1.
Recruitment:			

14a	Dates defining the periods of recruitment and follow-up		N/A.
14b	Why the trial ended or was stopped	Why the pilot trial ended or was stopped	
Baseline data:			
15	A table showing baseline demographic and clinical characteristics for each group		Table 1 + 2.
Numbers analysed:			
16	For each group, number of participants (denominator) included in each analysis and whether the analysis was by original assigned groups	For each objective, number of participants (denominator) included in each analysis. If relevant, these numbers should be by randomised group	Figure 1.
Outcomes and estimation:			
17a	For each primary and secondary outcome, results for each group, and the estimated effect size and its precision (such as 95% confidence interval)	For each objective, results including expressions of uncertainty (such as 95% confidence interval) for any estimates. If relevant, these results should be by randomised group	Supp File 9.
17b	For binary outcomes, presentation of both absolute and relative effect sizes is recommended	Not applicable	Supp File 9.
Ancillary analyses:			
18	Results of any other analyses performed, including subgroup analyses and adjusted analyses, distinguishing prespecified from exploratory	Results of any other analyses performed that could be used to inform the future definitive trial	Supp File 9.
Harms:			
19	All important harms or unintended effects in each group (for specific guidance see CONSORT for harms)		Supp File 10
19a		If relevant, other important unintended consequences	Supp File 10.
**Discussion**			
Limitations:			
20	Trial limitations, addressing sources of potential bias, imprecision, and, if relevant, multiplicity of analyses	Pilot trial limitations, addressing sources of potential bias and remaining uncertainty about feasibility	Discussion, Para 6.
Generalisability:			
21	Generalisability (external validity, applicability) of the trial findings	Generalisability (applicability) of pilot trial methods and findings to future definitive trial and other studies	Discussion, Para 3, 4 + 6.
Interpretation:			
22	Interpretation consistent with results, balancing benefits and harms, and considering other relevant evidence	Interpretation consistent with pilot trial objectives and findings, balancing potential benefits and harms, and considering other relevant evidence	Discussion. Para 1
22a		Implications for progression from pilot to future definitive trial, including any proposed amendments	Conclusion. Table 3.
**Other information**			

Registration:			
23	Registration number and name of trial registry	Registration number for pilot trial and name of trial registry	Abstract + Acknowledgement + Declaration
Protocol:			
24	Where the full trial protocol can be accessed, if available	Where the pilot trial protocol can be accessed, if available	Ref. 13 + Supp. Upload.
Funding:			
25	Sources of funding and other support (such as supply of drugs), role of funders		Acknowledgement + Declaration
26		Ethical approval or approval by research review committee, confirmed with reference number	Acknowledgement + Declaration

*Here a pilot trial means any randomised study conducted in preparation for a future definitive RCT, where the main objective of the pilot trial is to assess feasibility.

4/4
